# Flash Communication: A Metal-First Approach to Ruthenium Complexes of a Boryl-Centered POBOP Pincer Ligand

**DOI:** 10.1021/acs.organomet.6c00078

**Published:** 2026-04-24

**Authors:** Jovanny J. Contreras, Mason W. Bayles, Nattamai Bhuvanesh, Lori A. Watson, Oleg V. Ozerov

**Affiliations:** † Department of Chemistry, 14736 Texas A&M University, College Station, Texas 77842, United States; ‡ Department of Chemistry, 6884 Earlham College, 801 National Rd W, Richmond, Indiana 47374, United States

## Abstract

Boryl/bis­(phosphine) complexes of (POBOP)Ru were assembled by first preparing intermediates containing two bidentate phosphino/phenoxide ligands coordinated to Ru, followed by their reactions with BH_3_·SMe_2_.

## Graphical abstract

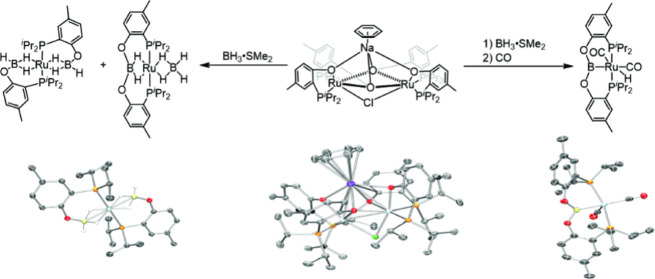

Pincer^1–3^ ligands containing a central boryl moiety attached to a transition metal (X_2_B–M) have attracted particular interest owing to the strong donor ability of boryl as a ligand^4,5^ and to the availability of the empty orbital at boron for unique reactivity pathways.^6,7^ Yamashita and Nozaki reported the first example of a PBP pincer with Ir,^8^ which was subsequently used with other metals, including Ru in **A** and **B** (Figure 1).^9–18^ Carborane-based PBP ligands have also been reported (**C**),^19^ and our group has explored diarylboryl-centered PBP complexes (e.g., **D**), with Rh and Ir.^6,20–22^ The synthesis of all of these examples, and of most other pincer complexes in general, typically proceeds via the preparation of the propincer molecule containing the three future donor atoms, which is then treated with a transition metal precursor. We recently reported on an alternative strategy, wherein the phosphine-containing portions of the eventual pincer complex are first ligated to a metal center before introducing the main group reagent that delivers the central donor atom and completes the pincer (Figure 1).^23^ This approach has precedent in the work of Braunstein.^24,25^ Having previously^23^ demonstrated the efficacy of this approach in the synthesis of a series of Rh, Ir (e.g., **E**), and Pd complexes, we now report on its application to a group 8 metal Ru.

**Figure 1:**
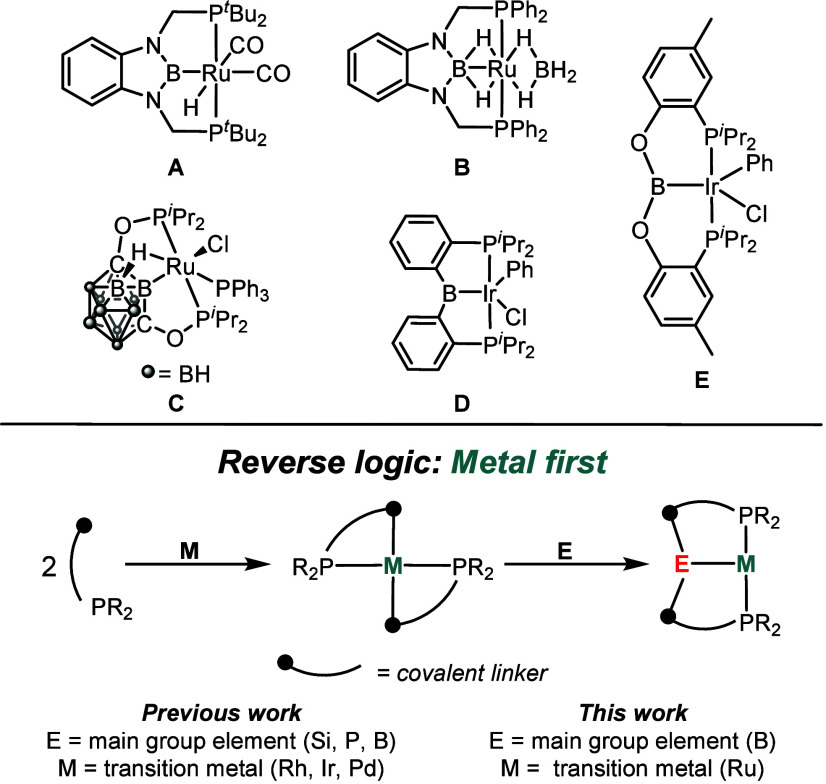
PBP complexes of Ru and the reverse synthesis strategy.

We began by attempting to synthesize well-defined precursors containing two “P–O” units from the phosphinophenolate **1** bound to Ru (Scheme 1). Treatment of [(COD)­RuCl_2_]_n_ with **1** (2 equiv per Ru) in toluene at 100 °C for 42 h,^26^ yielded a single product **2** that could be isolated as an analytically pure red solid in 47% yield via recrystallization from diethyl ether.^27^ This reaction stoichiometry might be expected to result in the formation of “(PO)_2_Ru”. However, the latter is only a 14-electron L_2_RuX_2_ fragment that is geometrically restricted from attaining the 18-electron count solely via bridging oxygens. The Caulton group,^28^ and subsequently the Schneider group,^29^ showed that monomeric 14-electron L_2_RuX_2_ pincer complexes (PNP)­RuCl can be isolated, but ostensibly require larger *t*-Bu groups on the phosphorus. Instead, bimetallic **2** adopts a structure with two (PO)_2_Ru units sharing one pair of bridging oxygens, complemented by the retention of a unit of NaCl. An XRD study on a crystal of **2-C**_**6**_ **H**_**6**_ grown in the absence of ether shows (Figure 2) that that the chloride bridging the two Ru centers brings the electron count to 18 at each Ru, while the sodium cation interacts with all four oxygens of the P–O units, as well as a molecule of benzene. We assume that Et_2_O can take the place of benzene in **2-C**_**6**_ **H**_**6**_. Not surprisingly, when supplied with an excess of a strongly binding ligand such as CO, the reaction of **1** with [(COD)­RuCl_2_]_n_ instead gave monomeric, 18-electron compound (PO)_2_Ru­(CO)_2_ **3**. While **2** displayed a pair of doublets (74.0 and 69.6 ppm, *J*_P–P_ = 35 Hz) in the ^31^P­{^1^H} NMR spectrum, **3** gave rise to a single resonance at 69.8 ppm. Two intense bands of similar intensity were noted in the IR spectrum of **3** (ν_CO_ = 2024 and 1957 cm^–1^), consistent with the *cis*-disposition of the carbonyl ligands. An XRD study (Figure 2) confirmed the expected structure of **3**. The Ru–O bond lengths in **2-C**_**6**_ **H**_**6**_ and **3** are typical of phosphinophenolate units bound to Ru.^30,31^

**Scheme 1:**
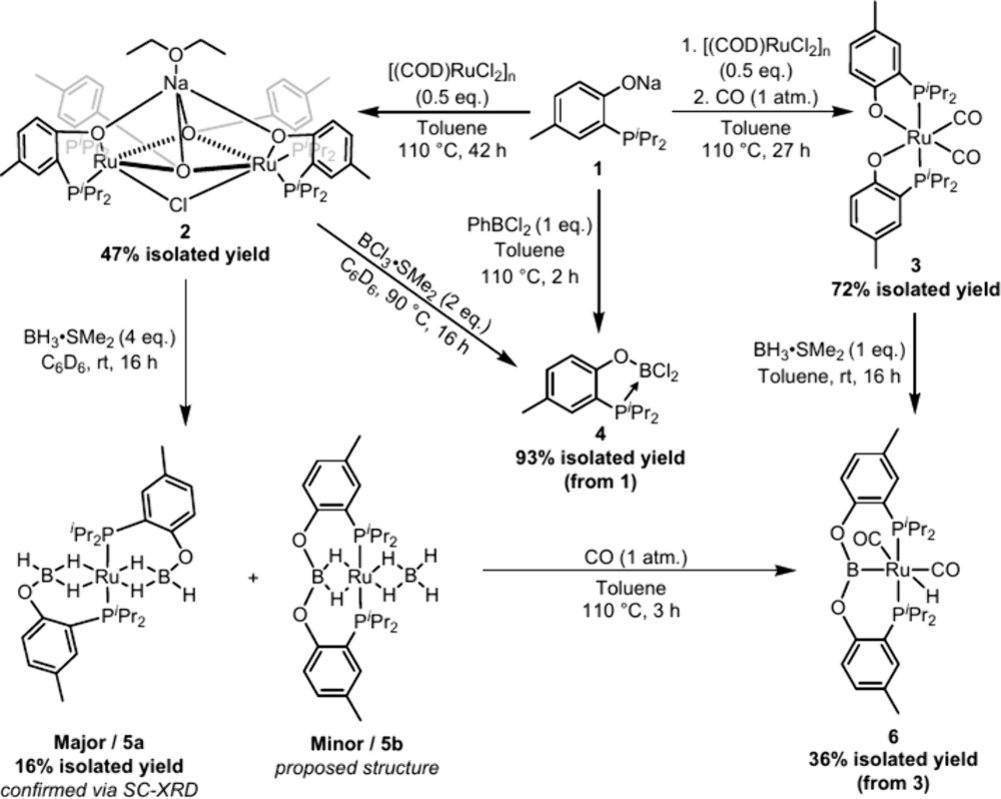
Synthesis of Ruthenium Complexes

**Figure 2:**
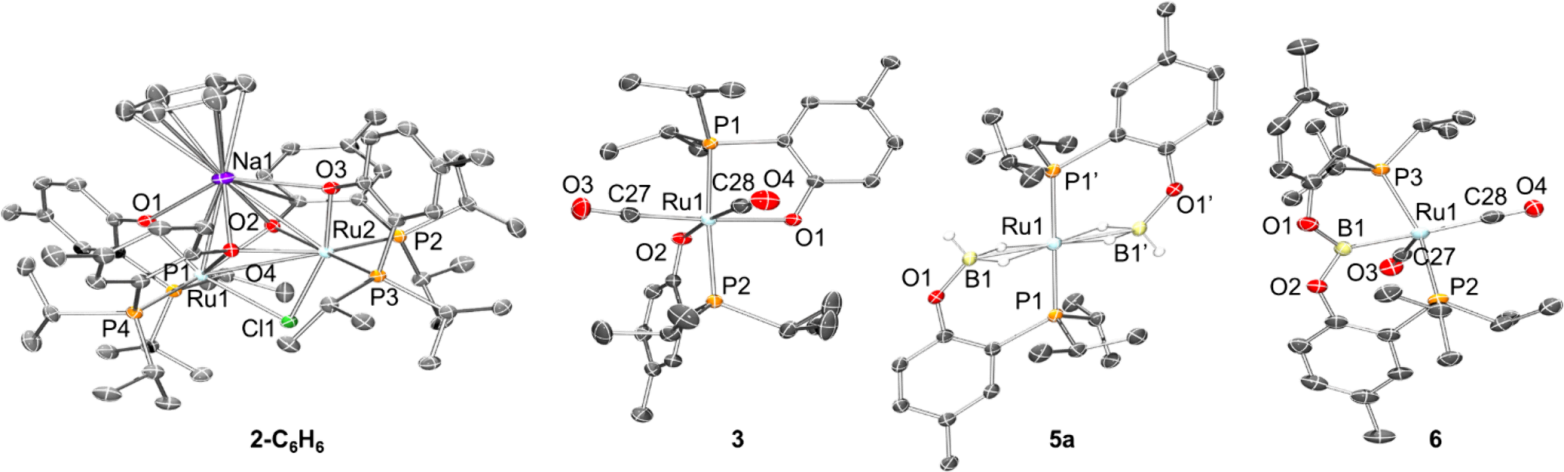
POV-Ray rendition^32^ of the ORTEP drawing^33^ (50% thermal ellipsoids) of **2-C_6_H_6_**, **3**, **5a** and **6** showing selected atom labeling. For **2-C_6_H_6_**, **3**, and **6**, hydrogen atoms are omitted for clarity. For **5a**, the hydrogen atoms are shown at an arbitrarily chosen small radius. Selected bond lengths (Å) and angles (deg) for **2**: Ru1–P1, 2.2428(8); Ru1–P4, 2.2540(8); Ru1–O1, 2.0694(18); Ru1–O2, 2.2135(18); Ru1– O4, 2.145(2); Ru1–Cl1, 2.4478(6); Ru2–P2, 2.2503(8); Ru2–P3, 2.2437(8); Ru2–O2, 2.1421(18); Ru2–O3, 2.0679(19); Ru2–O4, 2.2197(18); Ru2–Cl1, 2.4448(7); Na1–O1, 2.615(2); Na1–O2, 2.300(2); Na1–O3, 2.526(2); Na1–O4, 2.300(2); P1–Ru1–P4, 102.73(3); P1–Ru1–O4, 173.21(5); P4–Ru1–O2, 153.42(6); O1–Ru1–Cl1, 163.06(5); P2–Ru2–P3, 102.46(3); P2–Ru2–O4, 153.75(5); P3–Ru2–O2, 172.86(6); O3–Ru2–Cl1, 162.93(5); Ru1–O4–Ru2, 91.99(8); Ru1–O2–Ru2, 92.23­(7); Ru1–Cl1–Ru2, 79.840(18). Selected bond lengths (Å) and angles (deg) for **3**: Ru1–P1, 2.3760(4); Ru1–P2, 2.3711(4); Ru1–O1, 2.0903(11); Ru1–O2, 2.0857(12); Ru1–C27, 1.8673(18); Ru1– C28, 1.8790(18); P1–Ru1–P2, 167.378(17); O1–Ru1–C27, 175.58(6); O2–Ru1–C28, 173.62(6). Selected bond lengths (Å) and angles (deg) for **5a**: Ru1–P1, 2.3452(4); Ru1–B1, 2.1600(19); B1–O1, 1.408(3); P1–Ru1–B1, 88.99(6). Selected bond lengths (Å) and angles (deg) for **6**: Ru1–P3, 2.3258(8); Ru1–P2, 2.3310(9); Ru1–B1, 2.137(4); Ru1–C27, 1.935(4); Ru1–C28, 1.967(4); B1–O1, 1.397(4); B1–O2, 1.370(4); P3–Ru1–P2, 161.25(4); B1–Ru1–C28, 169.11(15).

Compounds **2** and **3** were then explored in reactions with boron reagents as “linchpins” for the formation of POBOP pincer complexes. However, treatment of **2** with 1 equiv of BCl_3_·SMe_2_ per Ru at ambient temperature for 16 h resulted in an intractable mixture of products, whereas thermolysis of an analogous mixture led to loss of the PO fragment from the Ru center and generation of **4** as the main P-containing product (the fate of Ru was not established). Interestingly, the ^31^P NMR resonance of **4** displays discernible coupling to both ^11^B (1:1:1:1 quartet, *J*_P–11B_ = 132 Hz) and ^10^B isotopes (1:1:1:1:1:1:1 septet, *J*_P–10B_ ≈ 45 Hz). Treatment of **2** with BH_3_·SMe_2_ (1 equiv per Ru) in C_6_D_6_ at room temperature for 16 h resulted in ca. 50% consumption of the starting material to two spectroscopically similar products **5a** and **5b**. Addition of at least 2 equiv of BH_3_·SMe_2_ per Ru resulted in complete consumption of **2** and formation of **5a** and **5b** in a 5:1 ratio in C_6_D_6_. This ratio was closer to 1:1 when the reaction was performed in chloroform, but appeared to be stable to changes of solvent after the fact. Thus, we cannot be sure whether the slight preference for **5a** is of kinetic or thermodynamic nature. Compound **5a** could be isolated from the mixture in modest yields in ∼ 94% purity (NMR evidence) by washing the solid mixture with pentane.

Compounds **5a** and **5b** give rise to very similar patterns and chemical shifts in the ^1^H NMR spectra, and with only a slight difference (ca. 0.1 ppm) in their ^31^P NMR chemical shifts. However, they differ in the pattern of the hydridic ^1^H NMR resonances: a single hydride resonance (δ −12.30 ppm) of intensity 4H for **5a**, but two distinct hydride resonances (δ −9.49 ppm, −15.11 ppm) of intensity 2H each for **5b**. Both compounds also display resonances that are quite broad and of intensity 2H (6.78 and 5.74 ppm for **5a** and **5b**, respectively), indicative of terminal B–H bonds.^34^ The single-crystal XRD study of **5a** (Figure 2) demonstrated that it contains two phosphine/(aryloxytrihydridoborate) ligands with a total of four bridging H’s filling out an octahedral geometry about Ru.

Complexes containing hydridoborates as part of a bidentate ligand have been reported for Ru.^35–37^ We surmised that **5b** is an isomer of **5a** analogous to **B** (Figure 1).^17^ The PBP-derived dihydroborate moiety seen in **B** has been frequently observed in various PBP complexes of other metals, as well.^20,21,23,38^ Compound **B** shares the 2H:2H:2H pattern seen in **5b** arising from three types of boron-bound hydrogens with analogous chemical shifts (∼6 ppm, −5.78, and −14.57 ppm). In support of this notion, we carried out a DFT computational study (B3LYP-D3 ^39–43^ /LANL2DZ/6-31G­(d) via Gaussian 16 ^44,45^ ) of **5a** and **5b**, establishing these two structures as minima of similar free energy (**5a** is lower than **5b** by 3.1 kcal/mol). The calculated structure of **5a** matches the XRD metrics well (calculated Ru–B distance of 2.193 vs 2.1600(19) Å). Analysis of the WBI values (Figure 3) shows that hydrides bridging B and Ru interact increasingly more strongly with Ru (and less strongly with B) upon replacement of the nonbridging hydrides with one or two oxygens.

**Figure 3:**
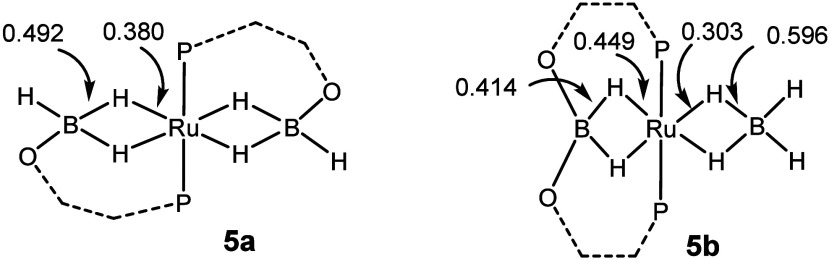
Wiberg bond index (WBI) values for the B–H and Ru–H bonds in **5a** and **5b**.

The reaction of **3** with BH_3_·SMe_2_ (1 equiv per Ru) was simpler, and yielded the pincer complex (POBOP)­Ru­(CO)_2_H (**6**) quantitatively (in situ NMR evidence), even though we were only able to isolate it in 36% yield (as an off-white powder) upon workup. Additionally, thermolysis (100 °C) of a mixture of **5a** and **5b** under an atmosphere of CO also cleanly forms **6**. This transformation ostensibly releases an equivalent of BH_3_ but proceeds equally well with or without an additional “capture agent” such as DABCO or SMe_2_.^46^ Compound **6** displays a ^11^B NMR resonance (54.0 ppm) that is consistent with an sp^2^-hybridized boryl-metal interaction, appearing in a range similar to Ru–Bcat complexes that also contain two oxygenous substituents on B.^47^ The ^1^H NMR chemical shift (δ −8.19 ppm) observed for Ru–H is typical for a hydride *trans* to a metal carbonyl,^48^ and the *cis* disposition of the CO was confirmed by the observation of two IR bands of approximately equal intensity. Their energies (ν_CO_ = 1998 and 1951 cm^–1^) are very similar to those in Caulton’s (^i^Pr_2_PMe)_2_Ru­(H)_2_(CO)_2_ (ν_CO_ = 1995 and 1952 cm^–1^),^49^ and also to those in (PBP)­Ru­(H)­(CO)_2_ (ν_CO_ = 1994 and 1967 cm^–1^, where the PBP ligand is the boryl/bis­(phosphine pincer of compound **B**).^50^ The XRD study (Figure 2) confirmed the proposed connectivity. The Ru-bound boron is in an essentially perfect planar environment and the Ru–B distance of 2.137(4) Å is in the range for the known Ru–B­(oryl) bonds.^38^ The P–Ru–P angle in **5** (161.25(4)°) is considerably smaller than the P–Ir–P angle in **E** (174.12(3)°),^20^ suggesting substantial flexibility of the (POBOP)­M framework. Addition of pyridine or of 4-dimethylaminopyridine to a C_6_D_6_ solution of **6** did not result in a perceptible change in the ^11^B or the ^31^P NMR chemical shift of the resonances of **6**, indicating that the boron site of it is a weak Lewis acid.

In summary, we have successfully applied the metal-templated “reverse logic” approach to the synthesis of boryl-centered POBOP pincer complexes of Ru. However, it appears that success in the preparation of the boryl-metal functionality hinges on the proper reagent choice and stoichiometry that leads to the formation of a stable, 18-electron product.

## Supplementary Material





## Notes

Details of synthetic procedures and X-ray diffractometry, graphical NMR spectra and other spectroscopic characterization, and computational details (PDF)
Coordinates for the computed structures of **5a** and **5b** (XYZ)
