# Supplementary material for: Flash Communication: A Metal-First Approach to Ruthenium Complexes of a Boryl-Centered POBOP Pincer Ligand
Source: Organometallics. 2026 Apr 24;45(9):1017–21. doi: 10.1021/acs.organomet.6c00078 (PMC13169357; doi:10.1021/acs.organomet.6c00078)
Supplement: Supplementary file 1 [file om6c00078_si_001.pdf]

Supporting Information

Flash Communication: A Metal-First Approach to Ruthenium Complexes of a Boryl-Centered POBOP Pincer Ligand

Jovanny J. Contreras[a], Mason W. Bayles[a], Nattamai Bhuvanesh[a], Lori A. Watson,* [b] and Oleg V. Ozerov*[a]

[a]*Department of Chemistry, Texas A&M University, College Station, Texas 77842*

[b]*Department of Chemistry, Earlham College, 801 National Rd W, Richmond, IN 47374*

ozerov@chem.tamu.edu

watsolo@earlham.edu

Table of Contents

I. General considerations 3
II. Synthesis of compounds 4
III. X-ray structural determination details 13
IV. NMR and IR spectra 19
V. Computational Details 68
VI. References 69

I. General considerations

All manipulations were performed under an Ar atmosphere using standard Schlenk line or glovebox techniques. Pentane, tetrahydrofuran, diethyl ether, toluene, and isooctane were dried and deoxygenated (by purging) using a solvent purification system (Innovative Technology Pure Solv MD-5 Solvent Purification System) and stored over molecular sieves in an Ar-filled glove box. C_6D_6, $CDCl_3$, CD_2Cl_2, were dried over calcium hydride, vacuum transferred and stored over molecular sieves in an Ar-filled glovebox. Celite and silica were dried at 250 and 200 °C, respectively, under vacuum overnight and stored in an Ar-filled glovebox. $[(COD)RuCl_2]_n$ and sodium 2-diisopropylphosphino-4-methylphenolate (**1**) were prepared according to published procedures.[1,2] All other chemicals were used as received from commercial vendors. NMR spectra were recorded on a Varian VnmrS 500 (^{1}H NMR, 500 MHz; ^{13}C{^{1}H} NMR, 126 MHz; ^{31}P{^{1}H} NMR, 202 MHz; ^{27}Al{^{1}H} NMR, 130 MHz), Varian Inova 500 (^{1}H NMR, 500 MHz; ^{13}C{^{1}H} NMR, 126 MHz; ^{31}P{^{1}H} NMR, 202 MHz), Bruker Avance NEO 400 (^{1}H NMR, 400 MHz; ^{13}C{^{1}H} NMR, 101 MHz; ^{31}P{^{1}H} NMR, 162 MHz), and Bruker Avance NEO 500 (^{1}H NMR, 500 MHz; ^{13}C{^{1}H} NMR, 126 MHz; ^{31}P{^{1}H} NMR, 202 MHz) spectrometers. Chemical shifts are reported in δ (ppm). For ^{1}H and ^{13}C NMR spectra, the residual solvent peak was used as an internal reference (^{1}H NMR: δ 7.16 for C_6D_6, 7.26 ppm for $CDCl_3$, and 5.32 for CD_2Cl_2; ^{13}C NMR: δ 128.06 for C_6D_6 and δ 77.16 ppm for $CDCl_3$). ^{31}P NMR spectra were referenced externally with neat phosphoric acid at δ 0 ppm. ^{11}B NMR spectra were referenced externally with neat $BF_3·OEt_2$ at δ 0 ppm. Elemental analyses were performed by Robertson Microlit Laboratories (Ledgewood, NJ).

II. Synthesis of compounds

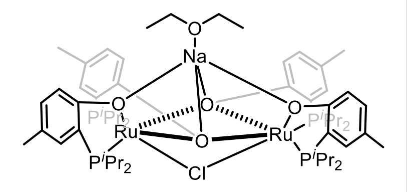

Synthesis of $NaRu_2(PO)_2(P\mu\text{-}O)_2(\mu\text{-}Cl)\bullet Et_2O$ (2). In a 100 mL PTFE stoppered Schlenk flask, **1** (600 mg, 2.44 mmol) and dichloro(1,5-cyclooctadiene)ruthenium(II) polymer (341 mg, 1.22 mmol) were combined and dissolved in approximately 15 mL of toluene. The solution was then removed from the glovebox and stirred in a 110 °C oil bath. After approximately 42 h, the solution was cooled to room temperature, transferred back to the glovebox, and filtered through a pad of celite. The filtrate was concentrated under reduced pressure to reveal a dark brown residue which was then dissolved in ca. 6 mL of Et_2O and left to recrystallize for at -37 °C. The dark brown mother liquor was removed to reveal a red solid that was further was washed with cold pentane (3 x 2 mL) and dried to afford the product as a red solid (348 mg, 0.283 mmol, 47%). The product crystallizes with a single equivalent of Et_2O that can be observed via NMR spectroscopy and persists after the removal of volatiles. ^{1}H NMR (400 MHz, C_6D_6): δ 7.78 (d, $J_{H–P}$ =7 Hz, 2H, Ar–*H*), 7.20 (d, $J_{H–P}$ =7 Hz, 2H, Ar–*H*), 7.02 (m, 2H, Ar–*H*), 6.81–6.75 (m, 4H, Ar–*H*), 6.52 (d, $J_{H–H}$ = 8 Hz, 2H, Ar–*H*), 3.19 (q, $J_{H–H}$ = 7 Hz, 4H, Et_2O-C*H*$_2$), 2.87 (dsept, $J_{H–P}$ = 8 Hz, $J_{H–H}$ = 7 Hz, 2H, C*H*(Me)$_2$), 2.54 (dsept, $J_{H–P}$ = 7 Hz, $J_{H–H}$ = 7 Hz, 2H, C*H*(Me)$_2$), 2.41–2.29 (m, 10H, C*H*(Me)$_2$ and Ar–*Me*), 2.15 (s, 6H, Ar–*Me*), 1.69 (dd, $J_{H–P}$ = 10 Hz, $J_{H–H}$ = 7 Hz, 6H, CH(*Me*)$_2$), 1.62 (dd, $J_{H–P}$ = 10 Hz, $J_{H–H}$ = 7 Hz, 6H, CH(*Me*)$_2$), 1.48 (dd, $J_{H–P}$ = 15 Hz, $J_{H–H}$ = 8 Hz, 6H, CH(*Me*)$_2$), 1.42–1.29 (m, 18H, CH(*Me*)$_2$), 1.03 (t, $J_{H–H}$ = 7 Hz, 6H, Et_2O-C*H*$_3$), 0.75 (dd, $J_{H–P}$ = 14 Hz, $J_{H–H}$ = 7 Hz, 6H, CH(*Me*)$_2$), 0.67 (dd, $J_{H–P}$ = 15 Hz, $J_{H–H}$ = 7 Hz, 6H, CH(*Me*)$_2$). ^{1}H{^{31}P} NMR (500 MHz, C_6D_6): δ 7.78 (s, 2H, Ar–*H*), 7.20 (s, 2H, Ar–*H*), 7.02 (m, 2H, Ar–*H*), 6.78 (t, $J_{H–H}$ = 8 Hz, 4H, Ar–*H*), 6.52 (d, $J_{H–H}$ = 8 Hz, 2H, Ar–*H*), 3.19 (q, $J_{H–H}$ = 7 Hz, 4H, Et_2O-C*H*$_2$), 2.87 (sept, $J_{H–H}$ = 7 Hz, 2H, C*H*(Me)$_2$), 2.54 (sept, $J_{H–H}$ = 7 Hz, 2H, C*H*(Me)$_2$), 2.41–2.29 (m, 10H, C*H*(Me)$_2$

and Ar–*Me*), 2.15 (s, 6H, Ar–*Me*), 1.69 (d, $J_{H–H}$ = 7 Hz, 6H, CH(*Me*)$_2$), 1.62 (d, $J_{H–H}$ = 7 Hz, 6H, CH(*Me*)$_2$), 1.48 (d, $J_{H–H}$ = 8 Hz, 6H, CH(*Me*)$_2$), 1.40–1.36 (m, 12H, CH(*Me*)$_2$), 1.33 (d, $J_{H–H}$ = 7 Hz, 6H, CH(*Me*)$_2$), 1.03 (t, $J_{H–H}$ = 7 Hz, 6H, Et$_2$O-C*H*$_3$), 0.75 (d, $J_{H–H}$ = 7 Hz, 6H, CH(*Me*)$_2$), 0.67 (d, $J_{H–H}$ = 7 Hz, 6H, CH(*Me*)$_2$). ^{13}C{^{1}H} NMR (101 MHz, C_6D_6): δ 178.2 (d, $J_{C–P}$ = 17 Hz, C_{Ar}) 172.7 (d, $J_{C–P}$ = 12 Hz, C_{Ar}), 133.5 (s, C_{Ar}), 131.3 (d, $J_{C–P}$ = 15 Hz, C_{Ar}), 130.1 (s, C_{Ar}), 127.5 (s, C_{Ar}), 127.1 (s, C_{Ar}), 126.7 (s, C_{Ar}), 125.8 (d, $J_{C–P}$ = 3 Hz, C_{Ar}), 121.7 (d, $J_{C–P}$ = 5 Hz, C_{Ar}), 121.5 (br d, $J_{C–P}$ = 8 Hz, C_{Ar}), 118.9 (d, $J_{C–P}$ = 9 Hz, C_{Ar}), 66.1 (s, Et$_2$O-*C*H$_2$), 38.0 (d, $J_{C–P}$ = 24 Hz, *C*H(Me)$_2$), 30.6 (d, $J_{C–P}$ = 18 Hz, *C*H(Me)$_2$), 30.3 (d, $J_{C–P}$ = 26 Hz, *C*H(Me)$_2$), 29.5 (d, $J_{C–P}$ = 19 Hz, *C*H(Me)$_2$), 23.1 (d, $J_{C–P}$ = 5 Hz, CH(*Me*)$_2$), 21.4 (m, two overlapping CH(*Me*)$_2$), 21.1–20.9 (m, two CH(*Me*)$_2$ and Ar–*Me* overlapping), 20.8 (s, Ar–*Me*), 20.2 (d, $J_{C–P}$ = 6 Hz, CH(*Me*)$_2$), 19.2 (d, $J_{C–P}$ = 4 Hz, CH(*Me*)$_2$), 18.8 (s, CH(*Me*)$_2$), 15.4 (s, Et$_2$O-*C*H$_3$). ^{31}P{^{1}H} NMR (162 MHz, C_6D_6): δ 74.0 (d, $J_{P–P}$ = 35 Hz), 69.6 (d, $J_{P–P}$ = 35 Hz). $C_{56}H_{90}ClNaO_5P_4Ru_2$: C, 54.78; H, 7.39; Found: C, 54.61; H, 7.37.

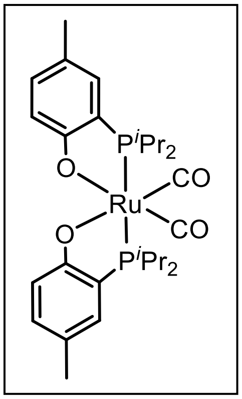

Synthesis of Ru(PO)$_2$(CO)$_2$ (3). In a 25 mL PTFE stoppered Schlenk flask, **1** (402 mg, 1.63 mmol) and dichloro(1,5-cyclooctadiene)ruthenium(II) polymer (227 mg, 0.810 mmol) were combined and dissolved in approximately 3 mL of toluene. The flask was then removed from the glovebox and stirred in a 110 °C oil bath. After 24 h the solution was cooled to room temperature, degassed via freeze-pump-thaw (3 x 5 min), and refilled with carbon monoxide (1 atm). The solution was placed back in a 110 °C oil bath and stirred for an additional 3 h. After cooling to room temperature, the flask was returned to the glovebox and filtered through a pad of celite. The filtrate was concentrated under reduced pressure and triturated with isooctane (5 x 3 mL) to reveal a brown solid. The resultant solid was collected on a frit and washed with pentane (3 x 3

mL) to afford the product as an off-white solid (352 mg, 0.583 mmol, 72%). ^{1}H NMR (400 MHz, C_6D_6): δ 6.95–6.88 (m, 4H, Ar–*H*), 6.77 (dt, J_{H-H} = 8 Hz, J_{H-P} = 2 Hz, 2H, Ar–*H*), 2.77–2.68 (m, 2H, C*H*(Me)$_2$), 2.39–2.31 (m, 2H, C*H*(Me)$_2$), 2.23 (s, 6H, Ar–*Me*), 1.47 (dvt, $J_{H-H} \approx J_{H-P}$ = 7 Hz, 6H, CH(*Me*)$_2$), 1.41 (dvt, $J_{H-H} \approx J_{H-P}$ = 7 Hz, 6H, CH(*Me*)$_2$), 1.25 (dvt, $J_{H-H} \approx J_{H-P}$ = 7 Hz, 6H, CH(*Me*)$_2$), 1.04 (dvt, $J_{H-H} \approx J_{H-P}$ = 7 Hz, 6H, CH(*Me*)$_2$). ^{1}H{^{31}P} NMR (500 MHz, C_6D_6): δ 6.94 (dd, J_{H-H} = 8 Hz, J_{H-H} = 2 Hz, 2H, Ar–*H*), 6.91 (s, 2H, Ar–*H*), 6.77 (d, J_{H-H} = 8 Hz, 2H, Ar–*H*), 2.73 (sept, J_{H-H} = 7 Hz, 2H, C*H*(Me)$_2$), 2.33 (sept, J_{H-H} = 7 Hz, 2H, C*H*(Me)$_2$), 2.23 (s, 6H, Ar–*Me*), 1.47 (d, J_{H-H} = 7 Hz, 6H, CH(*Me*)$_2$), 1.41 (d, J_{H-H} = 7 Hz, 6H, CH(*Me*)$_2$), 1.25 (d, J_{H-H} = 7 Hz, 6H, CH(*Me*)$_2$), 1.04 (d, J_{H-H} = 7 Hz, 6H, CH(*Me*)$_2$). ^{13}C{^{1}H} NMR (101 MHz, C_6D_6): δ 199.4 (t, J_{C-P} = 10 Hz, *C*O), 176.8 (t, J_{C-P} = 13 Hz, *C*O), 134.2 (s, C_{Ar}), 130.5 (s, C_{Ar}), 128.6 (s, C_{Ar}), 122.9 (vt, J_{C-P} = 4 Hz, C_{Ar}), 120.7 (vt, J_{C-P} = 5 Hz, C_{Ar}), 113.5 (vt, J_{C-P} = 22 Hz, C_{Ar}), 27.8 (vt, J_{C-P} = 13 Hz, *C*H(Me)$_2$), 27.0 (vt, J_{C-P} = 14 Hz, *C*H(Me)$_2$), 21.5 (s, CH(*Me*)$_2$), 20.6 (vt, J_{C-P} = 3 Hz, CH(*Me*)$_2$), 20.5 (s, Ar–*Me*), 20.0 (s, CH(*Me*)$_2$), 19.3 (s, CH(*Me*)$_2$). ^{31}P{^{1}H} NMR (162 MHz, C_6D_6): δ 69.8 (s). ν_{CO} = 2024, 1957 cm^{-1}. Anal. Calc. for $C_{28}H_{40}O_4P_2Ru$: C, 55.71; H, 6.68; Found: C, 55.73; H, 6.80.

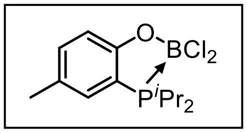

Independent synthesis of (PO)BCl$_2$ (BOP-Cl$_2$) (4). In a 25 mL PTFE stoppered Schlenk flask, BCl_3•SMe_2 (112 mg, 6.25 mmol) and **1** (155 mg, 6.29 mmol) were added and suspended in 6 mL of toluene. The flask was then removed from the glovebox and placed in a 110 °C oil bath. After 2 h, the flask was transferrred back into the glovebox, and the mixture was filtered through a pad of celite into a 25 mL Schlenk flask. The volatlies were removed under reduced pressure, revealing the product as a white solid (178 mg, 5.84 mmol, 93%) that was used without further purification and determined to be >95% by ^{31}P NMR spectroscopy. ^{1}H NMR (400 MHz, C_6D_6): δ 6.89 (dd, J_{H-H} = 8 Hz, J_{H-P} = 4 Hz, 1H, Ar–*H*),

6.81 (br d, $J_{H–H}$ = 8 Hz, 1H, Ar–*H*), 6.77 (d, $J_{H–P}$ = 7 Hz, 1H, Ar–*H*), 2.12 (m, 2H, C*H*(Me)$_2$), 1.98 (s, 3H, Ar–*Me*), 1.00 (dd, $J_{H–P}$ = 17 Hz, $J_{H–H}$ = 7 Hz, 12H, CH(*Me*)$_2$), 0.92 (dd, $J_{H–P}$ = 16 Hz, $J_{H–H}$ = 7 Hz, 12H, CH(*Me*)$_2$). ^{1}H{^{31}P} NMR (500 MHz, C_6D_6): δ 6.89 (d, $J_{H–H}$ = 8 Hz, 1H, Ar–*H*), 6.81 (d, $J_{H–H}$ = 8 Hz, 1H, Ar–*H*), 6.77 (s, 1H, Ar–*H*), 2.12 (sept, $J_{H–H}$ = 7 Hz, 2H, C*H*(Me)$_2$), 1.98 (s, 3H, Ar–*Me*), 1.00 (dd, $J_{H–H}$ = 7 Hz, 12H, CH(*Me*)$_2$), 0.92 (dd, $J_{H–H}$ = 7 Hz, 12H, CH(*Me*)$_2$). ^{13}C{^{1}H} NMR (101 MHz, C_6D_6): δ 164.7 (d, $J_{C–P}$ = 20 Hz, C_{Ar}), 136.8 (d, $J_{C–P}$ = 2 Hz, C_{Ar}), 130.7 (s, C_{Ar}), 130.2 (d, $J_{C–P}$ = 7 Hz, C_{Ar}), 117.1 (s, C_{Ar}), 108.5 (br d, $J_{C–P}$ = 63 Hz, C_{Ar}), 21.4 (d, $J_{C–P}$ = 33 Hz, *C*H(Me)$_2$), 20.2 (s, Ar–*Me*), 17.4 (d, $J_{C–P}$ = 3 Hz, CH(*Me*)$_2$), 17.0 (s, CH(*Me*)$_2$). ^{31}P{^{1}H} NMR (162 MHz, C_6D_6): δ 6.52 (q, $J_{P–B}$ = 132 Hz). ^{11}B{^{1}H} NMR (128 MHz, C_6D_6): δ 7.47 (d, $J_{B–P}$ = 132 Hz).

Reaction of 2 with BCl_3•SMe_2 at room temperature. In a J. Young tube, **2** (9.3 mg, 0.0076 mmol) and BCl_3•SMe_2 (3.0 mg, 0.016 mmol) were added and dissolved in approximately 400 µL of C_6D_6. The tube was left to react for approximately 16 h, then the contents of the tube were analyzed by ^{31}P NMR spectroscopy to reveal a large mixture of unidentified products (Figures S16 & S17).

Reaction of 2 with BCl_3•SMe_2 at elevated temperature. In a J. Young tube, **2** (12.1 mg, 0.0105 mmol) and BCl_3•SMe_2 (4.1 mg, 0.023 mmol) were added and dissolved in approximately 400 µL of C_6D_6. The tube was then removed from the glovebox and placed in a 90 °C oil bath. After heating for approximately 16 h, the contents of the tube were analyzed by ^{31}P NMR spectroscopy to reveal **$BOPCl_2$** as the major product (Figures S18 & S19).

Reaction of 2 with BH_3•SMe_2, observation of two products in a 5:1 ratio. In a J. Young tube, **2** (18 mg, 0.015 mmol) and BH_3•SMe_2 (14 µL, 0.15 mmol) were added and dissolved in approximately 400 µL of C_6D_6. The tube was left to react for approximately 16 h, then the contents

of the tube were analyzed by multinuclear NMR spectroscopy, revealing the presence of two products in a 5:1 ratio. (Figure S20).

Reaction of 2 with BH_3•SMe_2, observation of two products in a 1:1 ratio. In a J. Young tube, **2** (9.1 mg, 0.0074 mmol) and BH_3•SMe_2 (7.0 μL, 0.074 mmol) were added and dissolved in approximately 400 μL of $CDCl_3$. The tube was left to react for approximately 16 h, then the contents of the tube were analyzed by multinuclear NMR spectroscopy, revealing the presence of two products in a 1:1 ratio. (Figure S21).

Reaction of 2 with differing equivalents of BH_3•SMe_2. In three separate J. Young tubes, **2** ((**A:** 11.6 mg, 0.00943 mmol), **(B:** 12.3 mg, 0.0100 mmol), **(C:** 9.3 mg, 0.0076 mmol)) and BH_3•SMe_2 ((**A:** 2 μL, 0.02 mmol), (**B:** 3 μL, 0.03 mmol), (**C:** 3 μL, 0.03 mmol)) were added and dissolved in approximately 400 μL of C_6D_6. After letting the contents of the tubes react overnight, they were analyzed by ^{31}P NMR spectroscopy, revealing different ratios of starting material to two new products (approx. **A:** 50%, **B:** 75%, and **C:** 100% starting material consumption, respectively). (Figure S23.)

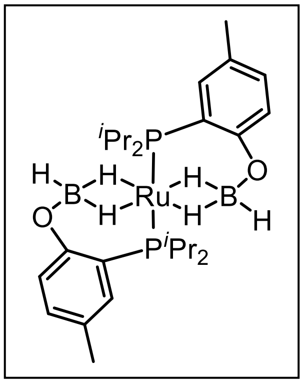

Reaction of 2 with BH_3•SMe_2; isolation of $(H_3BOP)_2Ru$ (5a). In a 20 mL scintillation vial, **2** (61.2 mg, 0.498 mmol) was dissolved in approximately 5 mL of toluene. To this red solution, BH_3•SMe_2 (10 μL, 8.0 mg, 0.25 mmol) was added, and the solution was left to stir overnight. The volatiles were removed under vacuum, and the residue was washed with pentane (3 × 1 mL) to give a beige solid as the product (15 mg, 0.26 mmol, 26%). ^{1}H NMR (500 MHz, C_6D_6): δ 7.39 (m, 2H, Ar–*H*), 7.22 (dt, d, $J_{H–H}$ = 8 Hz, $J_{H–P}$ = 2 Hz, 2H, Ar–*H*), 6.94 (d, $J_{H–H}$ = 8 Hz, 2H, Ar–*H*), 6.78 (br s, 2H, RuH_2BH_2), 2.53 (m, 4H, C*H*$(Me)_2$), 2.10 (s, 6H, Ar–*Me*), 1.17 (dvt, $J_{H–H}$ ≈ $J_{H–P}$ = 7 Hz, 12H, CH(*Me*)$_2$), 1.07 (dvt, $J_{H–H}$ ≈

$J_{H–P}$ = 7 Hz, 12H, CH(Me)$_2$), -12.30 (br s, 4H, Ru–H). $^{31}P\{^1H\}$ NMR (162 MHz, C_6D_6): δ 33.1 (s). $^{11}B\{^1H\}$ NMR (128 MHz, C_6D_6): δ 39.3 (br s).

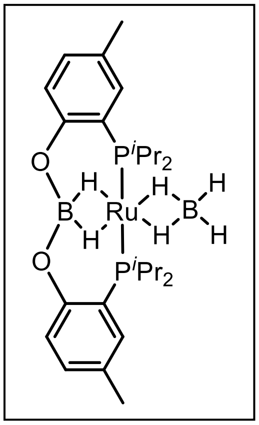

Spectroscopic data for (POB(H$_2$)OP)Ru(BH$_4$) (5b): ^1H NMR (500 MHz, C_6D_6): δ 7.35 (m, 2H, Ar–H), 7.21 (m, 2H, Ar–H, **overlap with 5a**), 6.88 (d, $J_{H–H}$ = 8 Hz, 2H, Ar–H), 5.74 (br s, 2H, RuH$_2$BH_2), 2.72 (m, 4H, CH(Me)$_2$), 2.09 (s, 6H, Ar–Me), 1.17 (dvt, $J_{H–H} \approx J_{H–P}$ = 7 Hz, 12H, CH(Me)$_2$, **overlap with 5a**), 1.07 (dvt, $J_{H–H} \approx J_{H–P}$ = 7 Hz, 12H, CH(Me)$_2$, **overlap with 5a**), –9.49 (br s, 2H, Ru–H), -15.11 (br s, 2H, Ru–H). $^{31}P\{^1H\}$ NMR (162 MHz, C_6D_6): δ 33.2 (s). $^{11}B\{^1H\}$ NMR (128 MHz, C_6D_6): δ 36.7 (br s, O_2BH_2), 23.7 (br s, BH_4).

Initial observation of the clean formation of 6 from 3. In a J. Young tube, **3** (18 mg, 0.030 mmol) was added and dissolved in approximately 400 µL of C_6D_6. To the colorless solution, BH_3•SMe_2 (3 µL, 0.03 mmol) was added, and the solution was left to sit at room temperature for 16 h. After 16 h, the contents of the tube were analyzed by multinuclear NMR spectroscopy to reveal near quantitative conversion to **6**, along with free SMe_2 and a small amount of unreacted BH_3•SMe_2 (Figure S30).

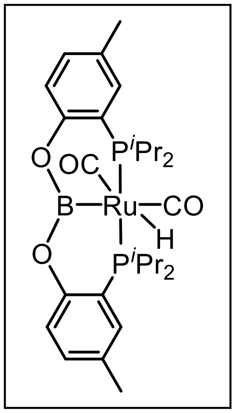

Synthesis of (POBOP)Ru(CO)$_2$H (6). In a 25 mL Schlenk flask, **3** (71 mg, 0.012 mmol) was added and dissolved in approximately 3 mL of toluene. To the solution, BH_3•SMe_2 (12 µL, 0.013 mmol) was added, and the solution was left to stir overnight (ca. 16 h). The next morning, an aliquot was taken to verify conversion to the desired product. The solution was then filtered through a pad of Celite and silica, and the filtrate was dried under reduced pressure to give an off-white residue. To the residue, pentane was added and removed under vacuum (3 × 1 mL) to give a white powder. The powder was then collected on a frit and washed

with pentane (3 x 1 mL) to afford the product a white solid (26 mg, 0.042 mmol, 36%). ^{1}H NMR (400 MHz, C_6D_6): δ 7.23 (dt, $J_{H–H}$ = 8 Hz, $J_{H–P}$ = 2 Hz, 2H, Ar–*H*), 7.00–6.97 (m, 2H, Ar–*H*), 6.92 (dd, $J_{H–H}$ = 8 Hz, $J_{H–P}$ = 2 Hz, 2H, Ar–*H*), 2.23–2.06 (m, 10H, Ar–*Me* and C*H*(Me)$_2$), 1.19–0.98 (m, 24H, CH(*Me*)$_2$), –8.19 (t, $J_{H–P}$ = 22 Hz, 1H, Ru–*H*). ^{1}H{^{31}P} NMR (500 MHz, C_6D_6): δ 7.23 (d, $J_{H–H}$ = 8 Hz, 2H, Ar–*H*), 7.00–6.97 (m, 2H, Ar–*H*), 6.92 (dd, $J_{H–H}$ = 8 Hz, 2H, Ar–*H*), 2.23–2.06 (m, 10H, Ar–*Me* and C*H*(Me)$_2$), 1.14 (d, $J_{H–H}$ = 7 Hz, 6H, CH(*Me*)$_2$), 1.11 (d, $J_{H–H}$ = 7 Hz, 6H, CH(*Me*)$_2$), 1.06 (d, $J_{H–H}$ = 7 Hz, 6H, CH(*Me*)$_2$), 1.02 (d, $J_{H–H}$ = 7 Hz, 6H, CH(*Me*)$_2$), -8.19 (s, 1H, Ru–*H*). ^{13}C{^{1}H} NMR (101 MHz, C_6D_6): δ 206.9 (t, $J_{C–P}$ = 8 Hz, *C*O), 201.3 (t, $J_{C–P}$ = 9 Hz, *C*O), 159.5 (vt, $J_{C–P}$ = 5 Hz, C_{Ar}), 132.8 (s, C_{Ar}), 129.9 (vt, $J_{C–P}$ = 3 Hz, C_{Ar}), 129.8 (s, C_{Ar}), 122.1 (vt, $J_{C–P}$ = 2 Hz, C_{Ar}), 117.4 (vt, $J_{C–P}$ = 22 Hz, C_{Ar}), 26.8 (vt, $J_{C–P}$ = 13 Hz, *C*H(Me)$_2$), 26.4 (vt, $J_{C–P}$ = 15 Hz, *C*H(Me)$_2$), 20.9 (s, Ar–*Me*), 18.2 (vt, $J_{C–P}$ = 2 Hz, CH(*Me*)$_2$), 17.9 (s, CH(*Me*)$_2$), 17.7 (s, CH(*Me*)$_2$), 17.6 (vt, $J_{C–P}$ = 3 Hz, CH(*Me*)$_2$). ^{31}P{^{1}H} NMR (162 MHz, C_6D_6): δ 49.5 (s). ^{11}B{^{1}H} NMR (128 MHz, C_6D_6): δ 54.0 (br s). ν_{CO} = 1998, 1951 cm^{-1}. Anal. Calc. for $C_{28}H_{41}BO_4P_2Ru$: C, 54.64; H, 6.71; Found: C, 54.39; H, 6.81.

Reaction of a mixture of 5a and 5b with CO. In a J. Young tube2 (7.2 mg, 0.0059 mmol) and BH_3•SMe_2 (5.5 μL, 0.058 mmol) were added and dissolved in approximately 400 μL of THF-h_8. The tube was left to react for approximately 16 h, then the contents of the tube were analyzed by multinuclear NMR spectroscopy, revealing the presence of two products in a 5:1 ratio. The contents of the tube were then degassed via FPT (3 × 5 min) and refilled with CO, followed by ^{31}P{^{1}H} NMR spectroscopy analysis. The tube was then placed in a 100 °C oil bath for approximately 16 h then analyzed once more, revealing significant conversion to X. The tube was left to stand for an additional 7 days and reanalyzed, revealing complete conversion to **6**. (Figure S37).

Reaction of a mixture of 5a and 5b with CO and a BH_3 capture agent (DABCO). In a J. Young tube, **2** (8.7 mg, 0.0071 mmol) and BH_3•SMe_2 (10 μL, 0.11 mmol) were added and dissolved in approximately 400 μL of C_6H_6. The tube was left to react for approximately 16 h, then the volatiles were removed under reduced pressure. The contents of the tube were then redissolved in C_6D_6, and DABCO (2 mg, 0.018 mmol) was added. The solution was then degassed via FPT (3 × 5 min) and refilled with CO. The tube was then placed in a 100 °C oil bath for 2.5 h then analyzed once more, revealing complete conversion to **6**, along with the presence of DABCO•BH_3 (Figures S38 & S39)

Reaction of a mixture of 5a and 5b with CO and a BH_3 capture agent (SMe_2). In a J. Young tube, **2** (8.7 mg, 0.0071 mmol) and BH_3•SMe_2 (10 μL, 0.11 mmol) were added and dissolved in approximately 400 μL of C_6H_6. The tube was left to react for approximately 16 h, then the volatiles were removed under reduced pressure. The contents of the tube were then redissolved in C_6D_6, and SMe_2 (1.25 μL, 0.017 mmol) was added. The solution was then degassed via FPT (3 × 5 min) and refilled with CO. The tube was then placed in a 100 °C oil bath for 2.5 h then analyzed once more, revealing complete conversion to **6**, along with the presence of SMe_2•BH_3 (Figures S40 & S41).

Reaction of 6 with pyridine. In a J. Young tube, **6** (15.3 mg, 0.0249 mmol) was added and dissolved in approximately 400 μL of C_6D_6. To the solution, pyridine (2 μL, 0.0249 mmol) was added, and the contents of the tube were then analyzed by mutlinuclear NMR spectroscopy (**Figures S42, S43 & S44**).

Reaction of 6 with N,N-dimethylaminopyridine (DMAP). In a J. Young tube, **6** (15.1 mg, 0.0245 mmol) was added and dissolved in approximately 400 μL of C_6D_6. To the solution, DMAP

(3.0 mg, 0.025 mmol) was added, and the contents of the tube were then analyzed by mutlinuclear NMR spectroscopy (**Figures S45, S46 & S47**).

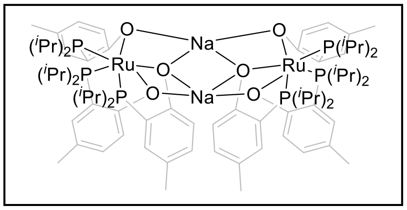

Synthesis of [NaRu(PO)$_3$]$_2$ (7). In a 25 mL PTFE stoppered Schlenk flask, **1** (342 mg, 1.39 mmol) and dichloro(1,5-cyclooctadiene)ruthenium(II) polymer (127 mg, 0.453 mmol) were combined and dissolved in approximately 3 mL of toluene. The solution was then removed from the glovebox and stirred in a 110 °C oil bath. After 18 h, the solution was cooled to room temperature, transferred back to the glovebox, and filtered through a pad of celite. The filtrate was then concentrated under reduced pressure to reveal a yellow residue which was triturated with isooctane (5 x 3 mL). The resultant yellow solid was then collected on a frit and washed with pentane (3 x 5 mL) to yield the product as a yellow solid (306 mg, 0.177 mmol, 78%). ^{1}H NMR (400 MHz, C_6D_6): δ 7.16–7.15 (m, 6H, Ar–*H*, resonance has slight overlap with residual solvent signal), 6.83–6.67 (m, 12H, Ar–*H*), 2.52 (m, 6H, C*H*(Me)$_2$), 2.34 (m, 6H, C*H*(Me)$_2$), 2.25 (s, 18H, Ar–*Me*), 1.38 (dvt, J_1 = 7 Hz, J_2 = 5 Hz, 18H, CH(*Me*)$_2$), 1.32 (dvt, J_1 = 7 Hz, J_2 = 4 Hz, 18H, CH(*Me*)$_2$), 1.14 (dvt, J_1 = 7 Hz, J_2 = 4 Hz, 18H, CH(*Me*)$_2$), 1.00 (dvt, $J_{H–H} \approx J_{H–P}$ = 7 Hz, 18H, CH(*Me*)$_2$). ^{13}C{^{1}H} NMR (101 MHz, C_6D_6): δ 173.1 (br s, C_{Ar}), 132.1 (s, C_{Ar}), 131.7 (s, C_{Ar}), 128.6 (s, C_{Ar}), 122.4 (s, C_{Ar}), 120.3 (br s, C_{Ar}), 31.2 (br s), 22.1 (s, CH(*Me*)$_2$), 21.5 (s, CH(*Me*)$_2$), 21.2 (s, CH(*Me*)$_2$), 20.9 (br s). ^{31}P{^{1}H} NMR (162 MHz, C_6D_6): δ 60.7 (s).

III. X-ray structural determination details

X-ray data collection, solution, and refinement for NaRu$_2$(PO)$_2$(Pμ-O)$_2$(μ-Cl)•C$_6$H$_6$ (2-C$_6$H$_6$) (CCDC 2491264) – A Leica M80 microscope was used to identify a suitable single red block-shaped crystal of suitable dimensions (0.08 × 0.04 × 0.03 mm^3) from a representative sample of crystals of the same habit. The crystal mounted on a nylon loop was then placed in a cold nitrogen stream (Oxford) maintained at T = 99.9(7) K. Crystal screening, unit cell determination, and data collection were carried out using a XtaLAB Synergy, Dualflex, HyPix diffractometer. The diffraction pattern was indexed and the total number of runs and images was based on the strategy calculation from the program CrysAlisPro system (CCD 43.142a 64-bit (release 17-10-2024)).[3] Data were measured using *w* scans with Cu K_a radiation. Data was collected to a maximum resolution of Q = 74.502° (0.80 Å). The unit cell was refined using CrysAlisPro 1.171.43.143a on 48448 reflections, 290 % of the observed reflections.[3] Integrated Intensity information for each reflection was obtained by reduction of data frames using CrysAlisPro 1.171.43.143a.[3] The final completeness is 99.90 % out to 74.502° in Q. A multi-scan absorption correction was performed using CrysAlisPro 1.171.43.143a.[3] Empirical absorption correction using spherical harmonics, implemented in SCALE3 ABSPACK scaling algorithm. The absorption coefficient *m* of this material is 5.317 mm^{-1} at this wavelength (l = 1.54184Å) and the minimum and maximum transmissions are 0.850 and 1.000. Systematic reflection conditions and statistical tests of the data suggested the space group *P*-1 (# 2) and was confirmed by ShelXT structure solution program using dual methods.[5,6] The structure was refined by full matrix least squares minimization on $\boldsymbol{F}^2$ using version 2019/1 of ShelXL 2019/1.[5,6] All non-hydrogen atoms were refined anisotropically. Hydrogen atom positions were calculated geometrically and refined using the riding model.

X-ray data collection, solution, and refinement for Ru(PO)$_2$(CO)$_2$ (3) (CCDC 2491266) – A Leica M80 microscope was used to identify a suitable single colorless plate-shaped crystal of suitable dimensions (0.20 × 0.12 × 0.08 mm^3) from a representative sample of crystals of the same habit. The crystal mounted on a nylon loop was then placed in a cold nitrogen stream (Oxford) maintained at T = 99.99(10) K. Crystal screening, unit cell determination, and data collection were carried out using a XtaLAB Synergy, Dualflex, HyPix diffractometer. The diffraction pattern was indexed and the total number of runs and images was based on the strategy calculation from the program CrysAlisPro system CrysAlisPro system (CCD 43.142a 64-bit (release 17-10-2024)).[3] Data were measured using *w* scans with Cu K_a radiation. Data was collected to a maximum resolution of Q = 74.494° (0.80 Å). The unit cell was refined using CrysAlisPro 1.171.43.143a on 31241 reflections, 71 % of the observed reflections.[3] Integrated Intensity information for each reflection was obtained by reduction of data frames using CrysAlisPro 1.171.43.143a.[3] The final completeness is 100.00 % out to 74.494° in Q. A gaussian absorption correction was performed using CrysAlisPro 1.171.43.143a.[3] Numerical absorption correction based on gaussian integration over a multifaceted crystal model Empirical absorption correction using spherical harmonics, implemented in SCALE3 ABSPACK scaling algorithm. The absorption coefficient *m* of this material is 4.883 mm^{-1} at this wavelength (l = 1.54184Å) and the minimum and maximum transmissions are 0.578 and 1.000. Systematic reflection conditions and statistical tests of the data suggested the space group $P2_1/n$ (# 14) and was confirmed by ShelXT 2018/2 structure solution program using dual methods.[5,6] The structure was refined by full matrix least squares minimization on $\boldsymbol{F^2}$ using version 2019/1 of ShelXL 2019/1.[5,6] All non-hydrogen atoms were refined anisotropically. Hydrogen atom positions were calculated geometrically and refined using the riding model.

X-ray data collection, solution, and refinement for $(H_3BOP)_2Ru$ (5a) (CCDC 2519454) – A Leica M80 microscope was used to identify a suitable single colorless plate-shaped crystal of suitable dimensions ($0.09 \times 0.06 \times 0.03$ mm^3) from a representative sample of crystals of the same habit. The crystal mounted on a MITIGEN holder and was then placed in a cold nitrogen stream (Oxford) maintained at T = 100.00(10) K. Crystal screening, unit cell determination, and data collection were carried out using a XtaLAB Synergy, Dualflex, HyPix diffractometer. The diffraction pattern was indexed and the total number of runs and images was based on the strategy calculation from the program CrysAlisPro system CrysAlisPro system (CCD 43.142a 64-bit (release 17-10-2024)).[3] Data were measured using *w* scans with Cu K_a radiation. Data was collected to a maximum resolution of Q = 80.135° (0.78 Å).The unit cell was refined using CrysAlisPro 1.171.43.143a on 20418 reflections, 69 % of the observed reflections.[3] Integrated Intensity information for each reflection was obtained by reduction of data frames using CrysAlisPro 1.171.43.143a.[3] The final completeness is 100.00 % out to 80.135° in Q. A gaussian absorption correction was performed using CrysAlisPro 1.171.43.143a.[1] Numerical absorption correction based on gaussian integration over a multifaceted crystal model Empirical absorption correction using spherical harmonics, implemented in SCALE3 ABSPACK scaling algorithm. The absorption coefficient *m* of this material is 5.643 mm^{-1} at this wavelength (l = 1.54184Å) and the minimum and maximum transmissions are 0.113 and 0.505. Systematic reflection conditions and statistical tests of the data suggested the space group $P2_1/c$ *(# 14)* and was confirmed by ShelXT 2018/2 structure solution program using dual methods.[5,6] The structure was refined by full matrix least squares minimization on $\boldsymbol{F^2}$ using version 2019/1 of ShelXL 2019/1.[5,6] All non-hydrogen atoms were refined anisotropically. Hydrogen atom positions were calculated geometrically and refined using the riding model.

X-ray data collection, solution, and refinement for (POBOP)Ru(CO)$_2$H (6) (CCDC 2491265) – A Leica M80 microscope was used to identify a suitable single colorless plate-shaped crystal of suitable dimensions (0.10 × 0.07 × 0.02 mm^3) from a representative sample of crystals of the same habit. The crystal mounted on a nylon loop was then placed in a cold nitrogen stream (Oxford) maintained at T = 100.00(10) K. Crystal screening, unit cell determination, and data collection were carried out using a XtaLAB Synergy, Dualflex, HyPix diffractometer. The diffraction pattern was indexed and the total number of runs and images was based on the strategy calculation from the program CrysAlisPro system CrysAlisPro system (CCD 43.142a 64-bit (release 17-10-2024)).[3] Data were measured using *w* scans with Cu K_a radiation. Data was collected to a maximum resolution of Q = 79.693° (0.78 Å). The unit cell was refined using CrysAlisPro 1.171.43.143a on 17159 reflections, 56 % of the observed reflections. [1] Integrated Intensity information for each reflection was obtained by reduction of data frames using CrysAlisPro 1.171.43.143a.[3] The final completeness is 98.40 % out to 79.693° in Q. A gaussian absorption correction was performed using CrysAlisPro 1.171.43.143a.[3] Numerical absorption correction based on gaussian integration over a multifaceted crystal model Empirical absorption correction using spherical harmonics, implemented in SCALE3 ABSPACK scaling algorithm. The absorption coefficient *m* of this material is 4.679 mm^{-1} at this wavelength (l = 1.54184Å) and the minimum and maximum transmissions are 0.115 and 0.659. Systematic reflection conditions and statistical tests of the data suggested the space group $P2_1/n$ (# 14) and was confirmed by ShelXT 2018/2 structure solution program using dual methods.[5,6] The structure was refined by full matrix least squares minimization on $\boldsymbol{F^2}$ using version 2019/1 of ShelXL 2019/1.[5,6] All non-hydrogen atoms were refined anisotropically. Hydrogen atom positions were calculated geometrically and refined using the riding model.

X-ray data collection, solution, and refinement for $[NaRu(PO)_3]_2$ (7) (CCDC 2528011) – A Leica M80 microscope was used to identify a suitable single colorless plate-shaped crystal of suitable dimensions (0.43 × 0.15 × 0.05 mm^3) from a representative sample of crystals of the same habit. The crystal mounted on a nylon loop was then placed in a cold nitrogen stream (Oxford) maintained at T = 99.99(10) K. Crystal screening, unit cell determination, and data collection were carried out using a XtaLAB Synergy, Dualflex, HyPix diffractometer. The diffraction pattern was indexed and the total number of runs and images was based on the strategy calculation from the program CrysAlisPro system (CCD 43.142a 64-bit (release 17-10-2024)). [1] Data were measured using *w* scans with Cu K_a radiation. Data was collected to a maximum resolution of Q = 80.223° (0.78 Å). The unit cell was refined using CrysAlisPro 1.171.43.143a on 78455 reflections, 76 % of the observed reflections. [1] Integrated Intensity information for each reflection was obtained by reduction of data frames using CrysAlisPro 1.171.43.143a. [1] The final completeness is 99.90 % out to 80.223° in Q. A gaussian absorption correction was performed using CrysAlisPro 1.171.43.143a.[1] Numerical absorption correction based on gaussian integration over a multifaceted crystal model Empirical absorption correction using spherical harmonics, implemented in SCALE3 ABSPACK scaling algorithm. The absorption coefficient *m* of this material is 4.744 mm^{-1} at this wavelength (l = 1.54184Å) and the minimum and maximum transmissions are 0.356 and 1.000. Systematic reflection conditions and statistical tests of the data suggested the space group $P2_1/c$ (# 14) and was confirmed by ShelXT 2018/2 structure solution program using dual methods. [3,4] The structure was refined by full matrix least squares minimization on $\boldsymbol{F^2}$ using version 2019/1 of ShelXL 2019/1.[3,4] All non-hydrogen atoms were refined anisotropically. Hydrogen atom positions were calculated geometrically and refined using the riding model.

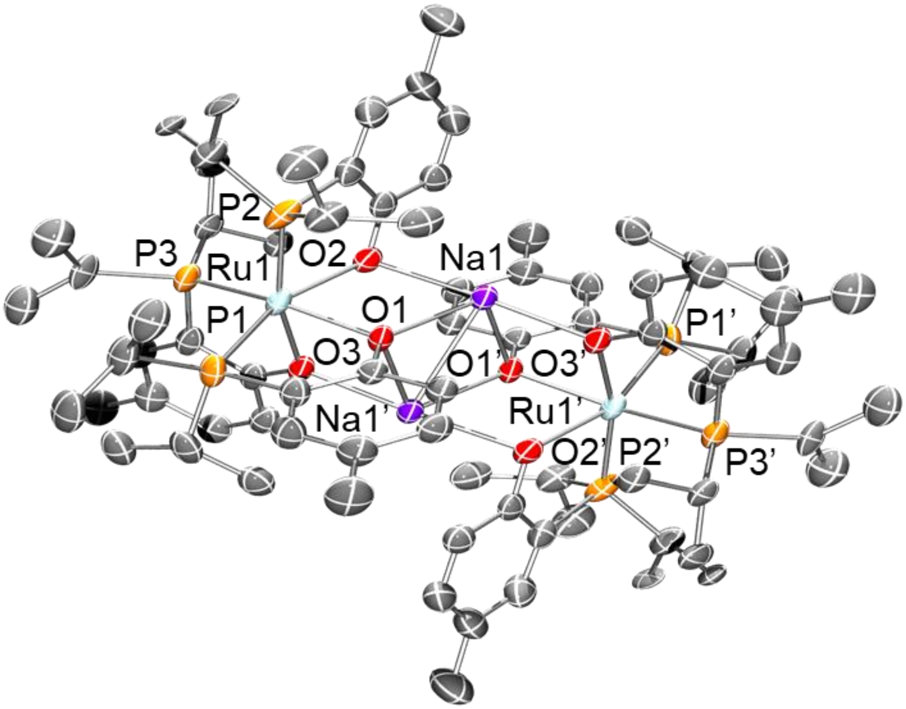

Figure S1. POV-Ray rendition of the ORTEP drawing (50% thermal ellipsoids) of **7** showing selected atom labeling. Hydrogen atoms are omitted for clarity. Selected bond lengths (Å) and angles (°) for **7**: Ru1–P1, 2.3426(8); Ru1–P2, 2.2975(10); Ru1–P3, 2.3053(8); Ru1–O1, 2.1459(18); Ru1–O2, 2.1070(17); Ru1–O3, 2.128 (2); Na1–O1, 2.362(2); Na1–O3, 2.266(2); P1–Ru1–P2, 167.378(17); P1–Ru1–O2, 160.51(5); P2–Ru1–O3, 164.12(5); P3–Ru1–O1, 163.91(6).

IV. NMR and IR spectra

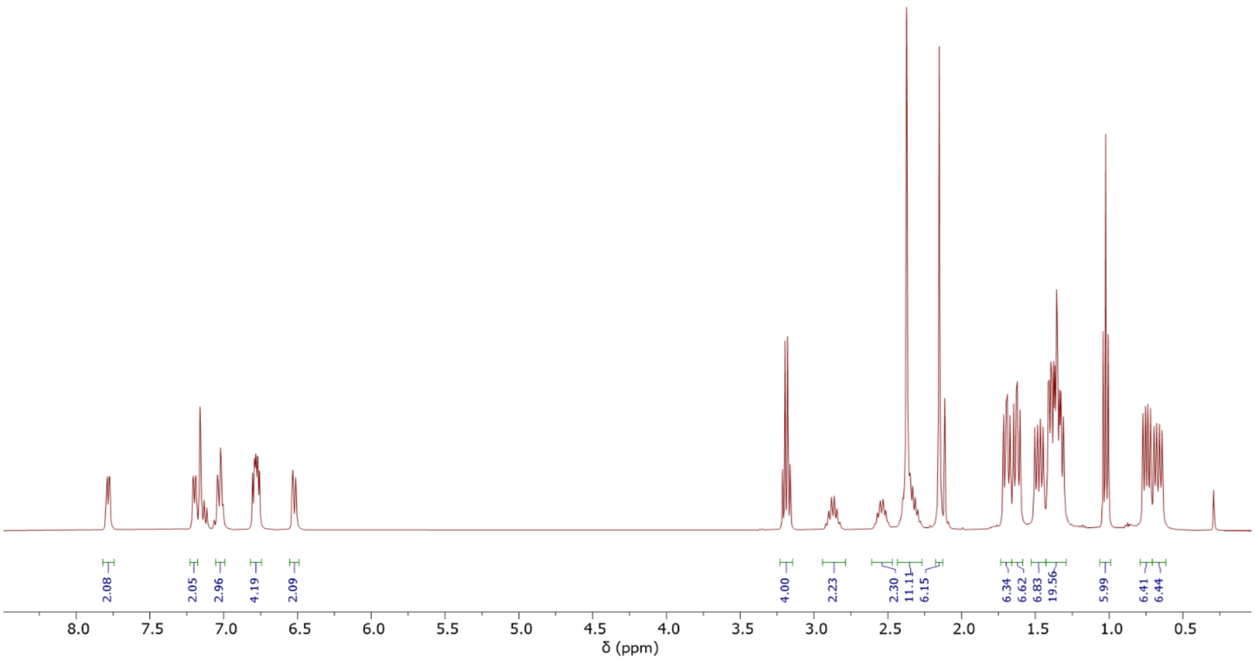

Figure S2. ^{1}H NMR (400 MHz, C_6D_6) spectrum of **2**. Sample contains residual toluene, pentane, and silicone grease.

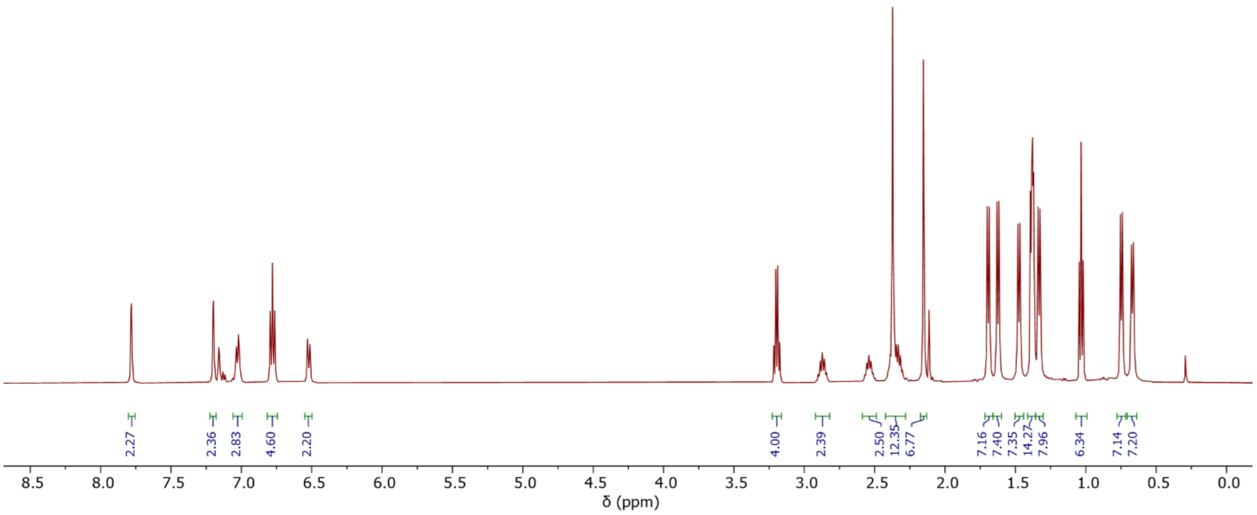

Figure S3. $^{1}H\{^{31}P\}$ NMR (500 MHz, C_6D_6) spectrum of **2**. Sample contains residual toluene, pentane, and silicone grease.

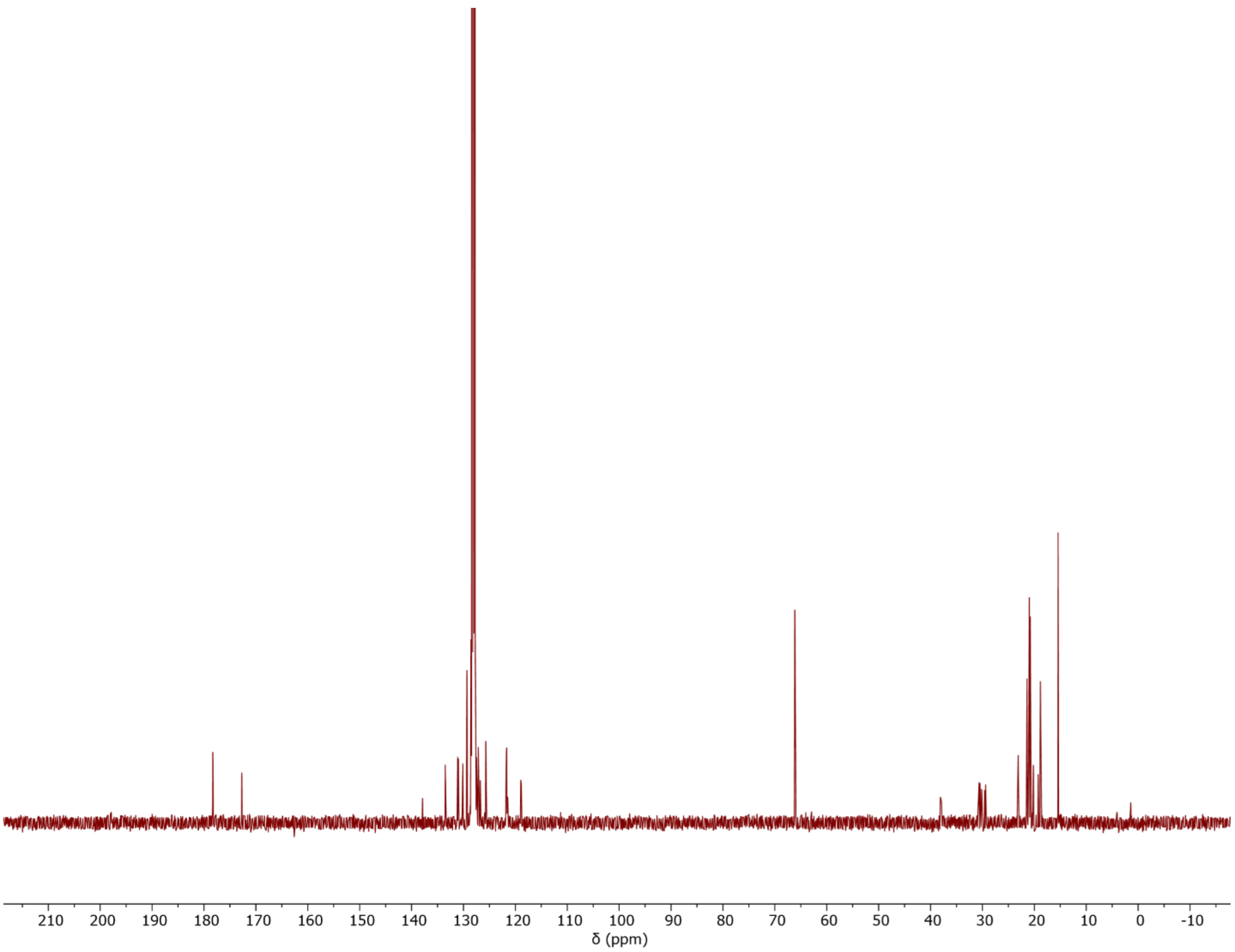

Figure S4. $^{13}C\{^1H\}$ NMR (101 MHz, C_6D_6) spectrum of **2**. Sample contains residual toluene, pentane, and silicone grease.

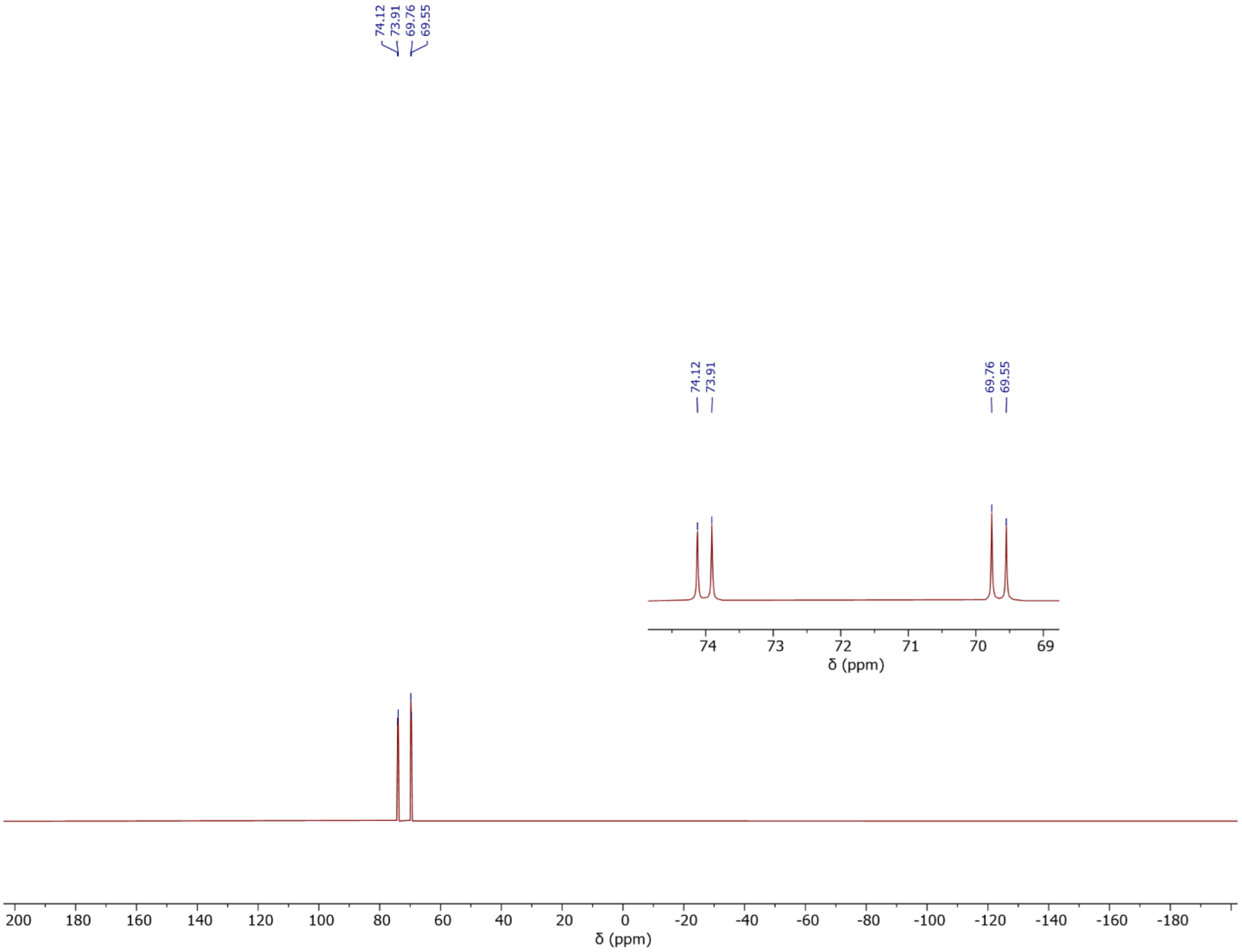

Figure S5. $^{31}P\{^{1}H\}$ NMR (162 MHz, C_6D_6) spectrum of **2**.

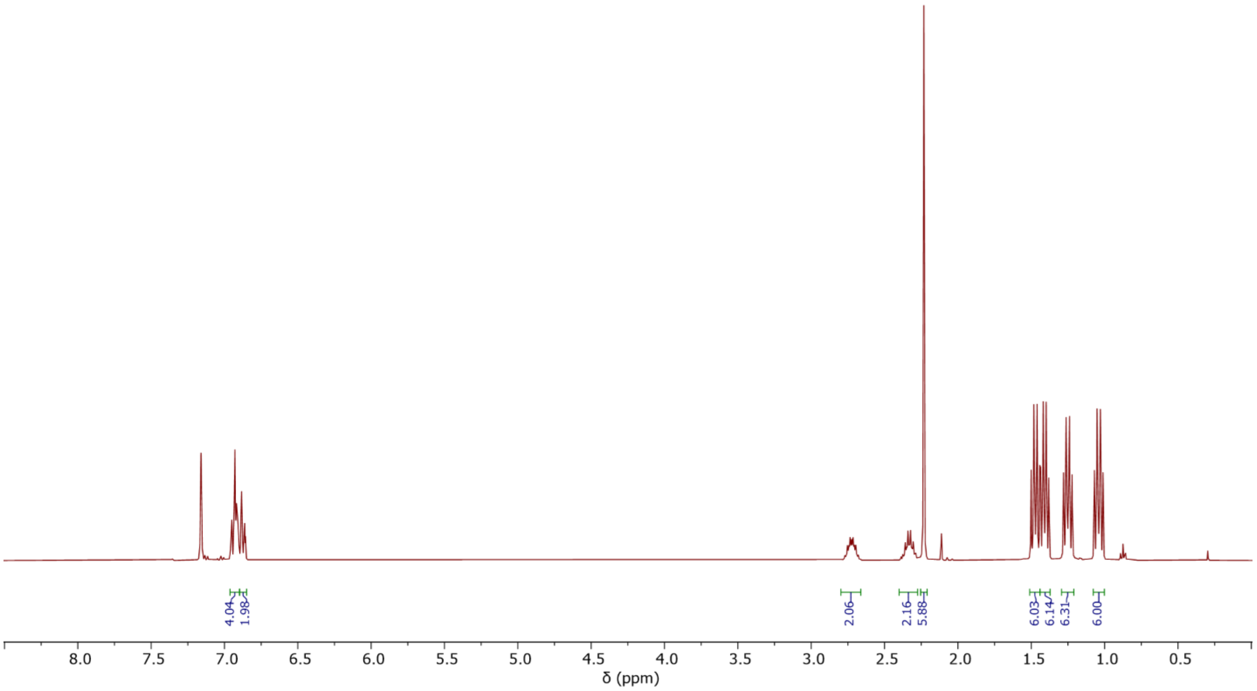

Figure S6. ^{1}H NMR (400 MHz, C_6D_6) spectrum of **3**. Sample contains residual toluene, pentane, and silicone grease.

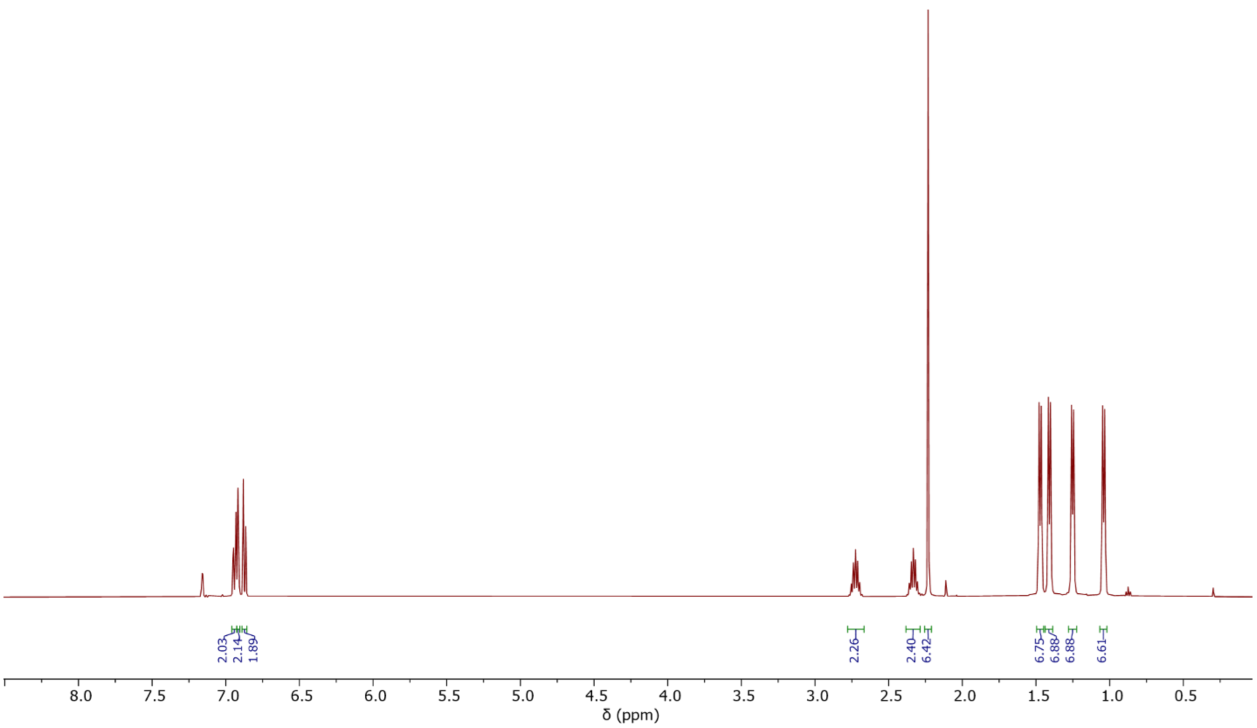

Figure S7. $^{1}H\{^{31}P\}$ NMR (500 MHz, C_6D_6) spectrum of **3**. Sample contains residual toluene, pentane, and silicone grease.

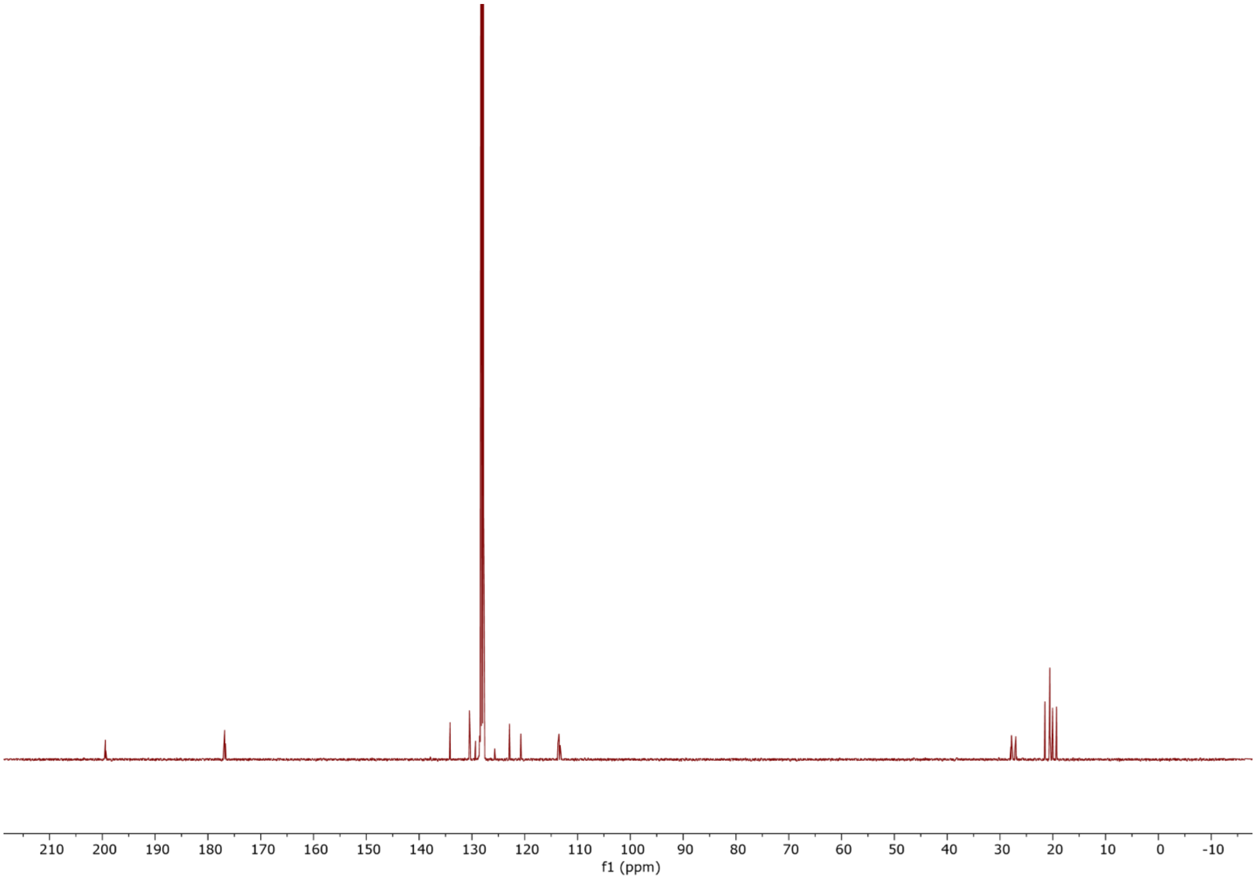

Figure S8. $^{13}C\{^{1}H\}$ NMR (101 MHz, C_6D_6) spectrum of **3**. Sample contains residual toluene, pentane, and silicone grease.

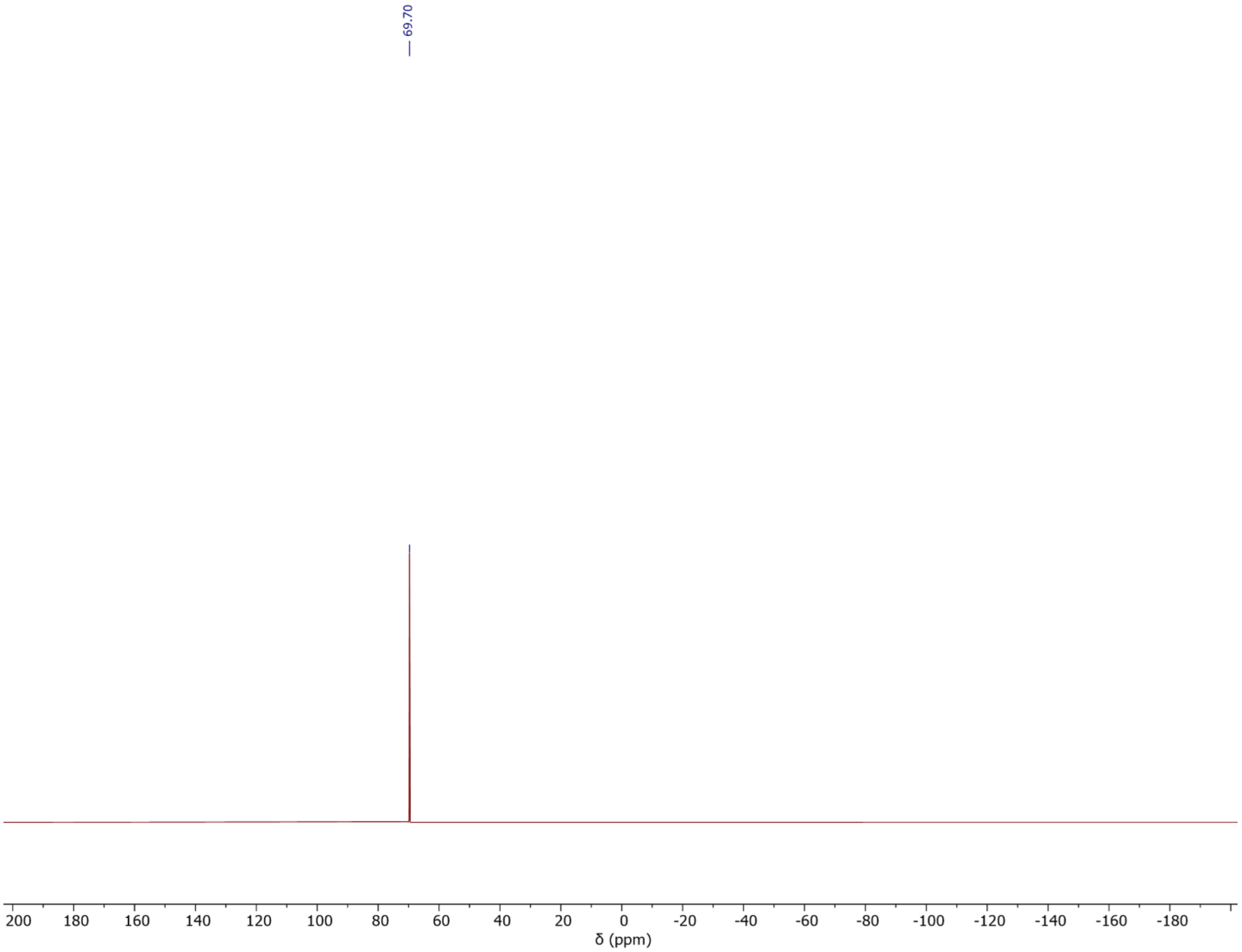

Figure S9. $^{31}P\{^{1}H\}$ NMR (162 MHz, C_6D_6) spectrum of **3**.

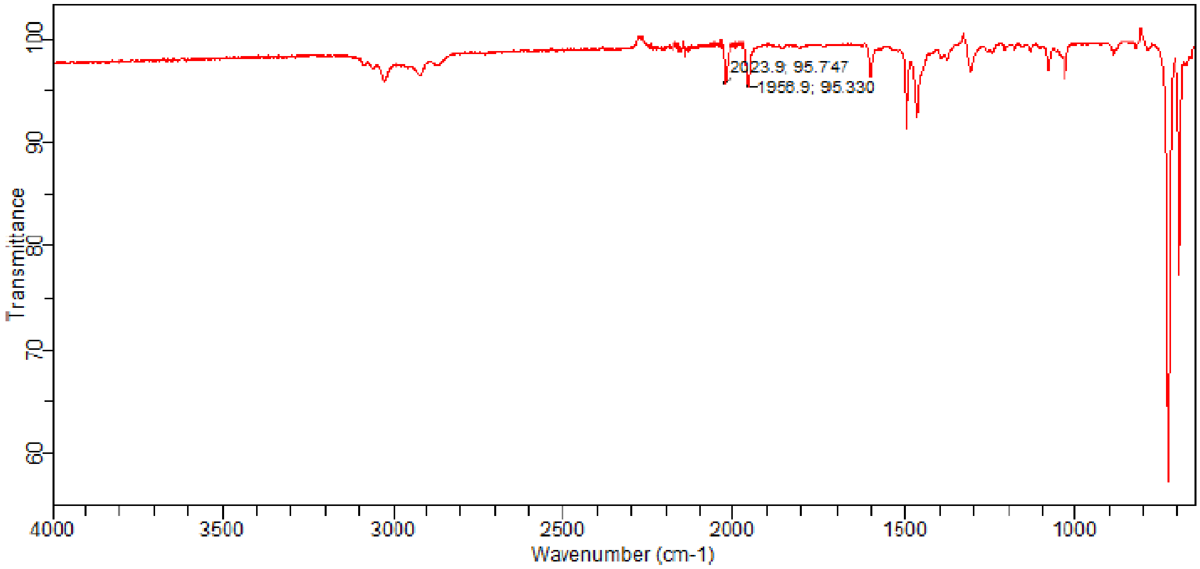

Figure S10. ATR-IR Spectrum of **3**.

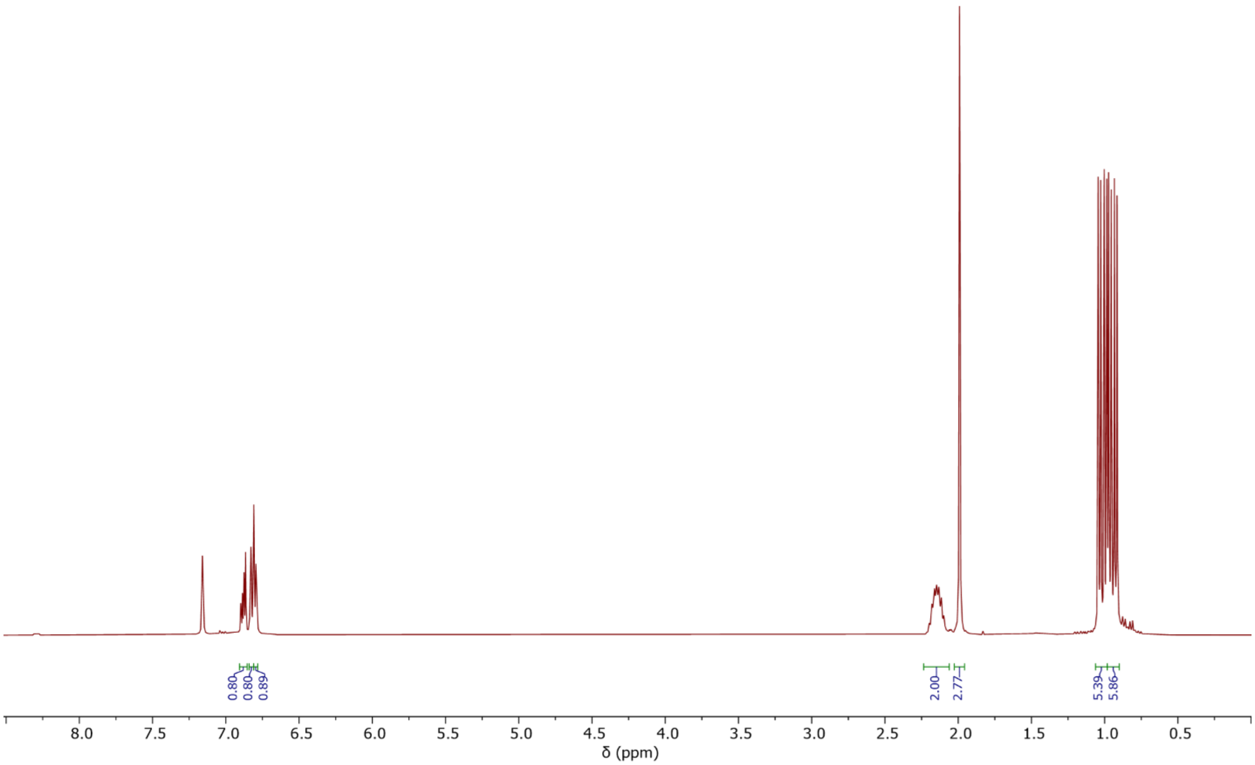

Figure S11. ^{1}H NMR (400 MHz, C_6D_6) spectrum of **4**.

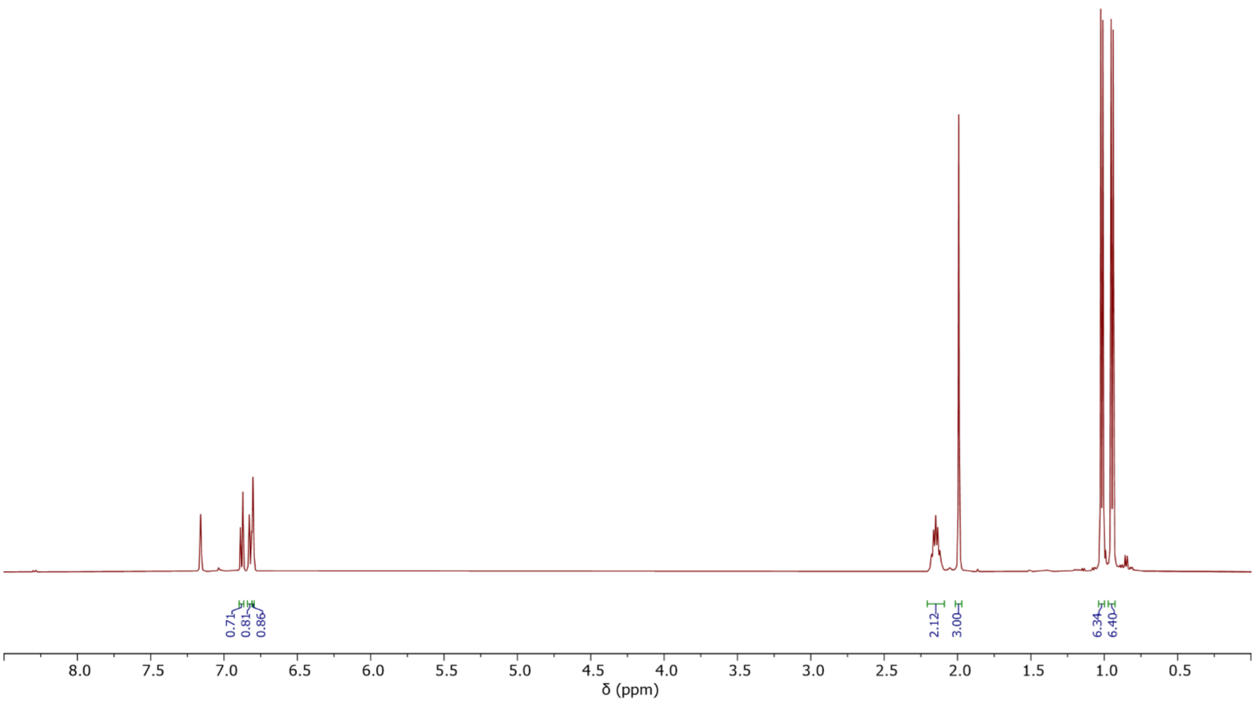

Figure S12. $^{1}H\{^{31}P\}$ NMR (500 MHz, C_6D_6) spectrum of **4**.

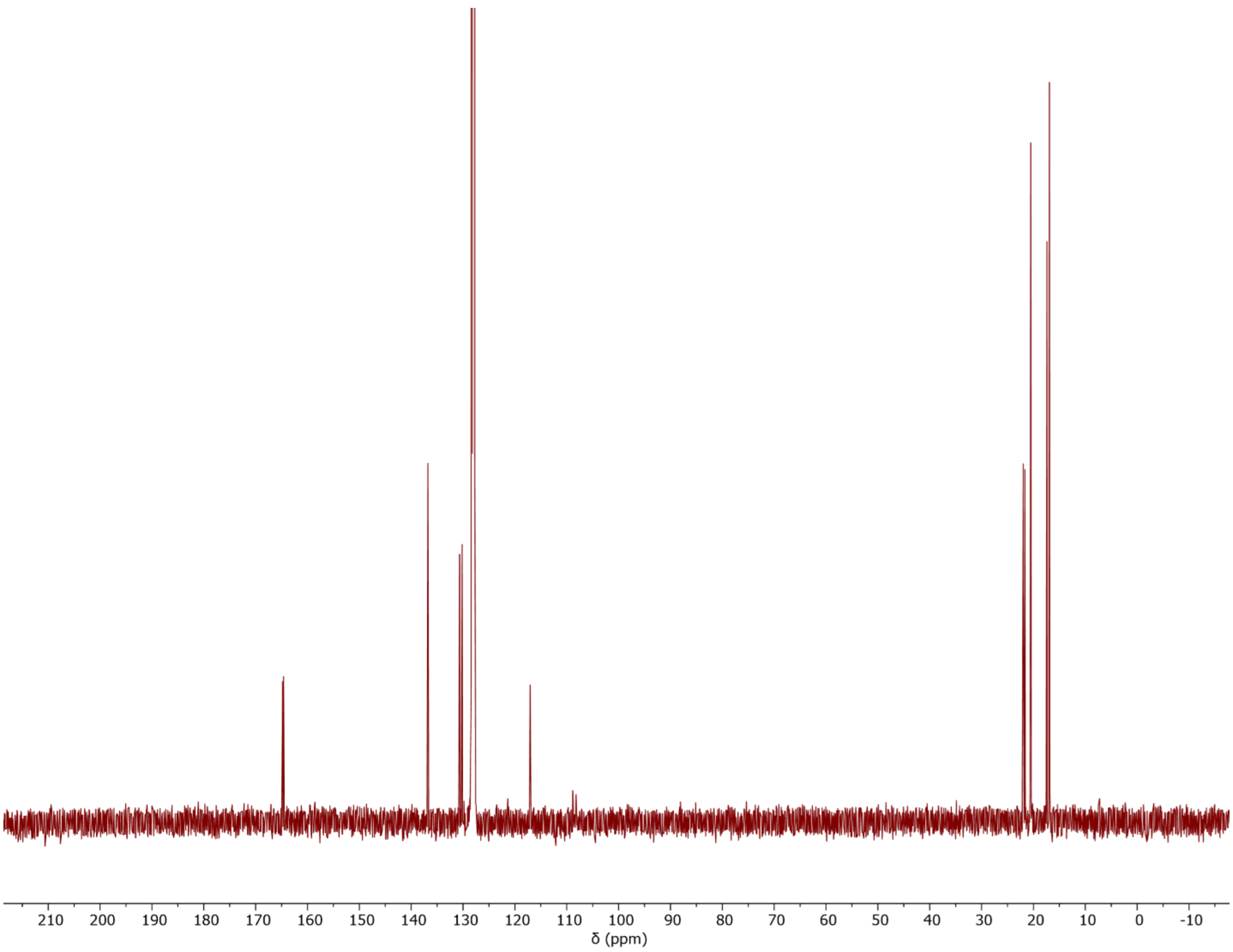

Figure S13. $^{13}C\{^1H\}$ NMR (101 MHz, C_6D_6) spectrum of **4**.

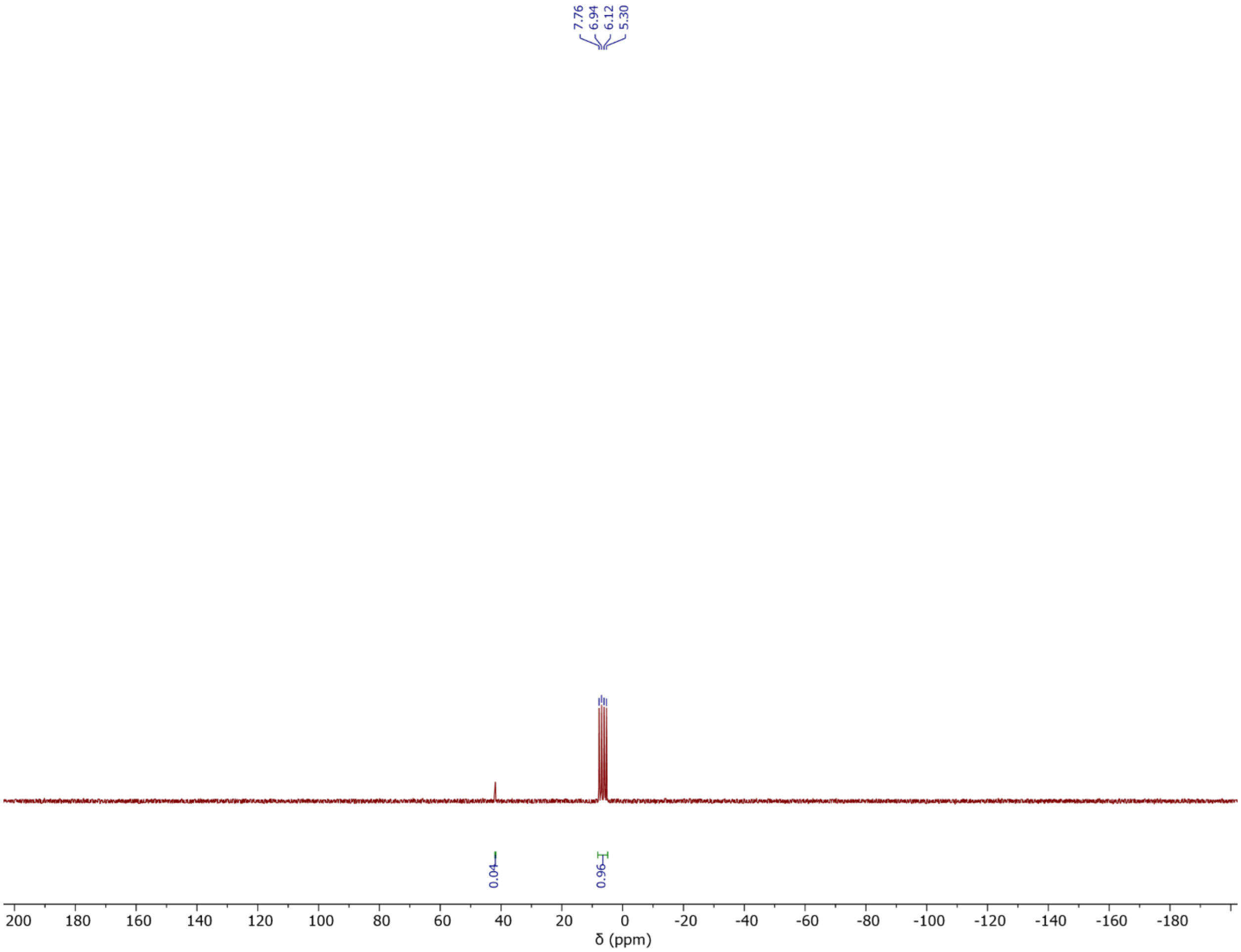

Figure S14. $^{31}P\{^{1}H\}$ NMR (162 MHz, C_6D_6) spectrum of **4**. Spectrum contains an unknown impurity (δ 48.9 ppm).

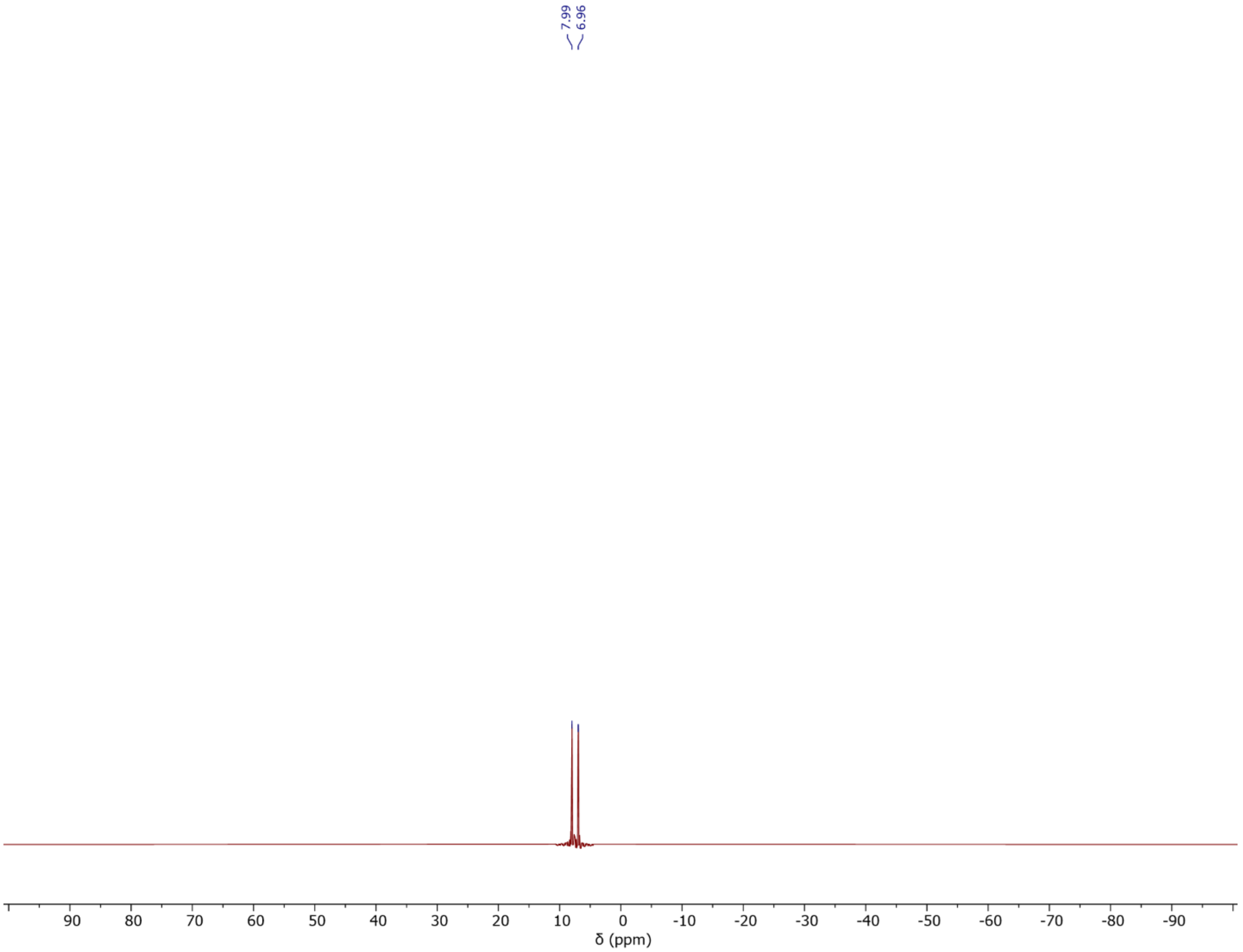

Figure S15. $^{11}B\{^{1}H\}$ NMR (128 MHz, C_6D_6) spectrum of **4**.

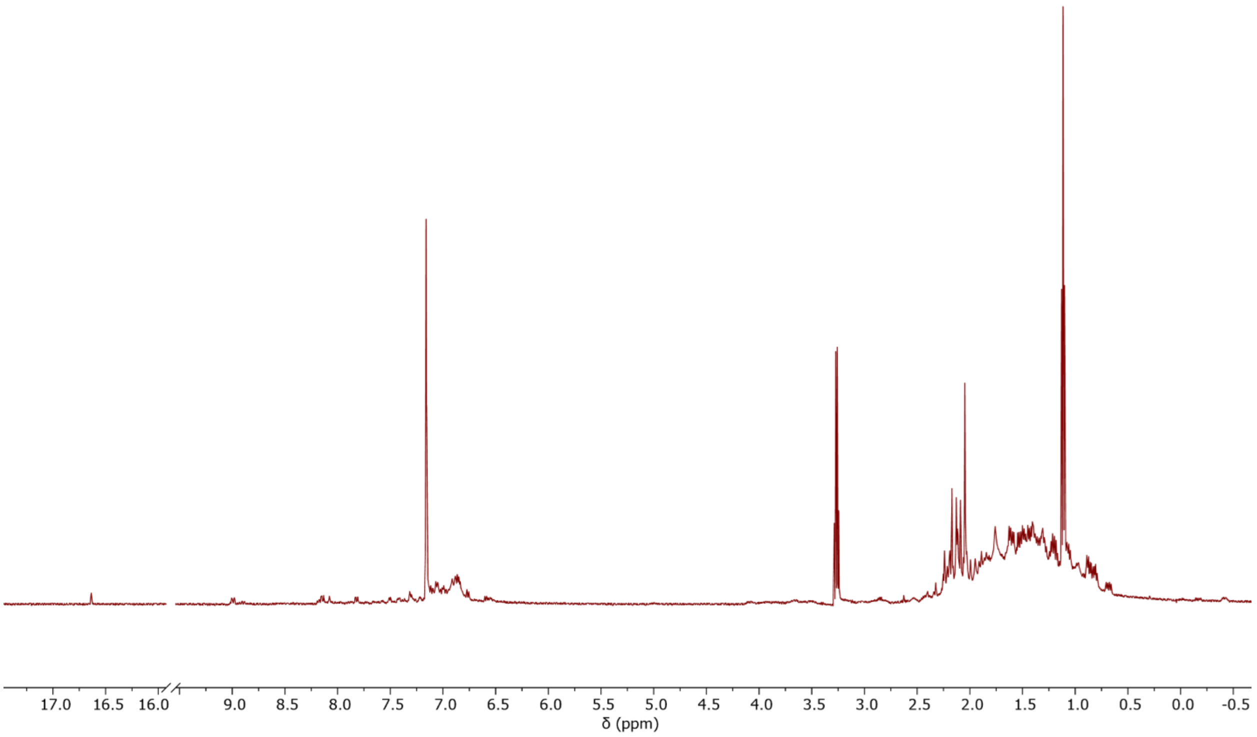

Figure S16. ^{1}H NMR (500 MHz, C_6D_6) spectrum of the reaction between **2** and BCl_3•SMe_2 after ~16 h at room temperature.

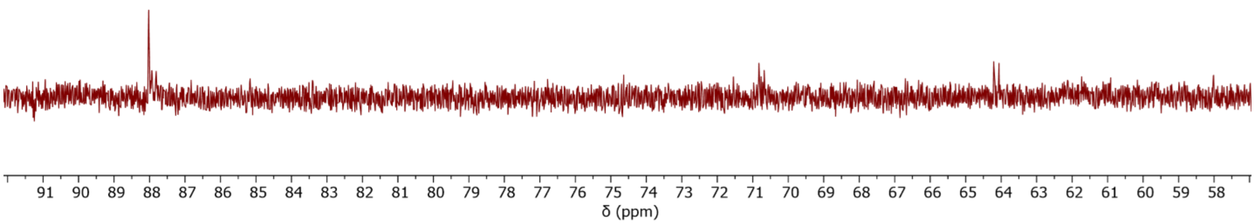

Figure S17. $^{31}P\{^1H\}$ NMR (202 MHz, C_6D_6) spectrum of the reaction between **2** and BCl_3•SMe_2 after ~16 h at room temperature.

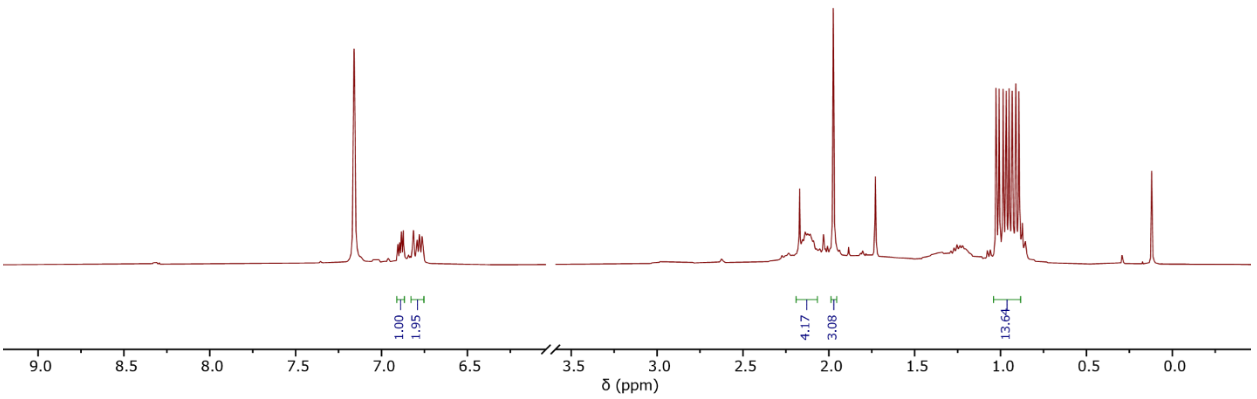

Figure S18. ^{1}H NMR (400 MHz, C_6D_6) spectrum of the reaction between **2** and BCl_3•SMe_2 after heating at 90 °C for ~16 h.

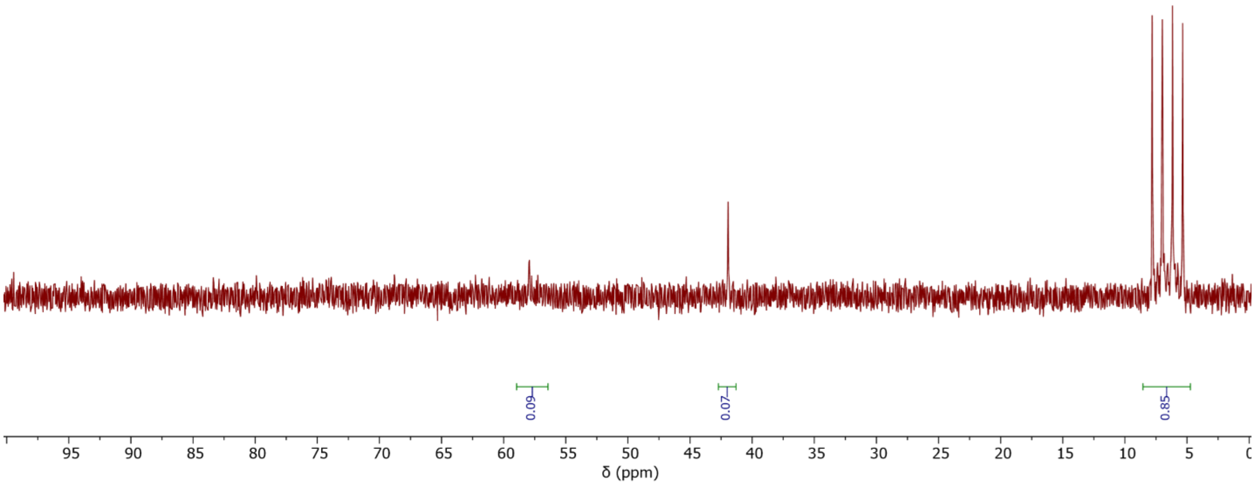

Figure S19. $^{31}P\{^{1}H\}$ NMR (162 MHz, C_6D_6) spectrum of the reaction between **2** and BCl_3•SMe_2 after heating at 90 °C for ~16 h.

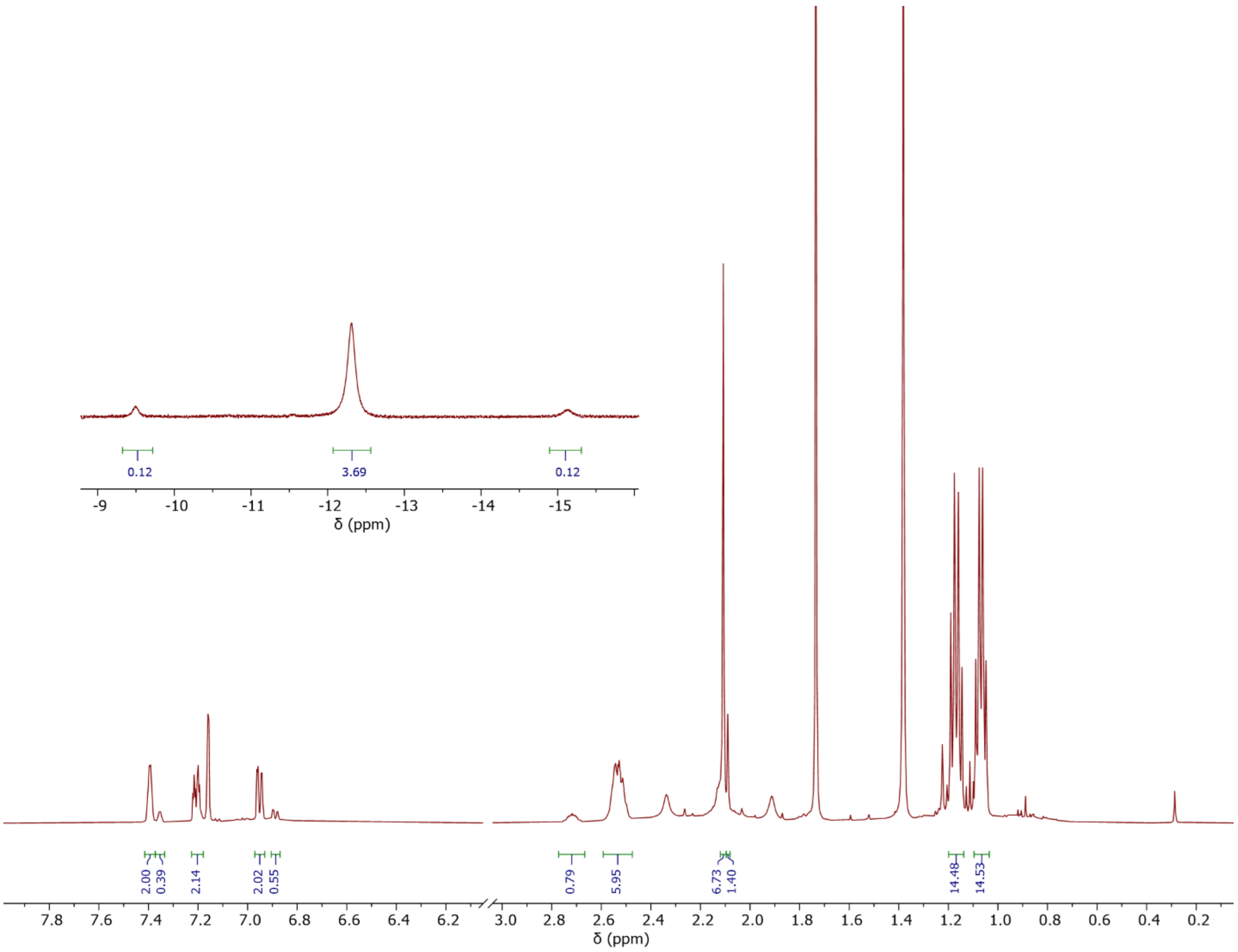

Figure S20. ^{1}H NMR (500 MHz, C_6D_6) spectrum of the reaction of **2** and BH_3•SMe_2 (10 eq.) in C_6D_6. Two products are observed in a 5:1 ratio, along with residual BH_3•SMe_2 and SMe_2.

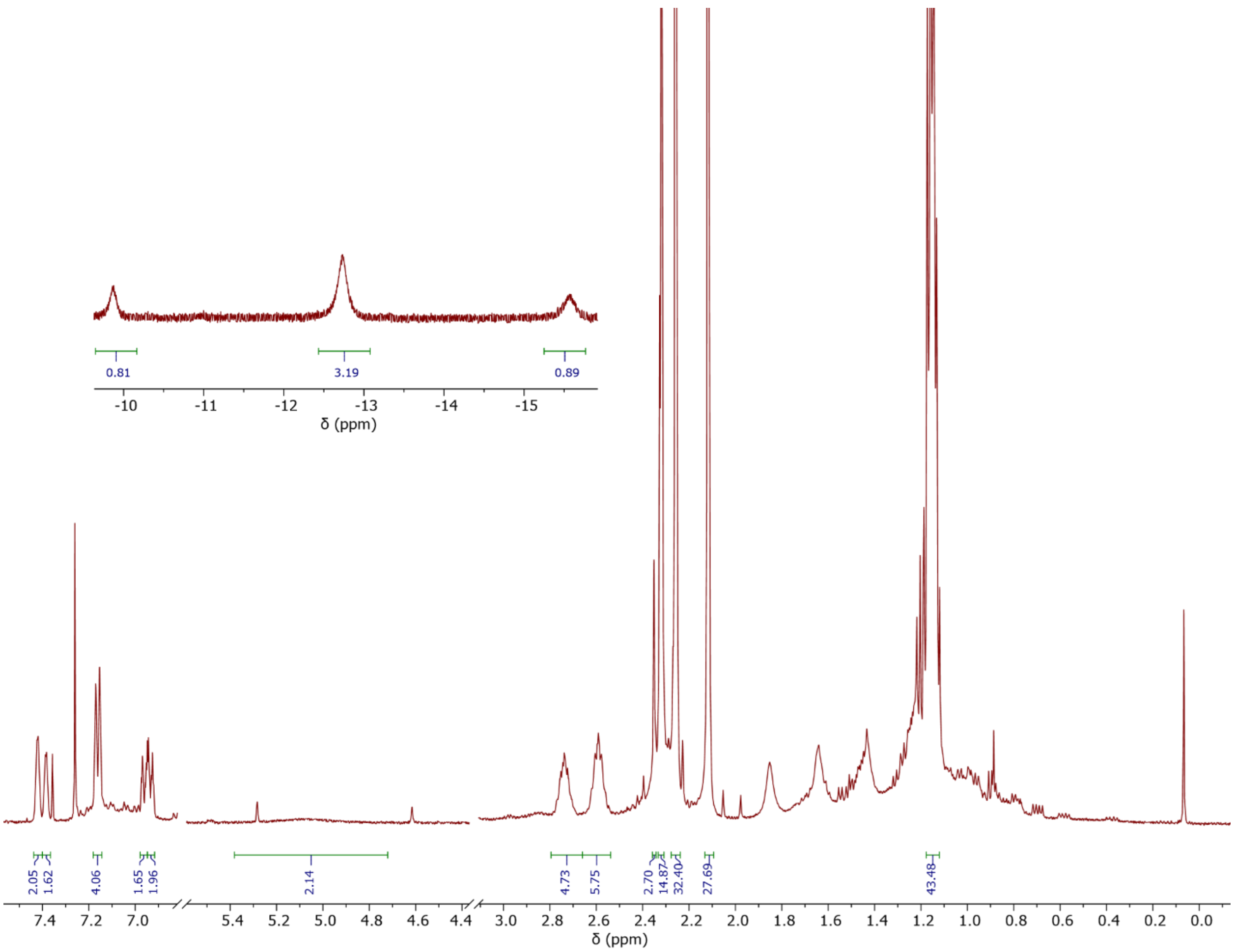

Figure S21. ^{1}H NMR (500 MHz, C_6D_6) spectrum of the reaction of **2** and BH_3•SMe_2 (10 eq.) in $CDCl_3$. Two products are observed in a 1:1 ratio, along with residual BH_3•SMe_2 and SMe_2.

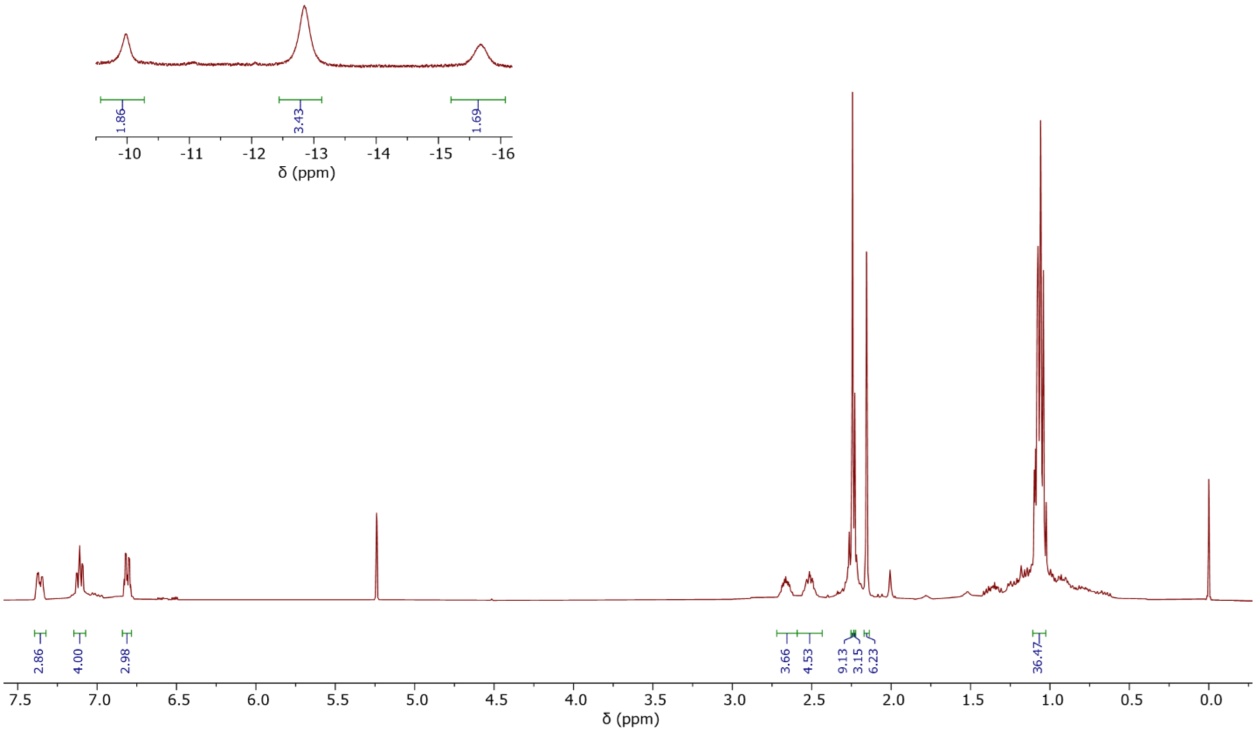

Figure S22. ^{1}H NMR (500 MHz, CD_2Cl_2) spectrum of the reaction of **2** and BH_3•SMe_2 in $CDCl_3$ after removing volatiles and redissolving in CD_2Cl_2. No change in the ratio of products is observed.

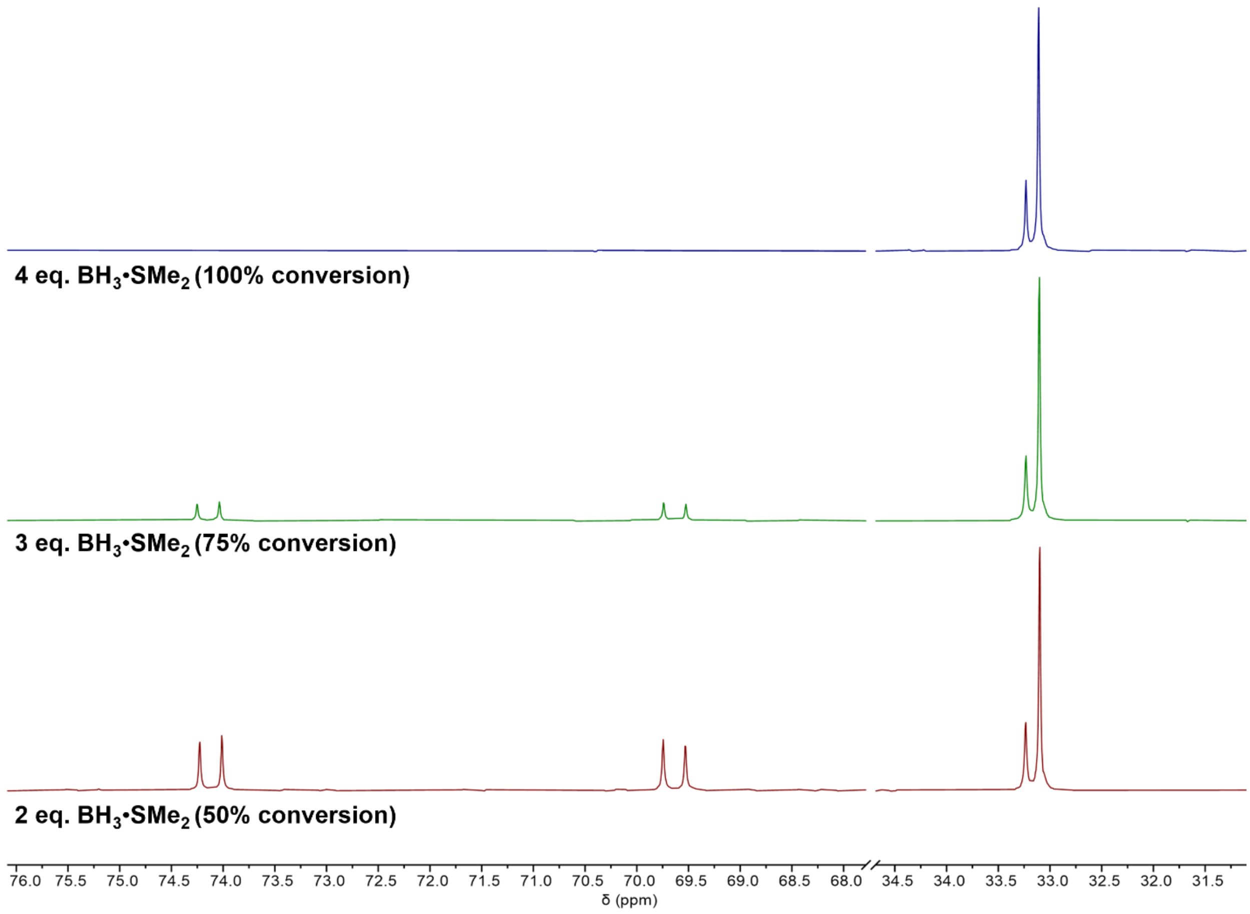

Figure S23. Stacked $^{31}P\{^{1}H\}$ NMR (162 MHz, C_6D_6) spectra of the reaction of **2** and different equivalents of BH_3•SMe_2, with varying conversion to **5a** and **5b**.

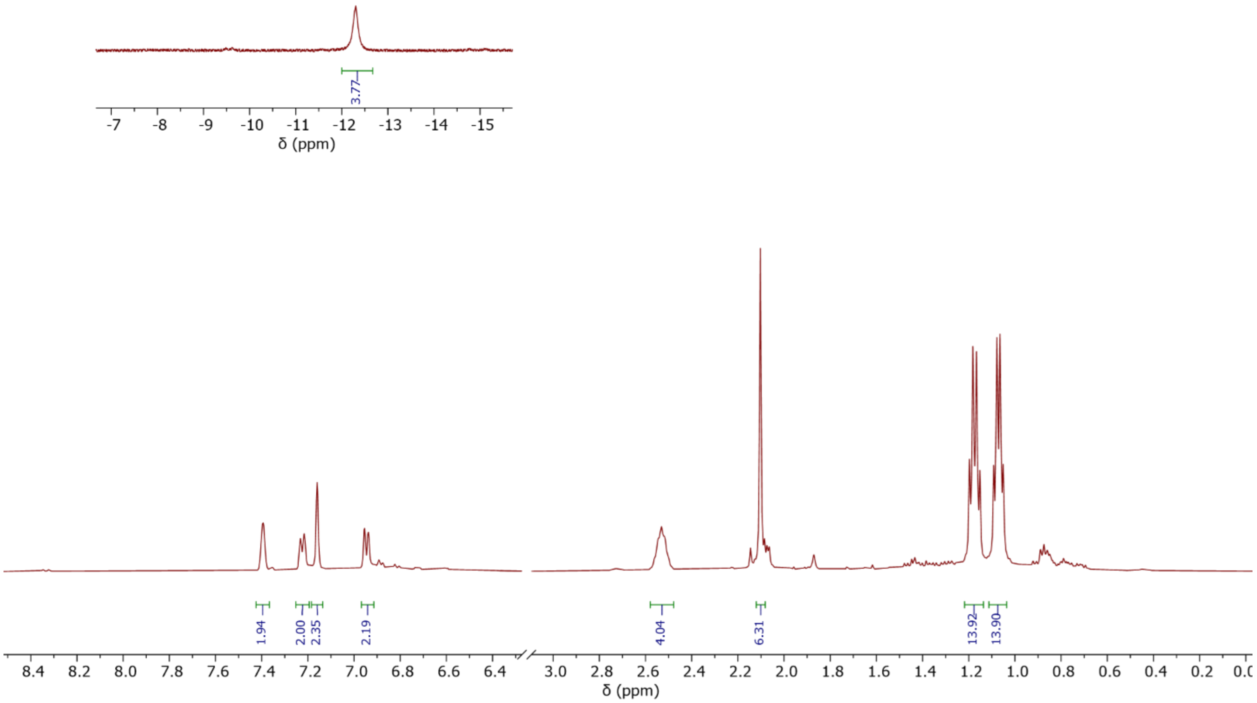

Figure S24. ^{1}H NMR (500 MHz, C_6D_6) spectrum of **5a**.

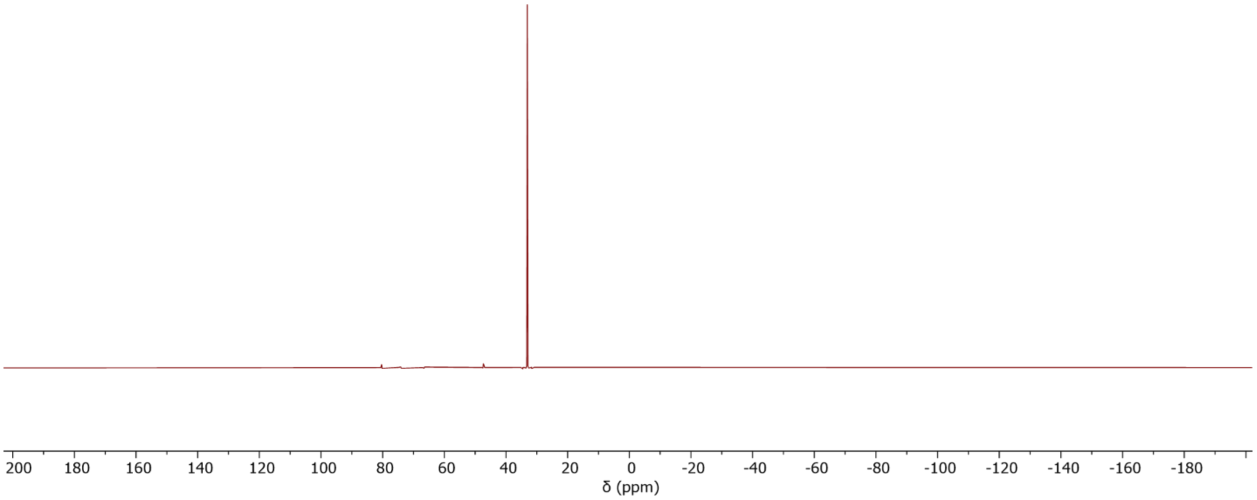

Figure S25. $^{31}P\{^{1}H\}$ NMR (162 MHz, C_6D_6) spectrum of **5a**.

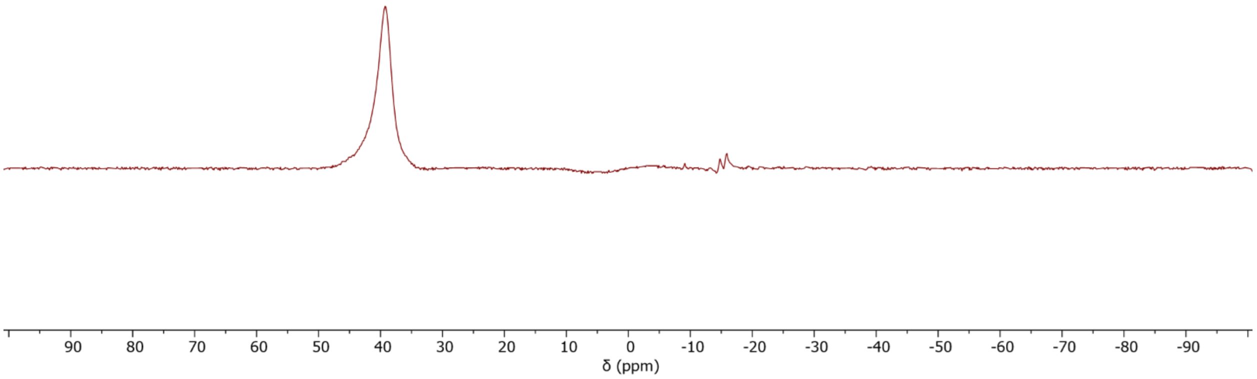

Figure S26. $^{11}B\{^{1}H\}$ NMR (128 MHz, C_6D_6) spectrum of **5a**.

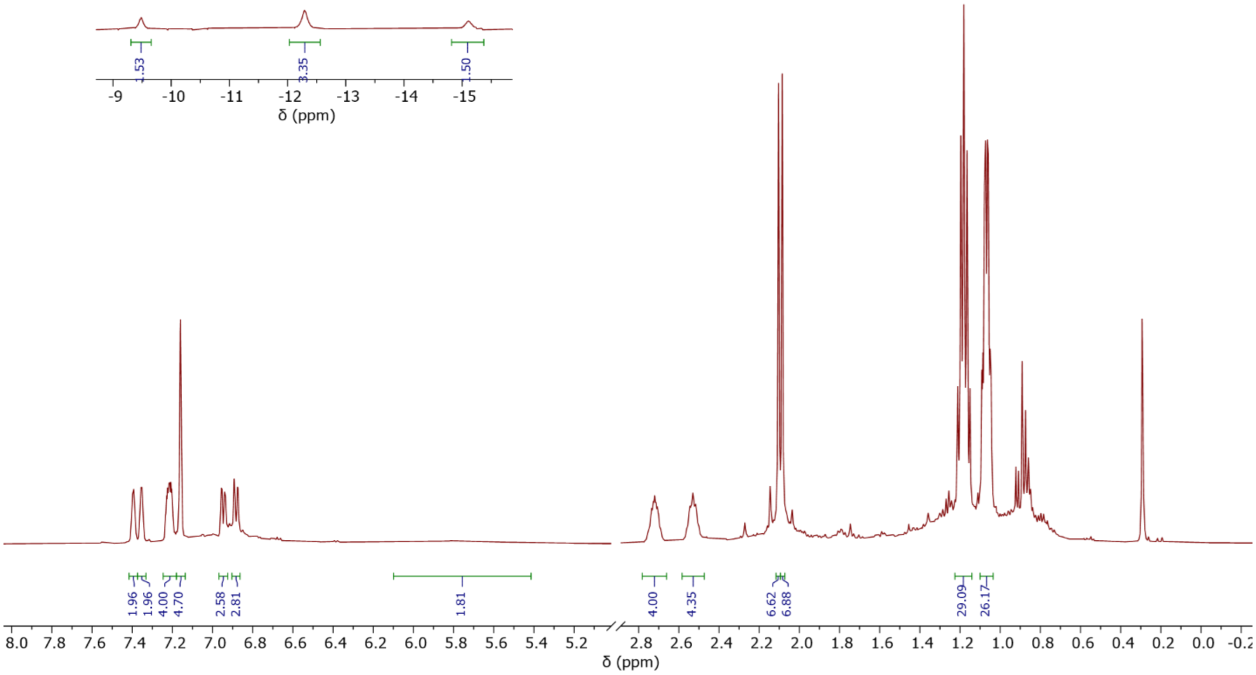

Figure S27. ¹H NMR (500 MHz, C_6D_6) spectrum of the pentane washes from the reaction **2** and BH_3•SMe_2 in toluene. Spectrum contains a mixture of **5a** and **5b**.

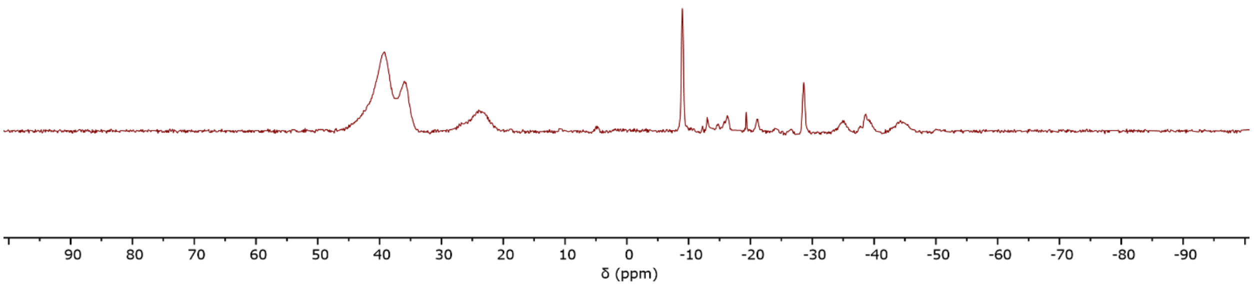

Figure S28. $^{11}B\{^1H\}$ NMR (128 MHz, C_6D_6) spectrum of the pentane washes from the reaction **2** and BH_3•SMe_2 in toluene. Spectrum contains a mixture of **5a** and **5b**, along with other unidentified species.

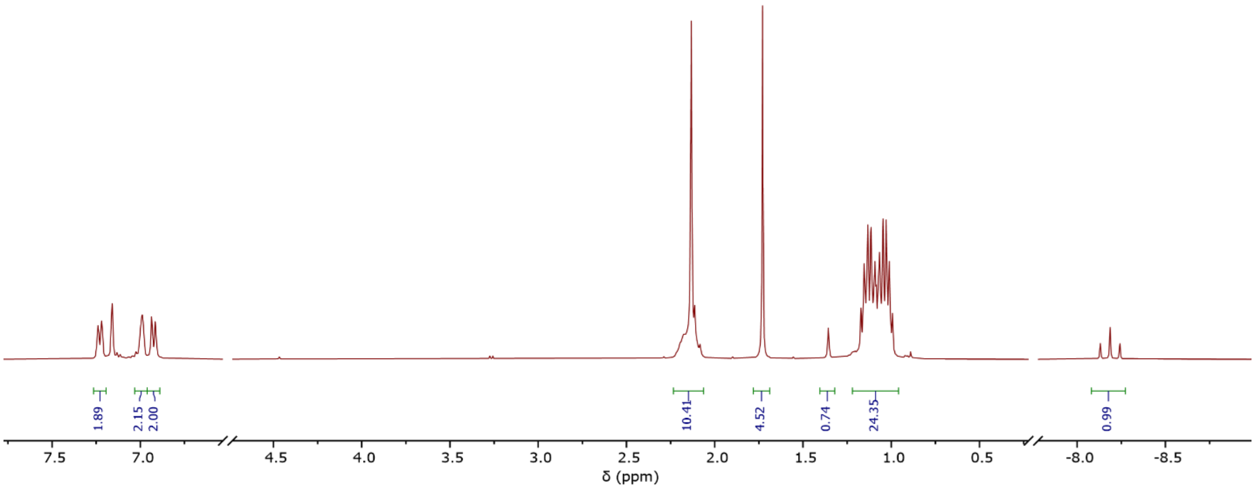

Figure S29. ^{1}H NMR (400 MHz, C_6D_6) spectrum of the initial observation of the clean formation of **5** from **3**. Spectrum contains free SMe_2 (δ 1.73 ppm) and a small amount of BH_3•SMe_2 (δ 1.36 ppm).

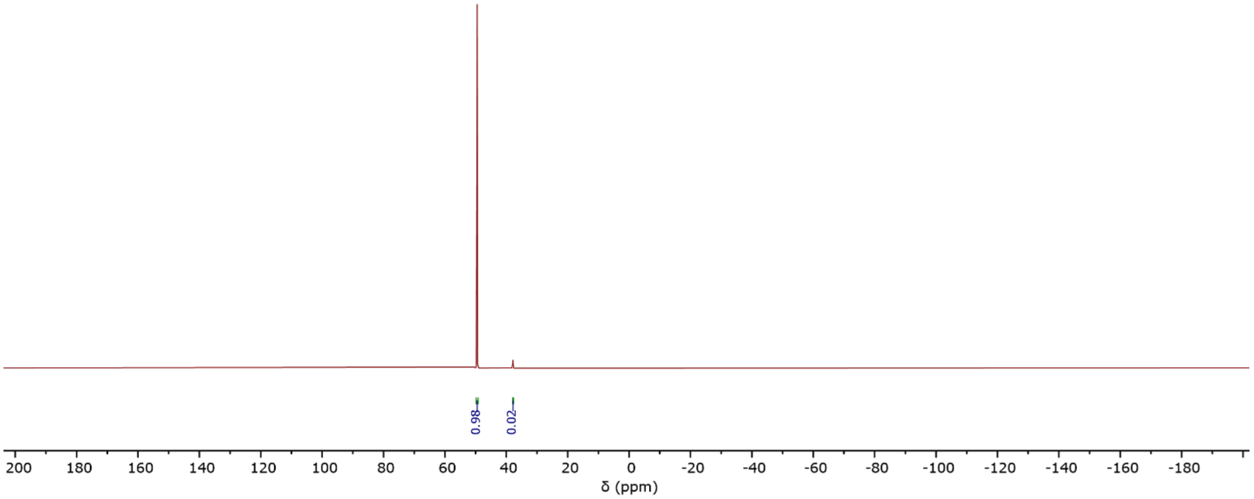

Figure S30. $^{31}P\{^{1}H\}$ NMR (162 MHz, C_6D_6) spectrum of the initial observation of the clean formation of **6** from **3**.

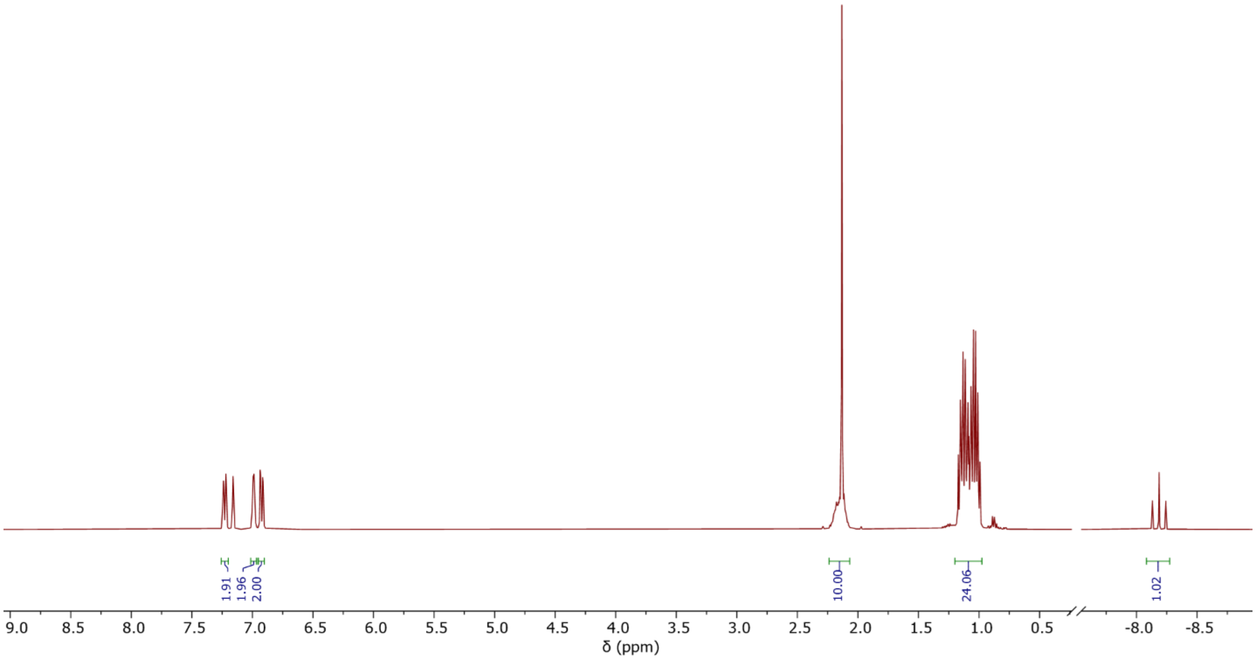

Figure S31. ^{1}H NMR (400 MHz, C_6D_6) spectrum of **6**. Sample contains residual pentane.

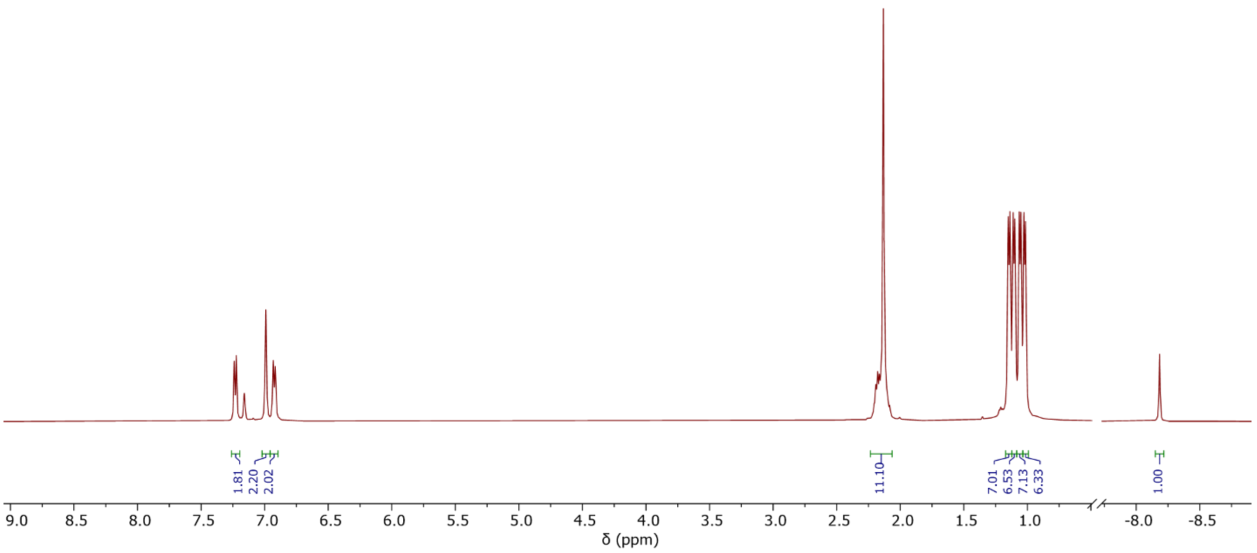

Figure S32. $^{1}H\{^{31}P\}$ NMR (500 MHz, C_6D_6) spectrum of **6**. Sample contains residual pentane.

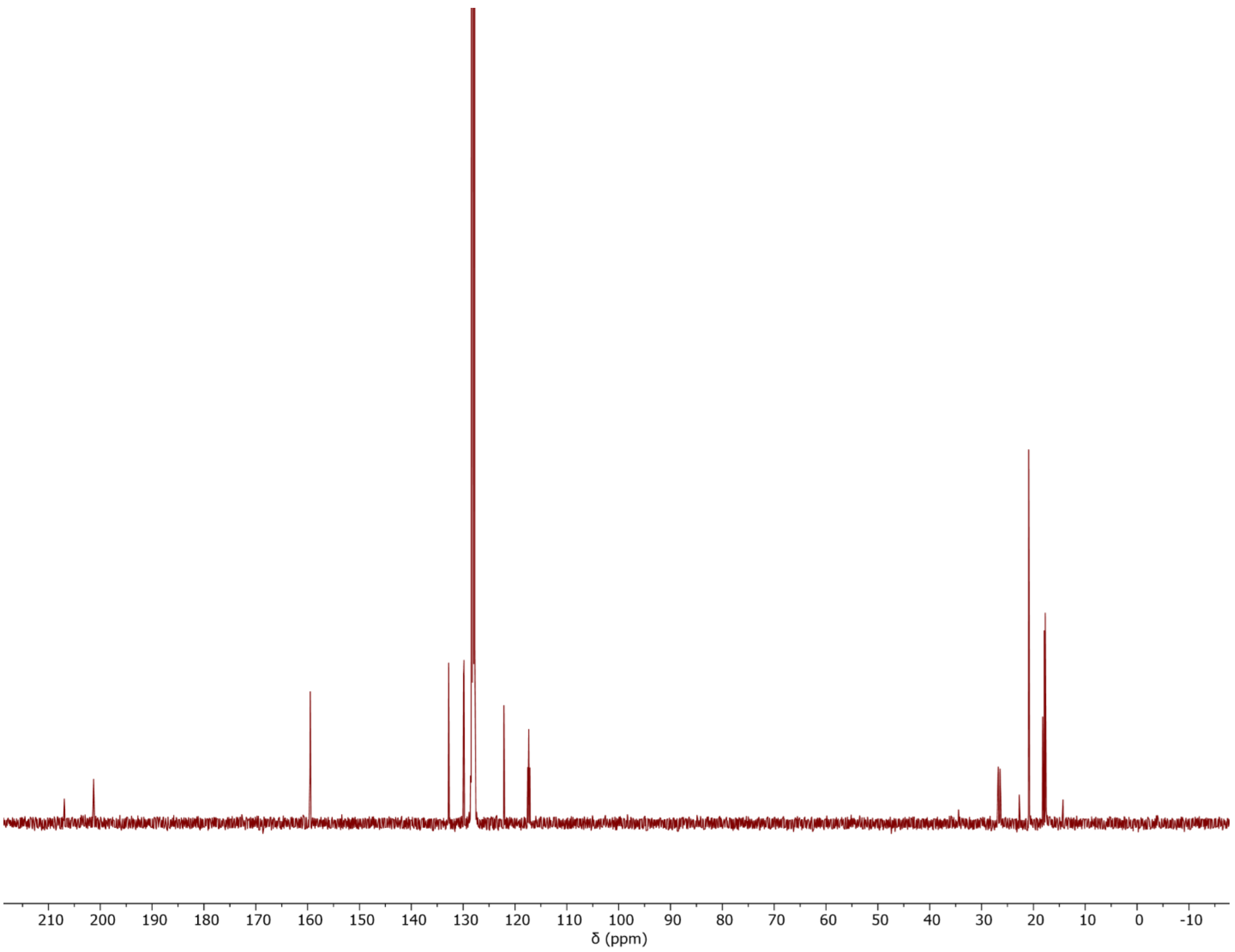

Figure S33. $^{13}C\{^{1}H\}$ NMR (101 MHz, C_6D_6) spectrum of **6**. Sample contains residual pentane.

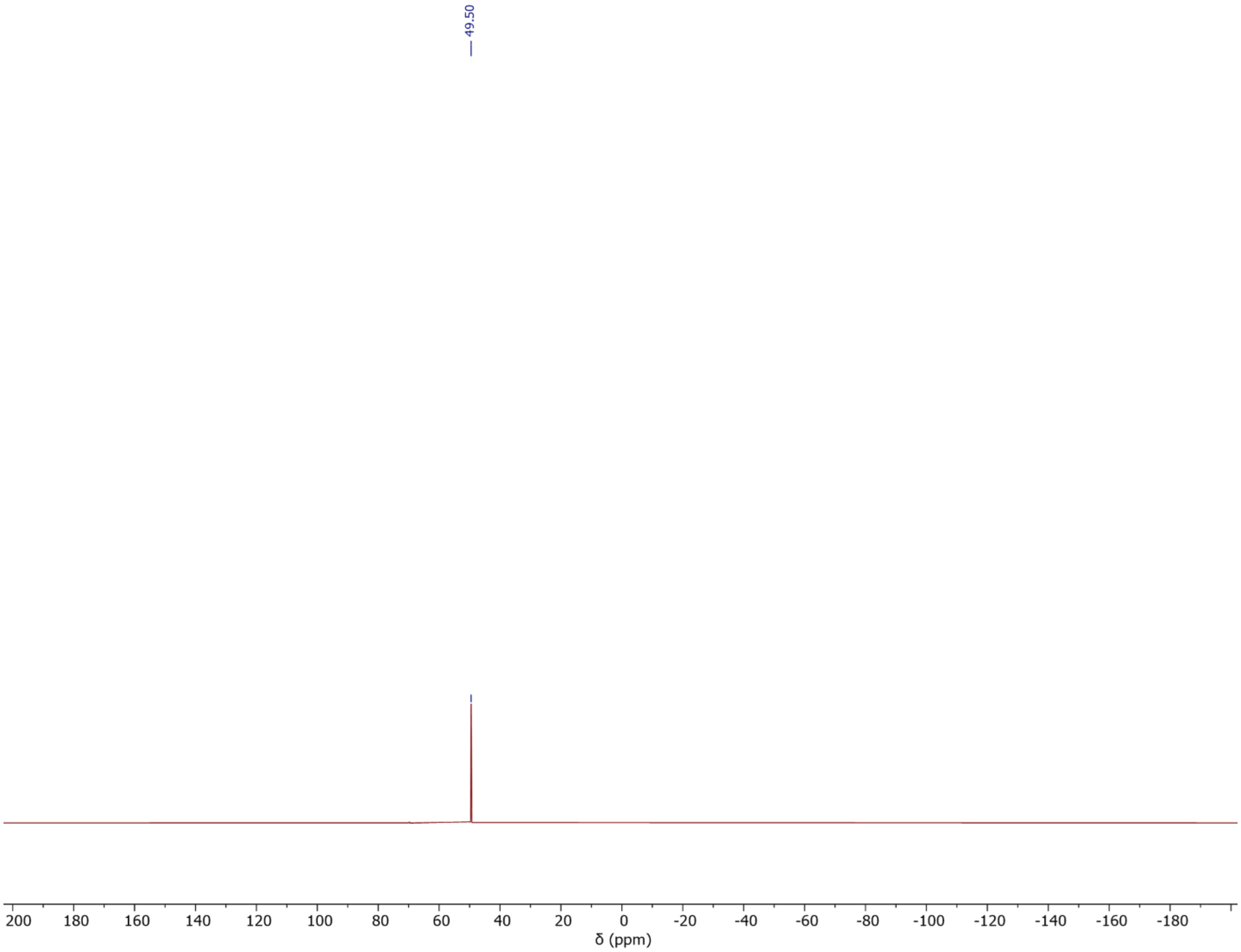

Figure S34. $^{31}P\{^{1}H\}$ NMR (162 MHz, C_6D_6) spectrum of **6**.

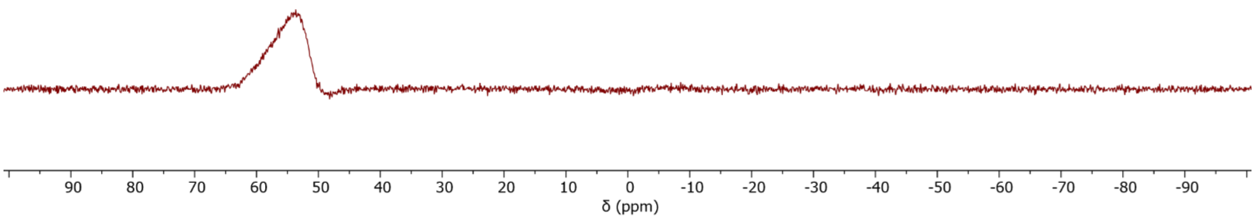

Figure S35. $^{11}B\{^1H\}$ NMR (128 MHz, C_6D_6) spectrum of **6**.

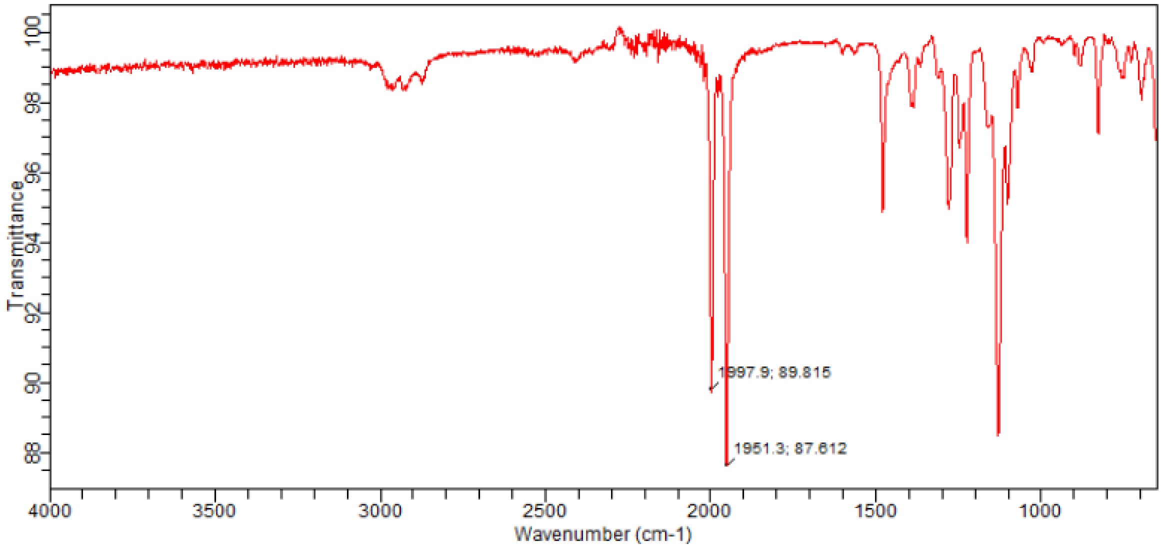

Figure S36. ATR-IR Spectrum of **6**.

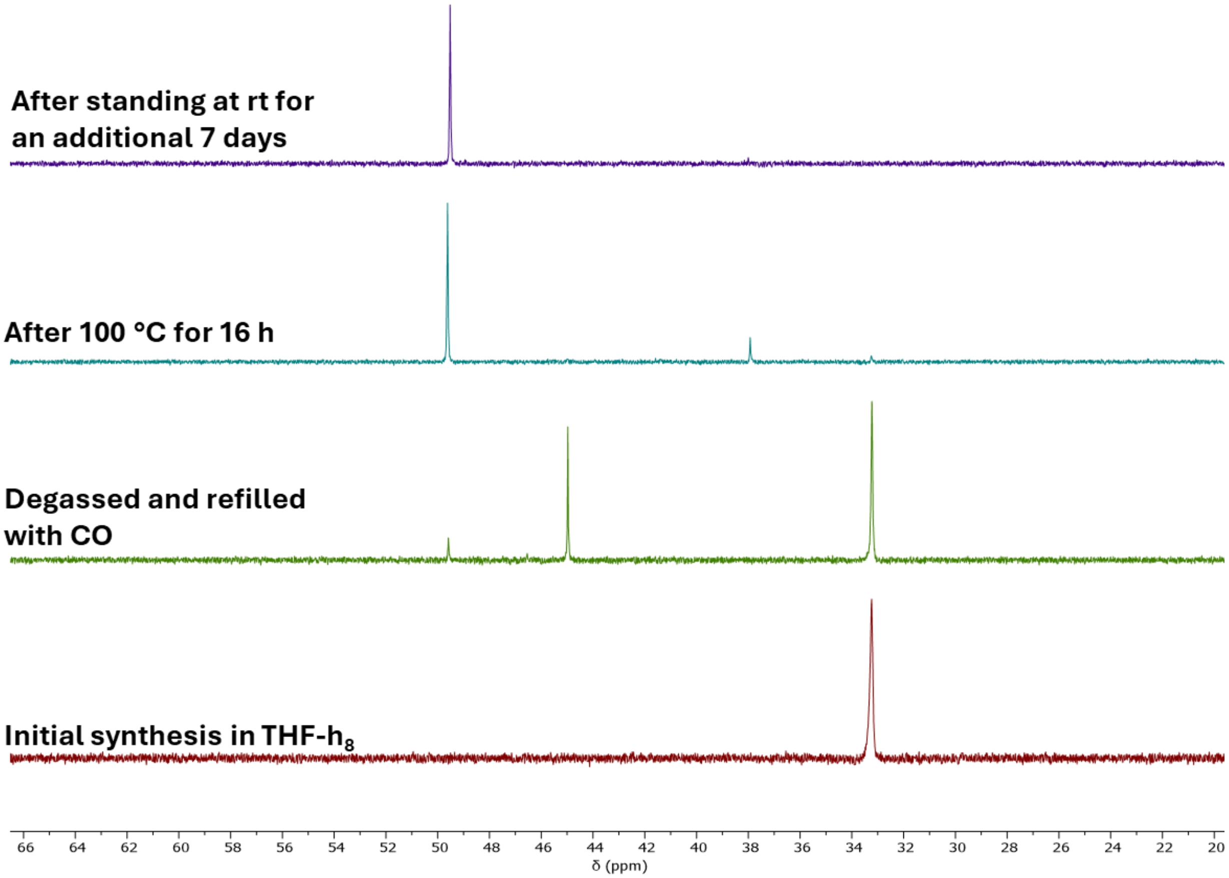

Figure S37. Stacked $^{31}P\{^{1}H\}$ NMR (202 MHz, C_6D_6) spectra of the reaction of the a mixture of **5a** and **5b** with CO.

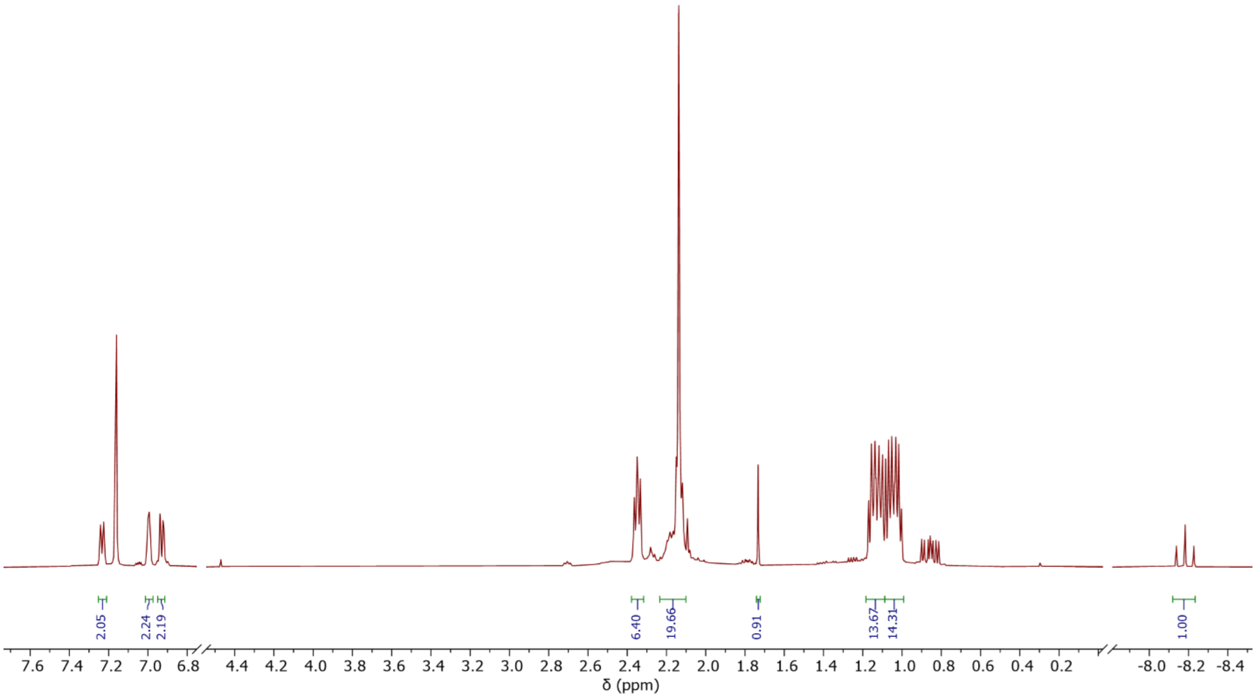

Figure S38. ^{1}H NMR (500 MHz, C_6D_6) spectrum of the reaction of a mixture of **5a** and **5b** with CO and a BH_3 capture agent (DABCO) after heating for 16 h in a 100 °C oil bath. The major products of the reaction are **6** and DABCO•BH_3 (δ 2.36 ppm (t, $J_{H–H}$ = 7 Hz), 2.14 ppm (t $J_{H–H}$ = 7 Hz)), H_2 (δ 4.47 ppm), and approximately 10% of an unidentified side product. The sample contains residual SMe_2 (δ 1.73 ppm).

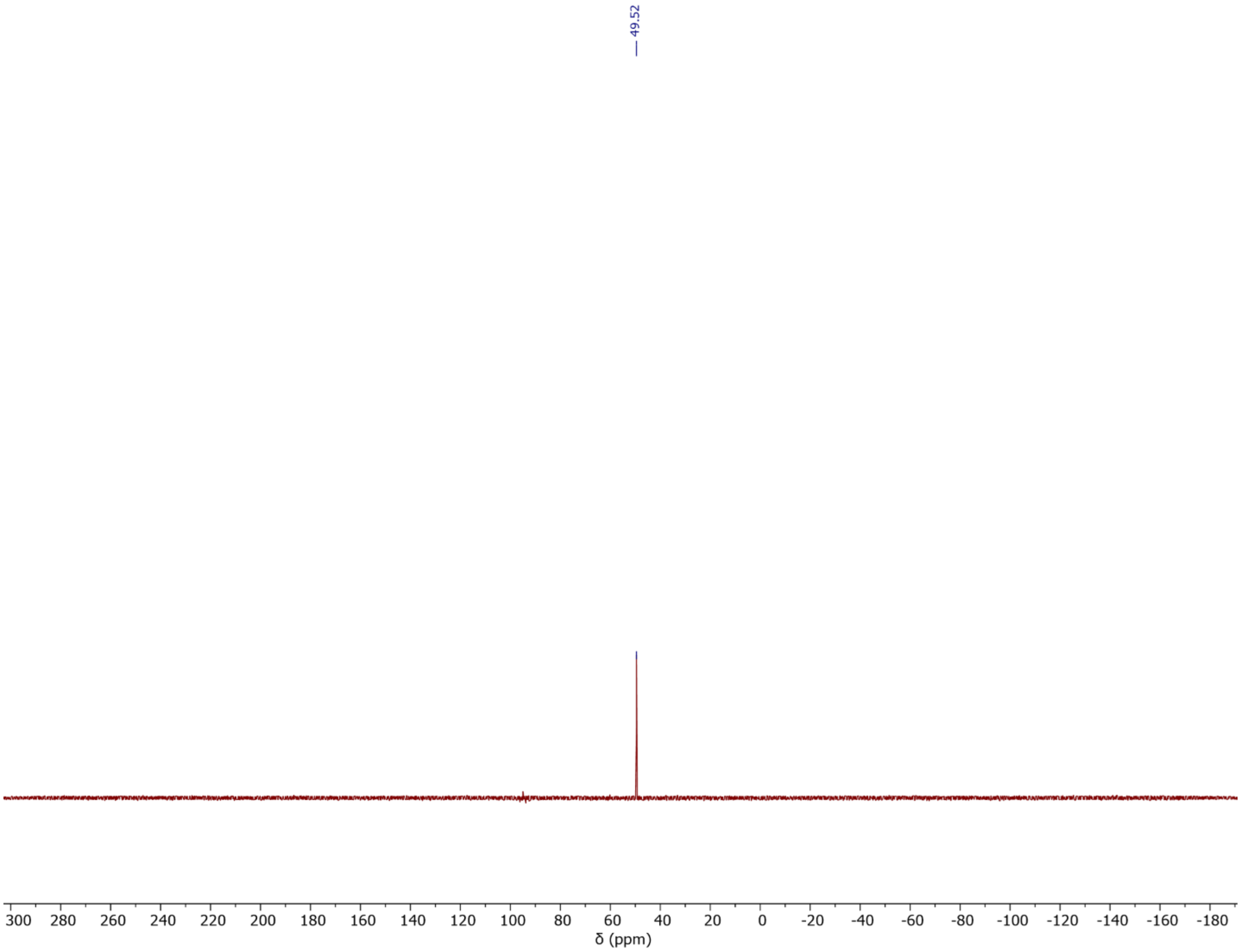

Figure S39. $^{31}P\{^{1}H\}$ NMR (202 MHz, C_6D_6) spectrum of the reaction of a mixture of **5a** and **5b** with CO and a BH_3 capture agent (DABCO) after heating for 16 h in a 100 °C oil bath.

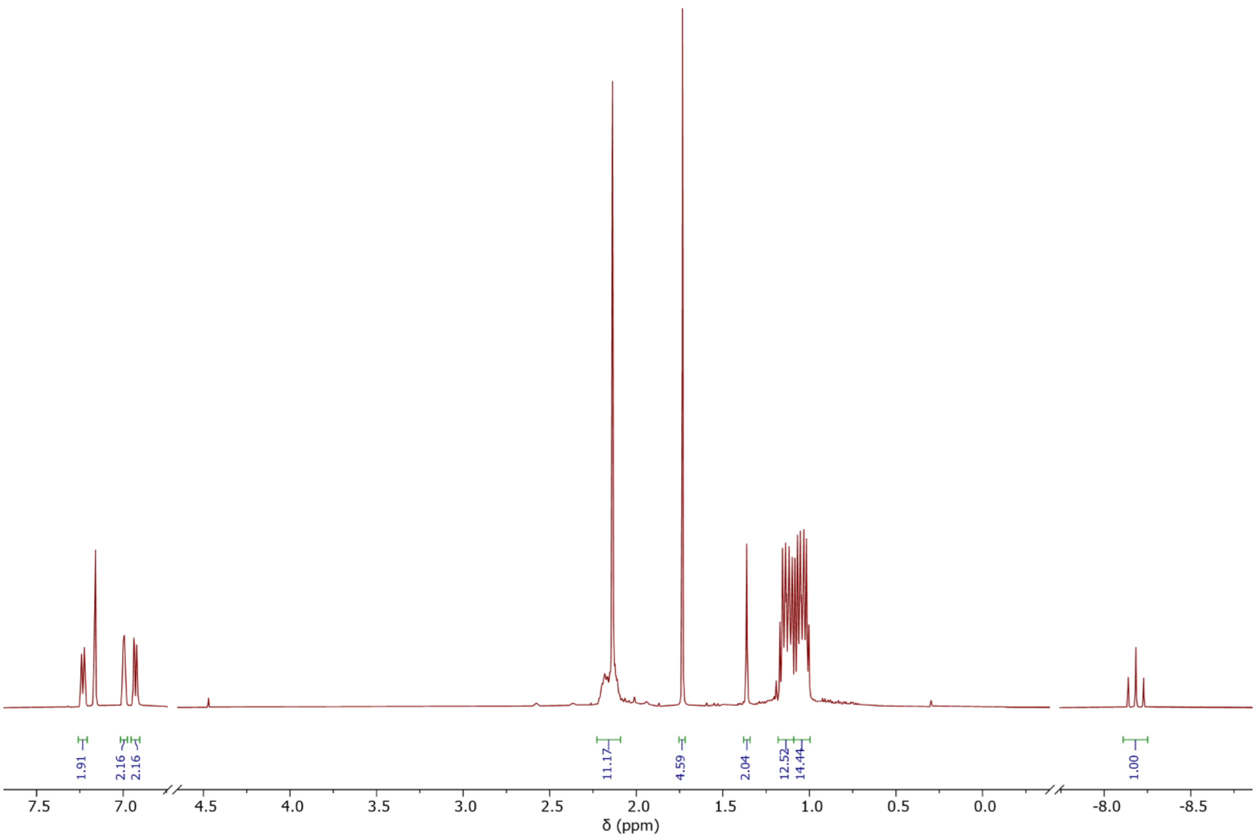

Figure S40. ^{1}H NMR (500 MHz, C_6D_6) spectrum of the reaction of a mixture of **5a** and **5b** with CO and a BH_3 capture agent (SMe_2) after heating for 16 h in a 100 °C oil bath. The major products of the reaction are **6** and SMe_2•BH_3 (δ 1.36 ppm) and H_2 (δ 4.47 ppm). The sample contains residual SMe_2 (δ 1.73 ppm).

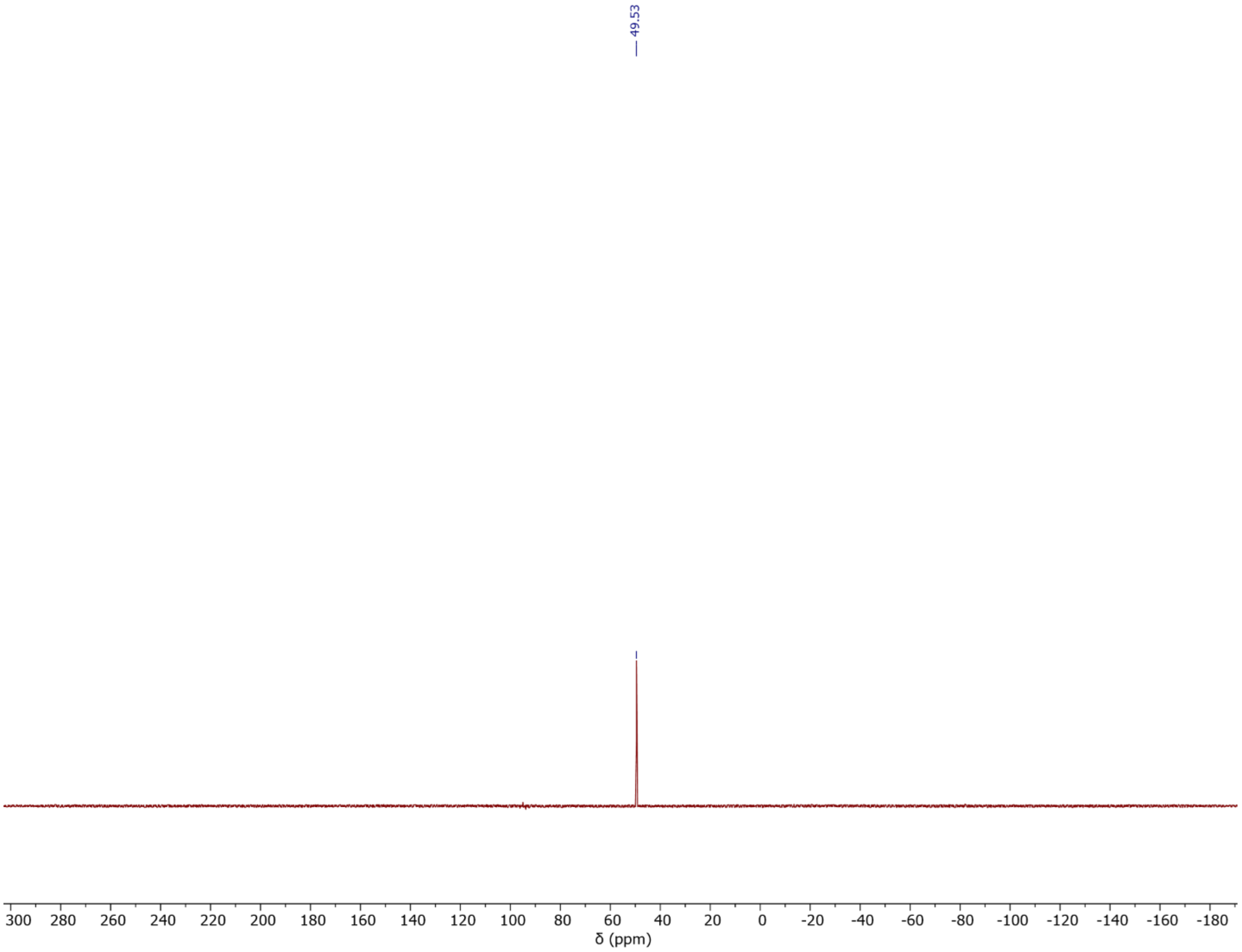

Figure S41. $^{31}P\{^{1}H\}$ NMR (202 MHz, C_6D_6) spectrum of the reaction of a mixture of **5a** and **5b** with CO and a BH_3 capture agent (SMe_2) after heating for 16 h in a 100 °C oil bath.

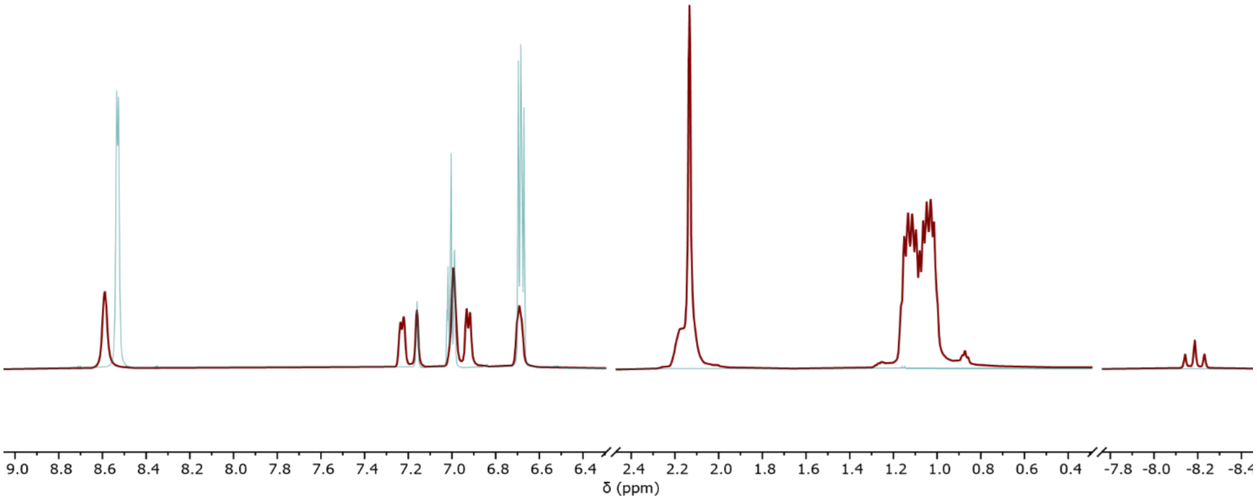

Figure S42. Superimposed ^{1}H NMR (400 MHz, C_6D_6) spectra of pyridine (blue) and the reaction of **6** with pyridine (red). Subtle shifting of the *o*-pyridyl resonance is noted, but no reaction is observed. Sample contains residual pentane.

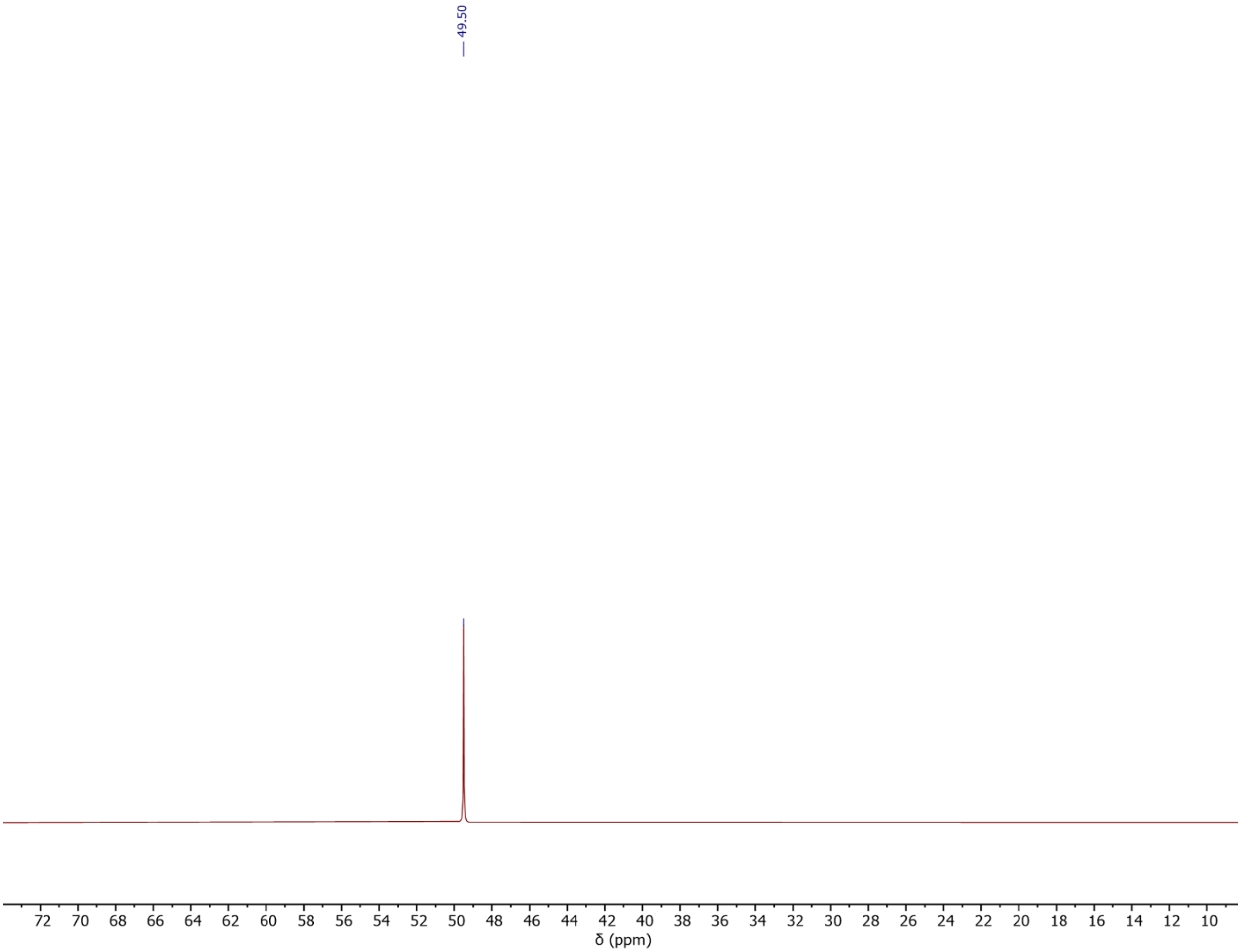

Figure S43. $^{31}P\{^{1}H\}$ NMR (162 MHz, C_6D_6) spectrum of the reaction of **6** and pyridine. Sample contains free **6**.

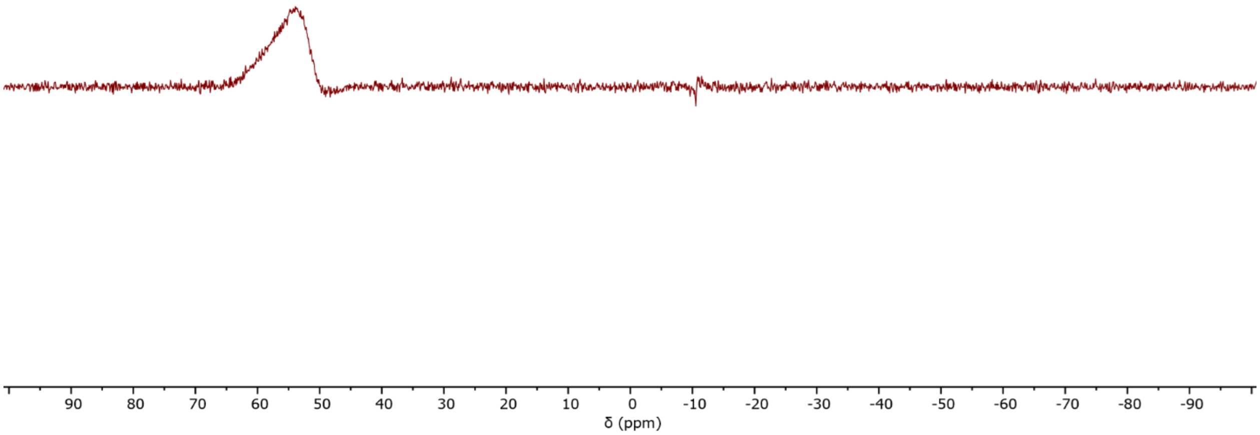

Figure S44. $^{11}B\{^{1}H\}$ NMR (128 MHz, C_6D_6) spectrum of the reaction of **6** and pyridine. Sample contains free **6**.

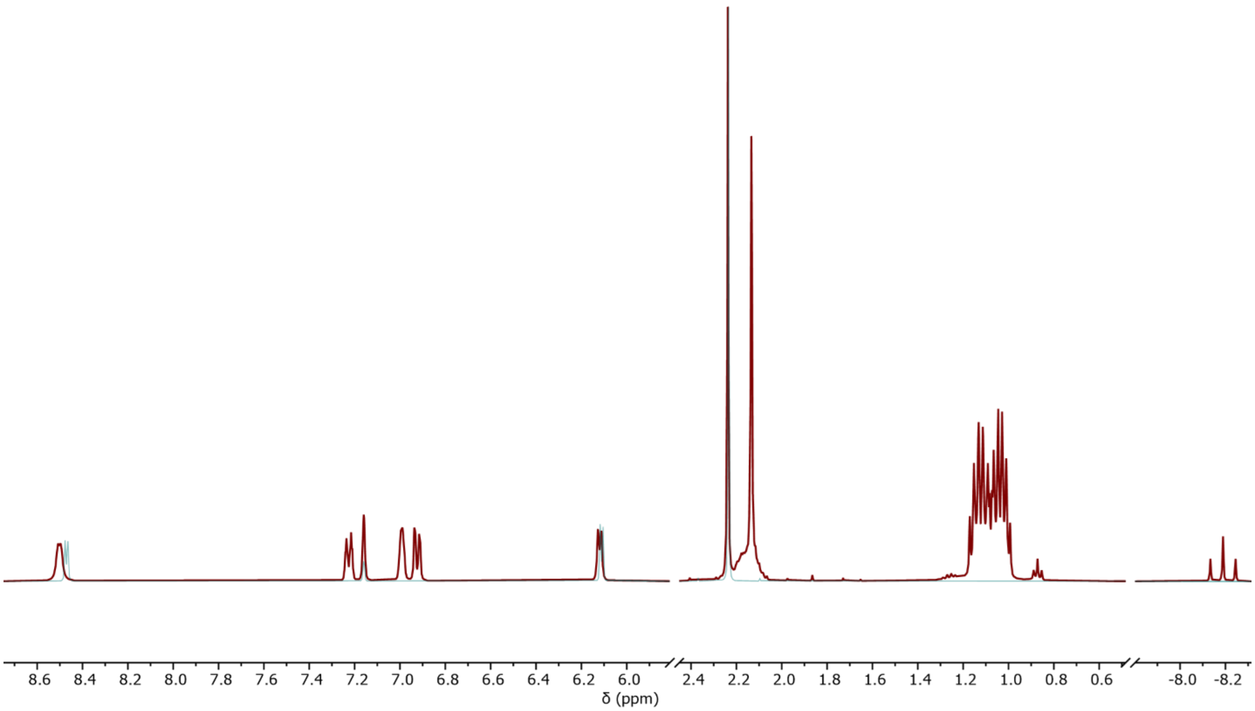

Figure S45. Superimposed ^{1}H NMR (400 MHz, C_6D_6) spectra of DMAP (blue) and the reaction of **6** with DMAP (red). Subtle shifting of the *o*-pyridyl resonance is noted, but no reaction is observed. Sample contains residual pentane.

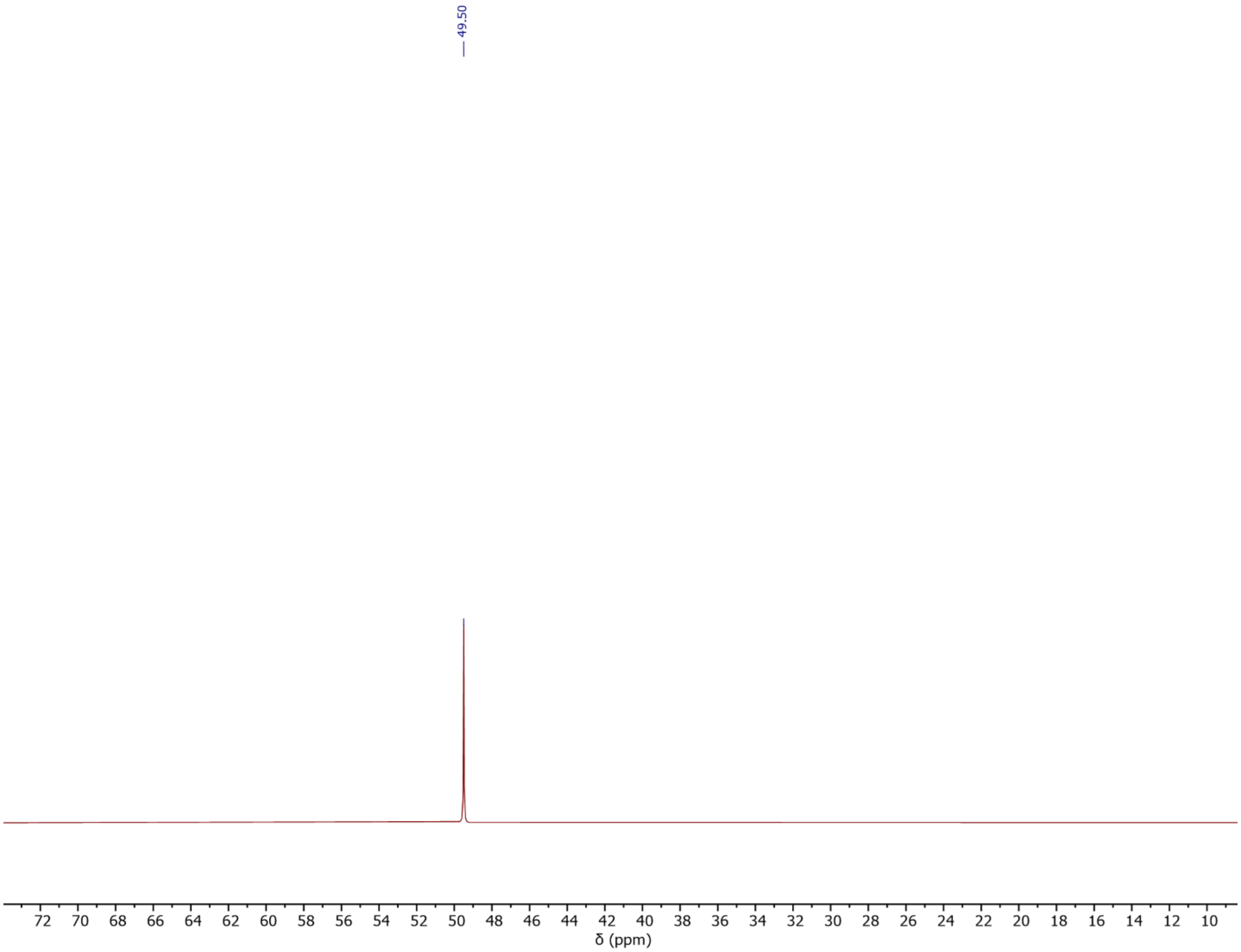

Figure S46. $^{31}P\{^1H\}$ NMR (162 MHz, C_6D_6) spectrum of the reaction of **6** and DMAP. Sample contains free **6**.

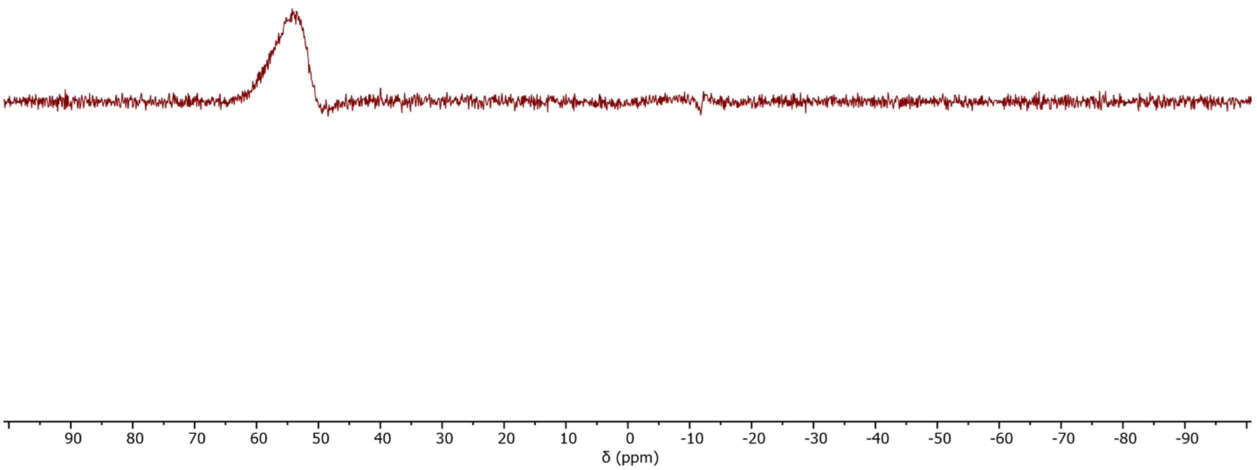

Figure S47. $^{11}B\{^{1}H\}$ NMR (128 MHz, C_6D_6) spectrum of the reaction of **6** and DMAP. Sample contains free **6**.

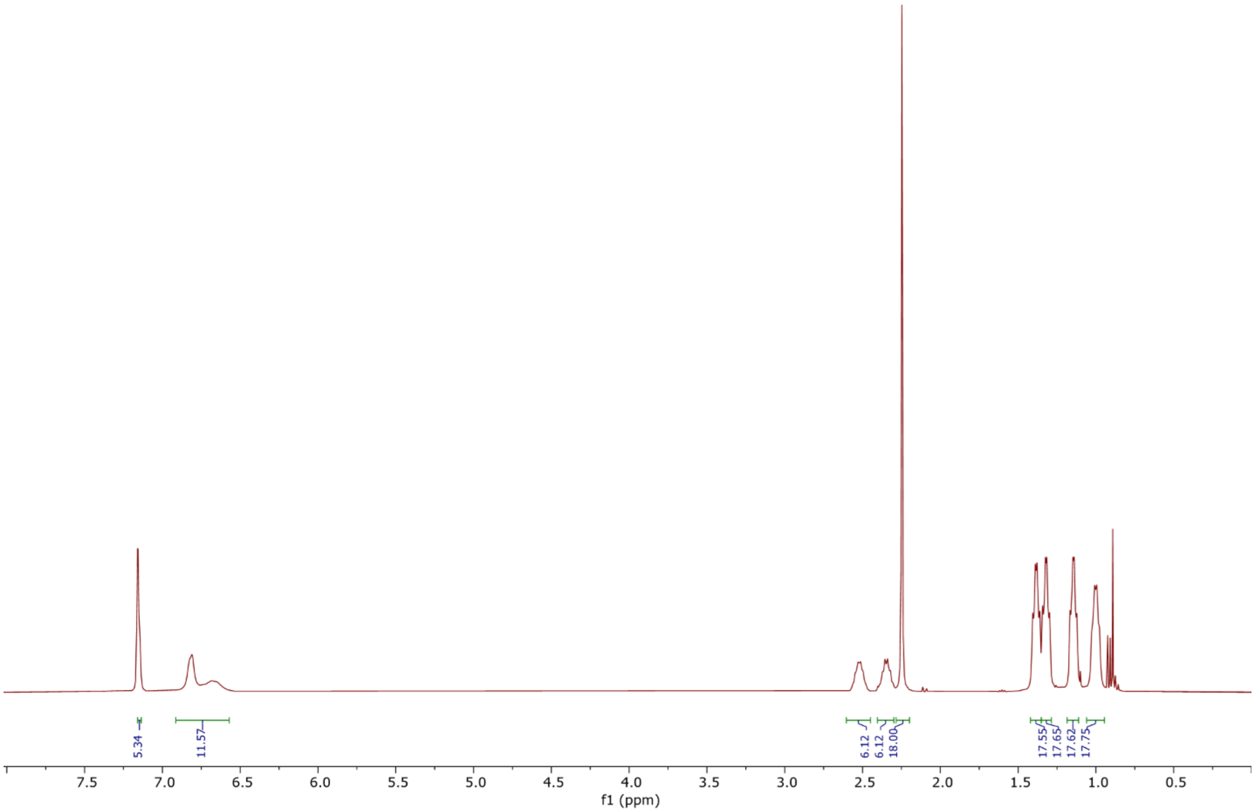

Figure S48. ^{1}H NMR (400 MHz, C_6D_6) spectrum of **7**. Sample contains residual pentane and silicone grease.

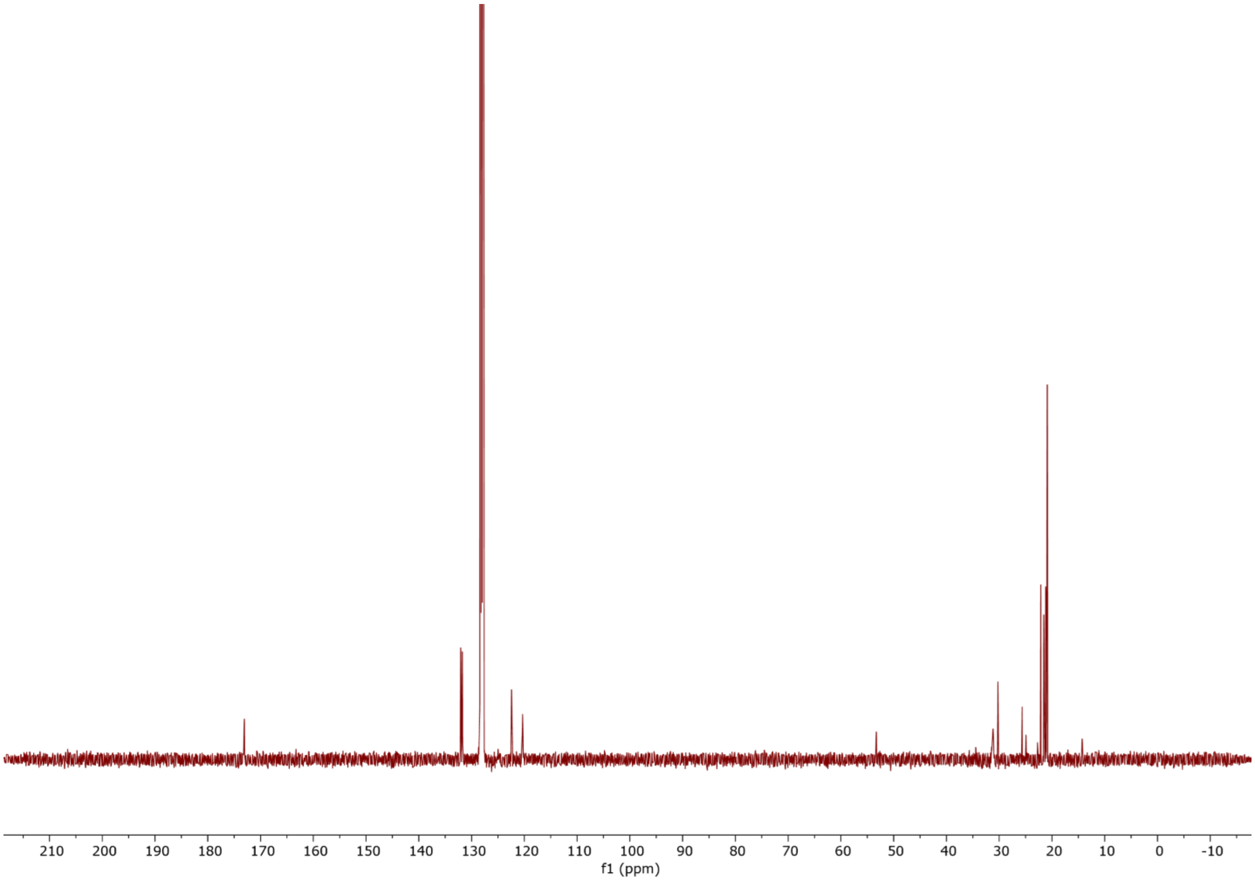

Figure S49. $^{13}C\{^{1}H\}$ NMR (101 MHz, C_6D_6) spectrum of **7.** Sample contains residual pentane and silicone grease.

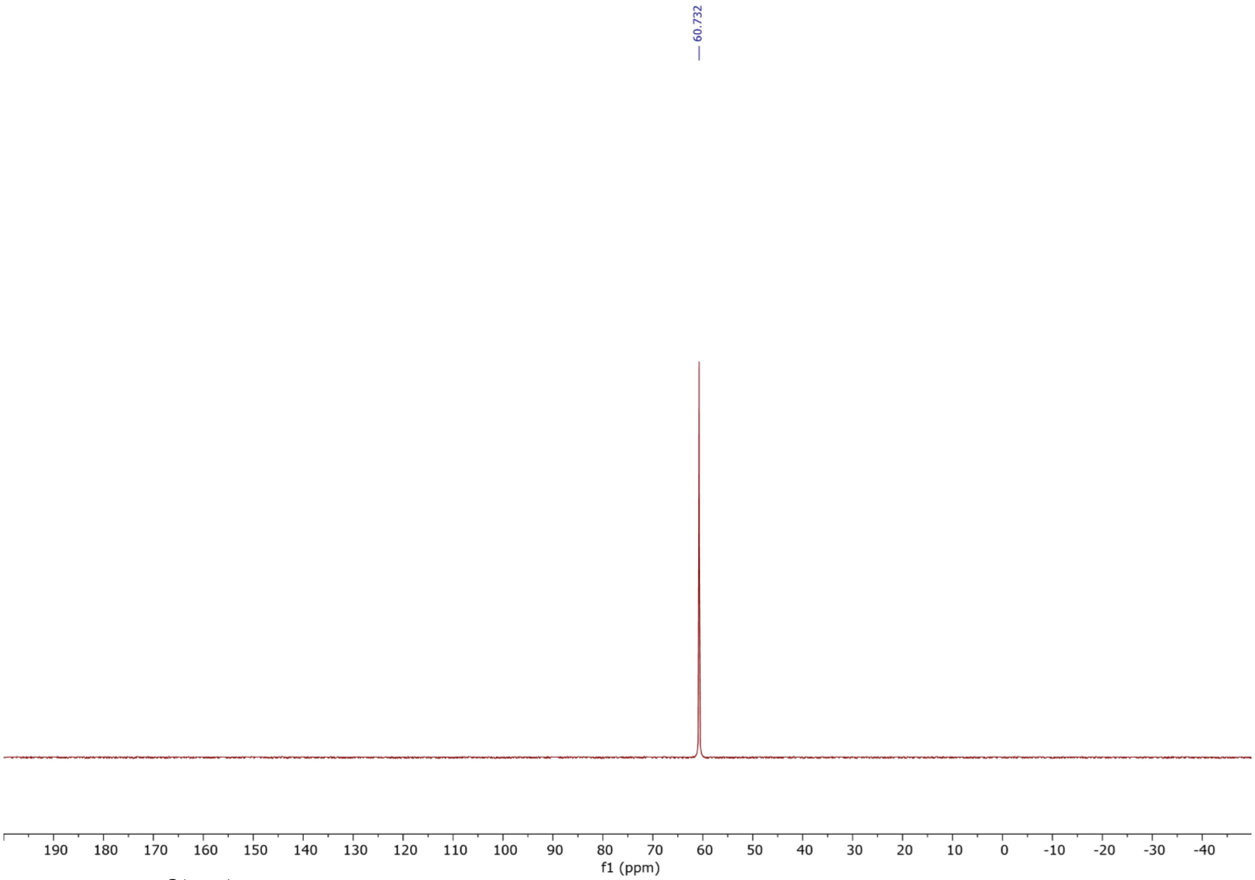

Figure S50. $^{31}P\{^1H\}$ NMR (162 MHz, C_6D_6) spectrum of **7.**

V. Computational Details

All calculations were carried out in Gaussian 16[7] using the B3LYP functional[8,9,10,11] with Grimme dispersion correction (B3LYP-D3).[12] Basis sets used included LANL2DZ for Ru and P, 6-31G(d) for B, C, and O, and 6-31G(d,p) for all hydrogens.[13] The basis set LANL2DZ is the Los Alamos National Laboratory ECP plus a double zeta valence on Ru and P;[14] additional d polarization functions[15] were added to all phosphorus atoms in all DFT calculations. All optimizations were performed in the gas phase with C_1 symmetry and all minima were confirmed by analytical calculation of frequencies, which were also used to compute zero-point energy corrections without scaling. Wiberg Bond Index (WBI) values were calculated within Gaussian 16 using NBO version 3.1.[16]

VI. References

[1] Albers, M. O.; Ashworth, T. V.; Oosthuizen, H. E.; Singleton, E. *Inorg. Synth.* **2009**, *26*, 68-77.

[2] Contreras, J. J.; Lee, S. R.; Nguyen, V. T.; Suarez, T.; Leong, D. W.; Bhuvanesh, N.; Johnson, M. W.; Ozerov, O. V. Transition Metal as Template: Reversing the Synthesis Logic in the Preparation of Pincer Complexes. *Angew. Chem. Int. Ed.* **2025**, *64*, e202418663.

[3] CrysAlisPRO, Rigaku Oxford Diffraction, Poland 2023.

[4] Dolomanov, O. V.; Bourhis, L. J.; Gildea, R. J.; Howard, J. A. K.; Puschmann, H. *J. Appl. Crystallogr.* **2009**, *42*, 339-341.

[5] Sheldrick, G. M. *Acta. Cryst.* **2015**, C*71*, 3-8.

[6] Sheldrick, G. M. *Acta. Cryst.* **2015**, A*71*, 3-8.

[7] Gaussian 16, Revision A.03, Frisch, M. J.; Trucks, G. W.; Schlegel, H. B.; Scuseria, G. E.; Robb, M. A.; Cheeseman, J. R.; Scalmani, G.; Barone, V.; Petersson, G. A.; Nakatsuji, H.; Li, X.; Caricato, M.; Marenich, A. V.; Bloino, J.; Janesko, B. G.; Gomperts, R.; Mennucci, B.; Hratchian, H. P.; Ortiz, J. V.; Izmaylov, A. F.; Sonnenberg, J. L.; Williams-Young, D.; Ding, F.; Lipparini, F.; Egidi, F.; Goings, J.; Peng, B.; Petrone, A.; Henderson, T.; Ranasinghe, D.; Zakrzewski, V. G.; Gao, J.; Rega, N.; Zheng, G.; Liang, W.; Hada, M.; Ehara, M.; Toyota, K.; Fukuda, R.; Hasegawa, J.; Ishida, M.; Nakajima, T.; Honda, Y.; Kitao, O.; Nakai, H.; Vreven, T.; Throssell, K.; Montgomery, J. A., Jr.; Peralta, J. E.; Ogliaro, F.; Bearpark, M. J.; Heyd, J. J.; Brothers, E. N.; Kudin, K. N.; Staroverov, V. N.; Keith, T. A.; Kobayashi, R.; Normand, J.; Raghavachari, K.; Rendell, A. P.; Burant, J. C.; Iyengar, S. S.; Tomasi, J.; Cossi, M.; Millam, J. M.; Klene, M.; Adamo, C.; Cammi, R.; Ochterski, J. W.; Martin, R. L.; Morokuma, K.; Farkas, O.; Foresman, J. B.; Fox, D. J. Gaussian, Inc., Wallingford CT, **2016.**

[8] Becke, D. Density-functional thermochemistry. III. The role of exact exchange, *J. Chem. Phys*. **1993**, *98*, 5648–5652.

[9] Lee, C.; Yang, W.; Parr, R. G. Development of the Colle-Salvetti correlation-energy formula into a functional of the electron density, *Phys. Rev. B.*, **1988,** *37,* 785.

[10] Vosko, S. H.; Wilk, L.; Nusair, M. Accurate spin-dependent electron liquid correlation energies for local spin density calculations: a critical analysis, *Can. J. Phys.* **1980**, *58*, 1200–1211.

[11] Stephens, P. J.; Devlin, F. J.; Chabalowski, C. F.; Frisch, M. J. Ab Initio Calculation of Vibrational Absorption and Circular Dichroism Spectra Using Density Functional Force Fields, *J. Phys. Chem*. **1994,** *98*, 11623–11627.

[12] Grimme, S.; Antony, J.; Ehrlich, S.; Krieg, H. A consistent and accurate ab initio parameterization of density functional dispersion correction (DFT-D) for the 94 elements H-Pu, *J. Chem. Phys*., **2010,** *132*, 154104.

[13] Hariharan, P. C.; Pople, J. A. The influence of polarization functions on molecular orbital hydrogenation energies. *Theor. Chim. Acta* **1973**, *28*, 213-222.

[14] (a) Hay, P. J.; Wadt, W. R. *Ab initio* effective core potentials for molecular calculations. Potentials for the transition metal atoms Sc to Hg. *J. Chem. Phys*. **1985**, *82*, 270-283. (b) Wadt, W. R.; Hay, P. J. *Ab initio* effective core potentials for molecular calculations. Potentials for main group elements Na to Bi. *J. Chem Phys.* **1985**, *82*, 284-298. (c) Hay, P. J.; Wadt, W. R. *Ab initio* effective core potentials for molecular calculations. Potentials for K to Au including the outermost core orbitals. *J. Chem. Phys.* **1985**, *82*, 299-310.

[15] Höllwarth, A.; Böhme, M.; Dapprich, S.; Ehlers, A. W.; Gobbi, A.; Jonas, V.; Köhler, K. F. Stegmann, R.; Veldkamp, A.; Frenking, G. A set of d-polarization functions for pseudo-

potential basis sets of the main group elements Al-Bi and f-type polarization functions for Zn, Cd, Hg. *Chem. Phys. Lett.* **1993**, *208*, 237-240.

[16] NBO Version 3.1, E. D. Glendening, A. E. Reed, J. E. Carpenter, and F. Weinhold.
